# Repeat-Driven Generation of Antigenic Diversity in a Major Human Pathogen, *Trypanosoma cruzi*

**DOI:** 10.3389/fcimb.2021.614665

**Published:** 2021-03-03

**Authors:** Carlos Talavera-López, Louisa A. Messenger, Michael D. Lewis, Matthew Yeo, João Luís Reis-Cunha, Gabriel Machado Matos, Daniella C. Bartholomeu, José E. Calzada, Azael Saldaña, Juan David Ramírez, Felipe Guhl, Sofía Ocaña-Mayorga, Jaime A. Costales, Rodion Gorchakov, Kathryn Jones, Melissa S. Nolan, Santuza M. R. Teixeira, Hernán José Carrasco, Maria Elena Bottazzi, Peter J. Hotez, Kristy O. Murray, Mario J. Grijalva, Barbara Burleigh, Edmundo C. Grisard, Michael A. Miles, Björn Andersson

**Affiliations:** ^1^ Department of Cell and Molecular Biology, Karolinska Institutet, Stockholm, Sweden; ^2^ European Bioinformatics Institute, Wellcome Sanger Institute, Hinxton, United Kingdom; ^3^ Faculty of Infectious and Tropical Diseases, London School of Hygiene and Tropical Medicine, London, United Kingdom; ^4^ Departamento de Parasitologia, Universidade Federal de Minas Gerais, Belo Horizonte, Brazil; ^5^ Departamento de Biologia Celular, Embriologia e Genética, Universidade Federal Santa Catarina, Florianópolis, Brazil; ^6^ Departamento de Parasitología, Instituto Conmemorativo Gorgas de Estudios de la Salud, Ciudad de Panamá, Panama; ^7^ Grupo de Investigaciones Microbiológicas-UR (GIMUR), Departamento de Biología, Facultad de Ciencias Naturales, Universidad del Rosario, Bogotá, Colombia; ^8^ Grupo de Investigaciones en Microbiología y Parasitología Tropical (CIMPAT), Tropical Parasitology Research Center, Universidad de Los Andes, Bogotá, Colombia; ^9^ Centro de Investigación para la Salud en América Latina (CISeAL), Escuela de Ciencias Biológicas, Pontificia Universidad Católica del Ecuador, Quito, Ecuador; ^10^ Sabin Vaccine Institute and Texas Children’s Hospital Center for Vaccine Development, National School of Tropical Medicine, Department of Pediatrics - Tropical Medicine, Baylor College of Medicine, Houston, TX, United States; ^11^ Departamento de Bioquímica e Imunologia, Universidade Federal de Minas Gerais, Belo Horizonte, Brazil; ^12^ Laboratorio de Biología Molecular de Protozoarios, Instituto de Medicina Tropical, Facultad de Medicina, Universidad Central de Venezuela, Caracas, Venezuela; ^13^ Department of Biomedical Sciences, Heritage College of Osteopathic Medicine, Infectious and Tropical Disease Institute, Ohio University, Athens, OH, United States; ^14^ Department of Immunology and Infectious Diseases, T.H. Chan School of Public Health, Harvard University, Boston, MA, United States; ^15^ Departamento de Microbiologia, Imunologia e Parasitologia, Universidade Federal Santa Catarina, Florianópolis, Brazil

**Keywords:** *Trypanosoma cruzi*, genome sequence, antigenic variation, population genetics, parasitology, microbial genomics, tropical medicine, pathology of infectious diseases

## Abstract

*Trypanosoma cruzi*, a zoonotic kinetoplastid protozoan parasite, is the causative agent of American trypanosomiasis (Chagas disease). Having a very plastic, repetitive and complex genome, the parasite displays a highly diverse repertoire of surface molecules, with pivotal roles in cell invasion, immune evasion and pathogenesis. Before 2016, the complexity of the genomic regions containing these genes impaired the assembly of a genome at chromosomal level, making it impossible to study the structure and function of the several thousand repetitive genes encoding the surface molecules of the parasite. We here describe the genome assembly of the Sylvio X10/1 genome sequence, which since 2016 has been used as a reference genome sequence for *T. cruzi* clade I (TcI), produced using high coverage PacBio single-molecule sequencing. It was used to analyze deep Illumina sequence data from 34 *T. cruzi* TcI isolates and clones from different geographic locations, sample sources and clinical outcomes. Resolution of the surface molecule gene distribution showed the unusual duality in the organization of the parasite genome, a synteny of the core genomic region with related protozoa flanked by unique and highly plastic multigene family clusters encoding surface antigens. The presence of abundant interspersed retrotransposons in these multigene family clusters suggests that these elements are involved in a recombination mechanism for the generation of antigenic variation and evasion of the host immune response on these TcI strains. The comparative genomic analysis of the cohort of TcI strains revealed multiple cases of such recombination events involving surface molecule genes and has provided new insights into *T. cruzi* population structure.

## Introduction

*Trypanosoma cruzi* is a kinetoplastid protozoan and the etiologic agent of Chagas disease, considered one of the most important human parasitic disease in Latin America. The Global Burden of Disease Study 2013 reported that almost 7 million people live with Chagas disease in the Western Hemisphere (GBD 2013 Mortality and Causes of Death Collaborators, 2015), with the expectation that up to one third will progress to develop chronic chagasic cardiomyopathy (CCC) or other life-threatening symptoms. In 2015, 5,742,167 people were estimated to be infected with *T. cruzi* in 21 Latin American countries and around 13% of the Latin American population is at risk of contracting *T. cruzi* infection due to domicile infestation of triatomine bugs or due to non-vectorial transmission *via* blood transfusion, organ transplant, oral, congenital or accidental infection (“WHO | 6 February 2015, Vol. 90, 6 (pp. 33–44)” 2015). Human Chagas disease is not restricted to Latin America. The migration of infected humans to non-endemic areas has made it a new public health threat in other geographic areas such as North America, Europe and Asia (Bern, 2015). Also, sylvatic *T. cruzi* transmission cycles, often associated with human disease, have been described in areas formerly considered as free from this disease such as in Texas (USA) (Bern, 2015).

The acute phase of the disease frequently lacks specific symptoms, is often undiagnosed and usually resolves in a few weeks in immunocompetent individuals but may be fatal in around 5% of diagnosed cases. Without successful treatment, a *T. cruzi* infection is normally carried for life. The disease progresses to either a chronic indeterminate phase that is asymptomatic, or to a chronic symptomatic phase with severe clinical syndromes such as cardiomyopathy, megaesophagus and/or megacolon (Rassi et al., 2010); meningoencephalitis may occur, especially in immunocompromised patients (Bern, 2015). The current prolonged chemotherapy (benznidazole or nifurtimox) is mostly effective only in the acute phase, particularly because severe side effects may interrupt treatment of adults in the chronic phase. There is currently no effective treatment for advanced chagasic cardiomyopathy (Morillo et al., 2015), and there is an urgent need to identify new potential drug and vaccine targets (Pecoul et al., 2016).

*T. cruzi* infection is a zoonosis, and the parasite has a complex life cycle; where transmission to humans occurs most frequently by contamination with infected feces from triatomine insect vectors (Subfamily Triatominae). The parasite evades the immune responses with the aid of multiple surface molecules from three large diverse gene families (Trans-Sialidases, Mucins and Mucin-Associated Surface Proteins - MASPs), which are also involved in cell invasion and possibly pathogenicity (Schenkman et al., 1994; Frasch, 2000; Yoshida and Cortez, 2008; Osorio et al., 2012).

Six distinct genetic clades of *T. cruzi* have been recognized, named TcI to TcVI (Discrete Typing Units or DTU-I to VI). The first genome sequence for *T. cruzi* was produced using Sanger sequencing technology from a hybrid, highly polymorphic, TcVI strain. The resultant genome sequence, while extremely useful for the core regions of the genome, was highly fragmented, especially in repetitive regions (El-Sayed et al., 2005). This sequence has been improved using enhanced scaffolding algorithms, but many repetitive regions remain unresolved (Weatherly et al., 2009). Subsequently, FLX 454 Titanium and Illumina sequencing were used to sequence a less polymorphic TcI strain (Sylvio X10/1), which allowed the first comparative genomic studies of *T. cruzi*, but correct assembly of repetitive regions was still impossible (Franzén et al., 2011; Franzén et al., 2012). The thousands of related genes that code for the surface proteins are generally located in large multigene family clusters of the *T. cruzi* genome (Kim et al., 2005), in the form of extremely repetitive segments with multiple gene copies and pseudogenes. These multigene family clusters are distinct from the core regions of the genome, defined as regions that share gene content and synteny with the genomes of other trypanosomatids (Llewellyn et al., 2009). The repetitive nature of the tandem arrays, and the length of the repeats, made correct assembly impossible using short and medium-sized sequence reads. The available *T. cruzi* genome sequences were therefore incomplete and inaccurate in these important regions, making it impossible to study the complex surface gene families in contrast to conserved core genomic regions (Berná et al., 2018).

We made a near-complete reconstruction of the majority (~98.5% of the estimated genome size) of the *T. cruzi* TcI Sylvio X10/1 genome available in Genbank and TriTrypDB in 2016, and it was described in a preprint made available in June 2018 (Talavera-López et al., 2018). This sequence has served as a main genome sequence for *T. cruzi* clade 1, since it was made public. We were able to decipher the majority of the organisation of *T. cruzi* surface protein coding gene repertoire from the TcI Sylvio X10/1 strain, revealing large numbers of evenly spaced retrotransposons, which may play a role in generating genomic structural diversity and antigenic variation. This study has been followed by several others using similar approaches and parasite strains (Callejas-Hernández et al., 2018; Reis-Cunha et al., 2018; Schwabl et al., 2019).

The population structure of *T. cruzi* is complex, and there is a high degree of genetic and phenotypic variation. The current TcI to TcVI clades are based on biochemical and molecular markers (Zingales et al., 2012), although there is substantial diversity even within these six groups (Llewellyn et al., 2009). The TcI clade is widespread and can be found across the American continent, and has been associated with CCC (Ramírez et al., 2010) and sudden death (Bern et al., 2011; Montgomery et al., 2014), among other clinical manifestations. In conjunction with the Sylvio X10/1 genome sequence, we generated Illumina whole-genome sequencing data for 34 *T. cruzi* TcI isolates and clones from different geographic locations for comparative analyses. These data was used to carry out population genetics studies, where strains from different environments and geographic locations were compared. We found patterns of active recombination possibly associated with the generation of new surface molecule variants. These studies contributed to answering longstanding questions on the biology of Chagas disease and host-parasite interaction in general. The availability of the close to complete repertoire of genes encoding surface molecules allows further research on virulence and pathogenesis, as well as the identification of drug targets and vaccine candidates, focused on shared and conserved motifs present within these variable families.

## Materials and Methods

### Genome Sequencing and Assembly

The *Trypanosoma cruzi* Sylvio X10/1 strain was isolated from an acute human case of Chagas disease in Brazil. Total genomic DNA of this TcI strain was obtained from culture epimastigotes as formerly described ^11^ and used to produce PacBio CCS data according to standard protocols from the Genomic Facility of Science for Life Laboratory (Sweden) and Pacific Biosciences (USA). Genomic DNA was sequenced to a depth of 210X using the PacBio platform, supplying raw reads with an average length of 5.8 Kb. These reads were corrected by means of the PBcR v8.3 pipeline with the MHAP algorithm (Berlin et al., 2015) using the auto-correction parameters described to merge haplotypes and skipping the assembly step, producing a total of 1,216 contigs (NG50 = 62 Kb). Illumina sequences at an average coverage of approximately 120X, with a mean read length of 101 bp were added. The reads were trimmed from adaptors and filtered using the Nesoni utility (https://github.com/Victorian-Bioinformatics-Consortium/nesoni), which is now part of Tail Tools (https://github.com/Monash-RNA-Systems-Biology-Laboratory/tail-tools) in order to remove bases with a quality score < 20 and length < 75.

Later, the assembly was scaffolded using the corrected PacBio reads with the SSPACE-Long scaffolder yielding 310 scaffolds (NG50 = 788 Kb); 118 gaps were filled using Illumina reads with GapFiller and corrected PacBio reads with PBJelly2. Finally, the core regions of these scaffolds were aligned against the core regions of the TcVI CL Brener reference genome using ABACAS (http://abacas.sourceforge.net), producing 47 scaffolds, henceforth designated as chromosomes. The quality of the new assembly was assessed with FRC_bam with the Illumina paired end reads generated at the Genomic Facility of Science for Life Laboratory (Sweden) using the same genomic DNA extraction used for PacBio sequencing. The final genome size was 41382871 bp in 47 scaffolds. The number of gaps was 1005, and they are indicated by rows of Ns in the sequence.

### Annotation of the *Trypanosoma cruzi* Sylvio X10/1 Genome

The genome sequence was annotated using a new kinetoplastid genome annotation pipeline combining homology-based gene model transfer with *de novo* gene prediction. To allow for the sensitive identification of partial genes, input sequences were split at stretches of undefined bases, effectively creating a set of ‘pseudocontigs’, each of which does not contain any gaps. Gene finding was then performed on both the original sequences and the pseudocontigs using AUGUSTUS, which also calls partial genes at the boundaries of each pseudocontig. The minimum ORF length that was considered for annotation was 50 amino acids to allow for the identification of short peptides that were supported by a contig at least twice the length of a read. AUGUSTUS models were trained on 800 genes randomly sampled from the 41 Esmeraldo-type (TcII) *T. cruzi* CL Brener chromosomes in GeneDB. Protein-DNA alignments of reference proteins against the new *T. cruzi* sequences, generated using Exonerate, were additionally used to improve the accuracy of the gene prediction. In addition, the RATT software was used to transfer highly conserved gene models from the *T. cruzi* CL Brener annotation to the target. A non-redundant set of gene models was obtained by merging the results of both RATT and AUGUSTUS and, for each maximal overlapping set of gene models, selecting the non-overlapping subset that maximizes the total length of the interval covered by the models, weighted by varying levels of *a priori* assigned confidence. Spurious low-confidence protein coding genes with a reading direction in disagreement with the directions of the polycistronic transcriptional units were removed automatically. The result of this integration process was then merged with ncRNA annotations produced by specific tools such as ARAGORN and Infernal. Finally, protein-DNA alignments with frame shifts produced by BLAST were used in a computational approach to identify potential pseudogenes in the remaining sequence.

Downstream of the structural annotation phase, gene models were automatically assigned IDs and further extended with product descriptions and GO terms, both transferred from CL Brener orthologs and inferred from Pfam protein domain hits and represented as feature attributes or Sequence Ontology-typed subfeatures tagged with appropriate evidence codes. This annotation pipeline has been implemented in the Companion web server. The assembled genome was scanned for small RNAs using INFERNAL against the curated RFAM database using cmsearch with a minimum e-value of 1 ×10^-10^, a GC-bias of 0 and a minimum alignment length of 10 nt. This annotation process has been implemented into the web-based annotation pipeline COMPANION (Steinbiss et al., 2016) from the Wellcome Trust Sanger Institute.

Repetitive sequences were annotated using RepeatMasker with the NCBI+ search engine and LTRHarvest. Using the genomic coordinates of the repetitive elements, the genome was split in windows of 10 Kb to identify VIPER and L1Tc retroelements adjacent to surface molecule genes (i.e: trans-sialidases, mucins and MASP). A one-sided Fisher’s exact test was used to evaluate if the retroelements were enriched in genomic segments containing surface molecule genes.

### Identification of Single Nucleotide Polymorphism (SNP) and Insertion/Deletion Events (Indels)

An improved short-read mapping strategy was used to assign the reads to their target sequences with high accuracy, especially in regions rich in simple and low complexity repeats, by taking advantage of the statistical read placement implemented in the Stampy read mapper to accurately call genomic variants from the mapped reads. Reads from all 34 *T. cruzi* TcI isolates (SRA BioProject accession number: PRJNA325924) were mapped against the assembled *T. cruzi* Sylvio X10/1 genome using a two-step mapping process to improve the mapping of Illumina data to highly repetitive regions: First, reads were mapped using BWA MEM with default parameters; later, the BAM file produced by BWA was remapped with Stampy (v1.23) using the *–bamkeepgoodreads* option. The final mapping file was sorted and filtered for PCR duplicates using Picard Tools v1.137. Variants were called using FreeBayes with a minimum per-base quality of 30, minimum mapping quality of 30 and minimum coverage of 15 bases. Variants that were found in a potentially misassembled region were excluded from the analysis. Additionally, genomic variants were called using FermiKit—which is an assembly-based variant caller—to validate the genomic variants observed in subtelomeric regions. A consensus of the two methods was used as a final set of variants for downstream analyses. Haplotypes were phased using Beagle r1399. The phased markers were used for downstream analyses with SNPrelate and VCFtools and the functional effect of the identified variants was predicted using SnpEff.

### Identification of Genomic Structural Variants (SV)

Genomic structural variants were identified within TcI isolates, using a consensus of different methods: Delly2, Lumpy, FermiKit and FindTranslocations (https://github.com/vezzi/FindTranslocations.git) using both raw reads and realigned BAM files. For each method, an SV must had a depth of coverage > 10 reads and a mapping quality of > 30. Later, a consensus was created with all the SV that were supported by all the methods. SVs that were supported by FermiKit and at least one of the mapping-based methods were also included but labeled as ‘Low Confidence’. SVs identified by only one method were not included. Breakpoint analysis was done with custom Python scripts and their functional effect was predicted using SNPeff. Analyses of copy number variation (CNV) were done using the BAM files for each sample with the *Control-FREEC* package. Determination of the fixation index (*Fst*) using VCFTools was carried out for the *T. cruzi* CG and FcHc clones obtained from human TcI isolates from Colombia (**Supplementary Table 1**). For each strain, replicate clones from the original sample were isolated and cultured under the same conditions, and five of the replicates from each sample were sequenced in the Illumina HiSeq 2500 run.

## Results

### Genome Sequence of *Trypanosoma cruzi* Sylvio X10/1

**Figure 1:**
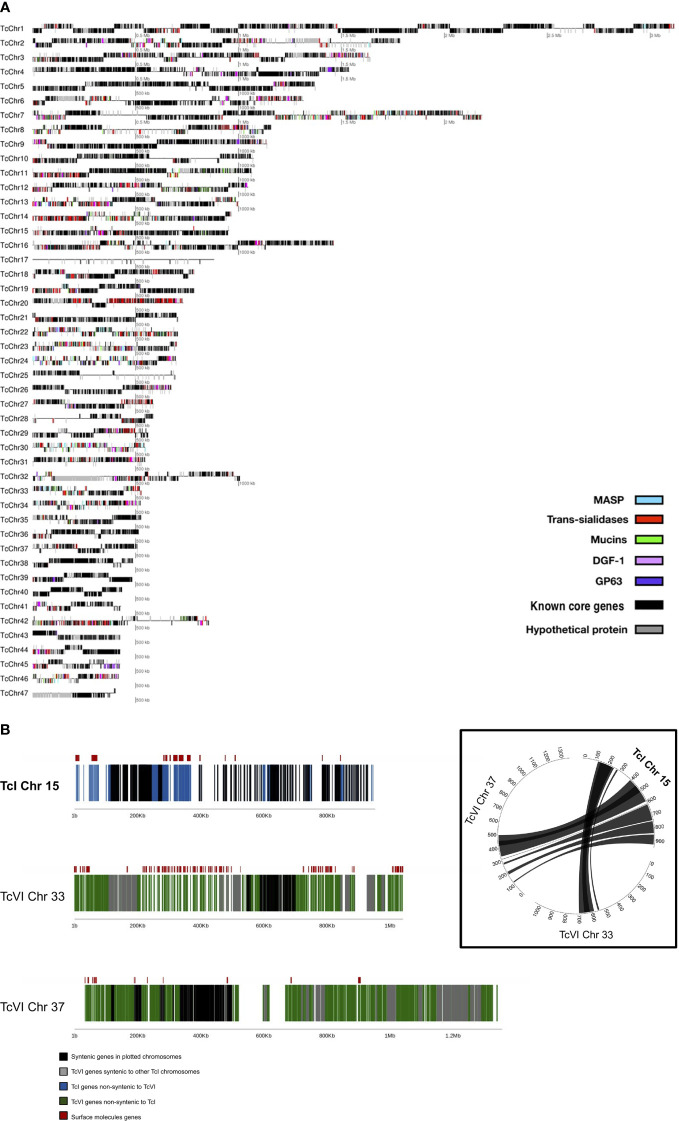
**(A)** Distribution of surface molecule gene tandem arrays in the 47 chromosomes of the TcI Sylvio X10/1 genome. In this image, each line corresponds to an assembled putative chromosome drawn in proportion to its size, where genes corresponding to the largest *T. cruzi* multigene families are represented by colored boxes, hypothetical genes are represented by gray boxes and known core genes as black boxes. The position of the gene boxes above or below the line corresponds to the direction of transcription. **(B)** Comparison of chromosome 15 from the TcI Sylvio X10/1 assembly with TcVI CL Brener chromosomes containing syntenic blocks. The TcI (Sylvio X10/1) chromosome is depicted with black boxes indicating genes that have a CL Brener homolog, and blue boxes showing genes with no synteny with CL Brener. For TcVI (CL Brener) green boxes indicate genes that have no Sylvio X10/1 homolog. The red boxes above these lines show the location of complete genes coding for large surface molecule gene family members. In the circle plot, the lines between chromosomes represent regions of synteny between orthologous genes.

The final Sylvio X10/1 (TcI) genome assembly reconstructed 98.5% of the genome size, as previously estimated by analyzing gene content, and was contained in 47 chromosomes (**Figure 1A**) assembled from 210 X PacBio sequence data and a previous Illumina data set (**Table 1**). We tentatively refer to these as chromosomes in this paper, even though more verification of the complete karyotype is needed. Reads corresponding to mitochondrial DNA, kDNA, were removed by homology searches and were thus not included in the analysis. Comparison with the available short read assembly of the TcVI strain CL Brener revealed conserved core syntenic blocks composed of stretches of homologous sequences separated by non-syntenic regions (**Figure 1B** and **Supplementary Table 2**) that corresponded to regions that were in some cases initially not reconstructed in the hybrid TcVI strain, but have been partially resolved in later versions of this genome sequence. The non-syntenic regions mostly contained surface molecule gene arrays, and other repeated regions. In some cases, we found possible other gene-rich regions that were expanded in longer CL Brener chromosomes (**Figure 1B**). The length of the PacBio reads and the high coverage allowed the reconstruction of long stretches of repetitive sequences in the Sylvio X10/1 genome that could previously not be resolved using shorter read data for this genome.

**Table 1:** *Trypanosoma cruzi* Sylvio X10/1 strain (Tc-I) genome assembly.

Metric	Value
Genome size	41.3 Mbp
Number of scaffolds	47
Percentage of reconstruction	98.5%
Longest scaffold	3.1 Mbp
Shortest scaffold	404 Kb
NG50	1.0 Mbp
N50	1.1 Mbp

The coverage of genomic regions coding for surface molecules was similar to that of known non-repetitive regions, which supported the correct reconstruction of these areas with a limited amount of assembly errors. To further investigate the quality of the new assembly, Illumina short reads were mapped and analyzed with FRC_bam, which revealed assembly artefacts related to low coverage, wrong paired-end read orientation, and higher than expected sequencing coverage in regions with long stretches of simple repeats. Coverage data based on mapping Illumina reads to the final genome sequence is presented in **Supplementary Figures 1** and **2**.

Repetitive elements comprised 18.43% of the TcI Sylvio X10/1 genome, 2.18% of which could not be classified using the repeat databases. LINE retroelements of the R1/Jockey group (3.63%) and VIPER LTRs (2.87%) were found to be the most prevalent types of retroelements, covering 6.89% of the genome, which is much higher than the 2.57% estimated from the previously published Sylvio X10/1 draft assembly using short reads (Franzén et al., 2012).

Although retrotransposons were found to be present throughout the genome, the frequency of VIPER and L1Tc elements was markedly higher in multigene family-rich regions and they were found within one kilobase of pseudogenes, hypothetical proteins and surface molecule gene tandem arrays (One-sided Fisher exact test, *p-value* < 1.32 × 10^-16^). This distribution indicates that these elements may contribute to increased recombination activity in the gene family clusters by providing a source of microhomology. We do not have experimental evidence for the activity of these retroelements in *T. cruzi* and it is unknown if they directly affect gene expression.

Simple and low complexity repeats were observed surrounding surface molecule coding sequences and were also more abundant in the multigene family regions (2.18%), extending up to 4 Kb, compared to core regions (0.98%) where they were much shorter (10–120 bp). The most prevalent type of simple repeat had the (C)n motif (11.7%), (TG)n repeat motif (5.6%) and (CA)n repeat motif (5.1%); each variable in length. The microhomology of these simple subtelomeric repeats may facilitate recombination resulting in new surface molecule variants, as described in other parasitic protozoa, including *Trypanosoma brucei* and *Plasmodium falciparum* (Hall et al., 2013; Claessens et al., 2014). However, it is noteworthy to mention that such subtelomeric regions are far less complex and shorter in *T. brucei* (African, virulent) and *T. rangeli* (Stoco et al., 2014) (American, non-virulent).

Based on our annotation approach, a total of 19,096 genes were identified in the TcI Sylvio X10/1 haploid genome sequence. The public CL Brener genome assembly has 11,106 annotated genes in one haplotype, 10, 596 in the other, and 3,397 in smaller contigs. We have previously estimated the total gene content of CL Brener, based on read coverage, to approximately 22,570 for the haploid genome (Arner et al., 2007). This is mostly due to the larger size of the multigene family clusters in the TcVI hybrid genome. The genome sequence was longer and less fragmented than the version generated previously using short-read sequencing of the same strain (Franzén et al., 2011), which indicated resolution of additional regions of the genome. About 24.1% (n = 4,602) of the total annotated genes were truncated, mostly due to the introduction of premature stop codons, and 67% of these were located within surface molecule gene arrays, sharing motifs of the complete genes.

The new assembly allowed an improved analysis of the *T. cruzi* surface molecule gene repertoire. While the regions can be described as large gene arrays that contain genes from different surface molecule gene families, the genes of each of the three major surface molecules families were mostly organized as multiple smaller groups or tandem arrays within the larger regions. After genome annotation, the total number of such smaller arrays were: trans-sialidases, 312, with 2,048 complete gene copies and 201 pseudogenes; mucins 98, with 2,466 complete copies and 111 pseudogenes; MASPs 264, with 1,888 complete copies and 245 pseudogenes. These three surface molecule gene families comprised 16.02 Mbp (39.04%) of the TcI Sylvio X10/1 genome and presented a high level of sequence diversity (**Figure 1A**). Sequence strand switches often delimited the surface molecule tandem arrays. Commonly, these arrays had two to four complete copies immediately followed by two or more truncated copies with motifs similar to the complete gene. The intergenic spaces between arrays were rich in simple and low complexity repeats with no identifiable regulatory elements. The VIPER and L1Tc retrotransposon elements, in clusters of two to four copies, were found in the proximity of, or inside, tandem arrays containing trans-sialidases, mucins and MASP genes. As the surface molecule genes are known to evolve rapidly and be highly variable (Andersson, 2011), the enrichment of VIPER and L1Tc elements in these regions supports the hypothesis that they may be involved in generating new surface molecule gene variants *via* recombination mediated by sequence homology.

Both Ser/Thr kinases and DEAD-box RNA helicase genes were found at both extremes of 34 (10.81%) trans-sialidase arrays located in chromosomes 1, 2, and 8. Searches against the RFAM database identified 1,618 small RNAs in the TcI Sylvio X10/1 genome. These were mostly ribosomal RNAs with the 5S rDNA subunit being the most common (31.9%) followed by ACA Box snoRNAs (30.9%), SSU rDNA (12.2%) and LSU rDNA (10.2%) subunits. We also found hits to telomerase RNA component (TERC), Catabolite Repression Control sequester (CrcZ), Protozoa Signal Recognition Particle RNAs, spliceosomal RNA subunits and miRNAs. The putative miRNAs identified in Sylvio X10/1 belong to the MIR2118 and MIR1023 families, previously not found in protozoan parasites. The functional relevance of these predicted small RNAs will need to be further validated *in vitro*. The miRNA segments were located in both strands within 1 Kb of genes coding for DEAD-box RNA helicases surrounding surface molecule gene tandem arrays.

### Genomic Variation Within The *Trypanosoma cruzi* TcI Clade

Intra-TcI genomic diversity was examined among 34 samples from six countries: United States, Mexico, Panama, Colombia, Venezuela and Ecuador, derived from a range of triatomine vectors and human patients of different clinical stages (**Table 2** and **Supplementary Table 1**). Our hybrid variant calling strategy, combined with removal of repeat elements and repeated genes, allowed us to identify genomic variants in the core and multigene family clusters in a reliable fashion (See methods).

**Table 2:** Genomic variants identified among the *Trypanosoma cruzi* TcI isolates.

GROUP	SNPs	INDELs	DELETION	DUPLICATION	TRANSLOCATION
Colombia*	158565	59520	439	1231	4140
Colombiana**	105023	30697	23	86	273
Venezuela	77232	70086	43	183	614
Ecuador	122122	84201	40	164	354
Panama	620499	238833	225	605	2060
Texas	101771	78499	69	303	978

*FcHc and CG clones from Colombia.

**TcI Colombiana strain.

A total of 1,031,785 SNPs and 279,772 INDELs shorter than 50 bp were called for all sequenced TcI isolates relative to the Sylvio X10/1 genome. INDELs presented an average density of 5.3 variants per Kb and SNPs 24.1 variants per Kb. An individual *T. cruzi* TcI isolate was found to contain an average of 61,000 SNPs and 6,820 INDELs with a density of 31.8 variants per Kb. However, these measures fluctuated depending on the geographical and biological source of the sample. Core regions had an average SNP density of 0.4 variants per Kb, in contrast with surface molecule multigene family clusters where approx. 10 variants per Kb were found. It was not surprising that the bulk of the genomic variants were located in the multigenic family clusters regions in all the isolates, with fewer differences in the core regions. Although several studies using single gene markers have identified heterogeneity in the TcI clade (Llewellyn et al., 2009; Guhl and Ramírez, 2011), the extent of this variation had not previously been assessed genome-wide.

The majority of INDELs (96%) were found in intergenic or noncoding regions, and 81% of these were located in surface molecule multigene family regions. INDELs within coding sequences were exclusively found to cause frameshifts turning the affected coding sequence into a pseudogene. This distribution of INDELs is a genomic signature that has been associated with non-allelic homologous recombination due to unequal crossing over (Parks et al., 2015) or microhomology-mediated end joining (Sfeir and Symington, 2015; Weckselblatt et al., 2015) (**Table 3**). However, these repair mechanisms, or something similar, have not yet been shown to be present in *T. cruzi*. Short insertions were more prevalent than short deletions, a pattern common to all the analyzed TcI genomes when compared to Sylvio X10/1. In the subtelomeric regions, short insertions (1–3 bp) occurred within the upstream and downstream portions of the coding sequences and usually involved the addition of one or more cytosines or guanines. Single-base pair deletions of an adenine or thymine were also observed within these regions, but at a lower frequency. Longer deletions (5–20 bp) and insertions (8–10 bp) were observed within trans-sialidases, Retrotransposon Hot Spot (RHS), pseudogenes and, at a lower frequency, L1Tc retroelements.

**Table 3:** Patterns of INDELs and their associated mechanisms of origin.

INDEL type	Example	Mechanism	Frequency*
HR - deletion	GCATAAA*aa*AAAGC	NAHR	756 411
HR - insertion	CACA*AAAAAAAAAAA*GCTAC	NAHR	521 002
TR - mixed	ACACAC*acac*ACACAC*AC*AC	NAHR	118 432
Non-repetitive	TAGCACagtGACTTCAC*AGC*CTG	NHEJ-like	28 389
Long Insertion	C*GGCTAGACCAGGTACAGTC*A	MMEJ	32 666
Long Deletion	GC*acactgacacgacactgacacactgaa*A	MMEJ	31 712

HR, Homopolymer run; TR, Tandem Repeat.

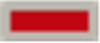
 = Deletion

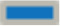
 = Insertion

*For all the 34 TcI genomes compared against Sylvio X10/1.

### Population Genomics of the *Trypanosoma cruzi* TcI Clade

**Figure 2:**
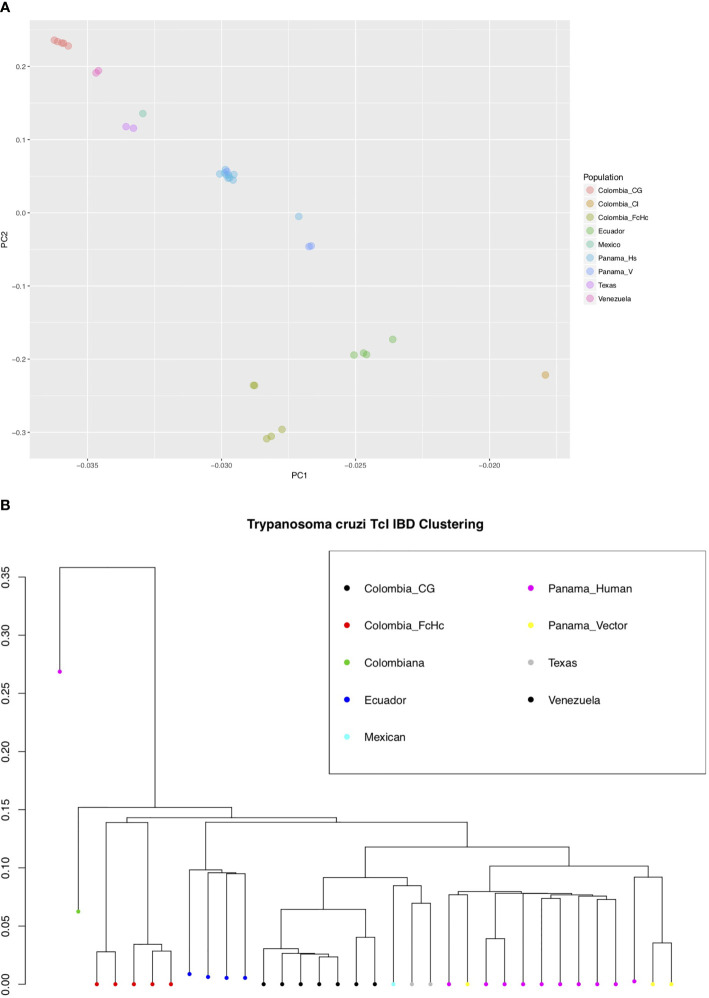
**(A)** Bayesian principal component analysis (PCA) of *T. cruzi* TcI strains using INDELs. The percentage of variance for PC1 was 46.2 and for PC2 28.6. **(B)** Identity by Descent (IBD) dendogram of *T. cruzi* TcI strains using SNPs, calculated using 1000 bootstraps. Both analyses, using different markers, support the population structure of the analyzed TcI samples. Notably, the highly virulent TcI Colombiana and the Panamanian TcI H1 from a chronic patient are presented as outliers [**(B)**, far left].

We used the short genomic variants to analyze the population genomics of the *T. cruzi* TcI clade, and where possible taking into account the different sample sources (insect vector or human host), clinical outcome of the infected patients and geographic locations (**Supplementary Table 1**). This sampling strategy allowed comparison of parasite population structure in different environments. Interestingly, a Bayesian PCA analysis using INDELs and IBD-based hierarchical clustering using only SNPs from core regions for all the samples showed a mostly geography-specific population structure (**Figures 2A, B**).

**Figure 3:**
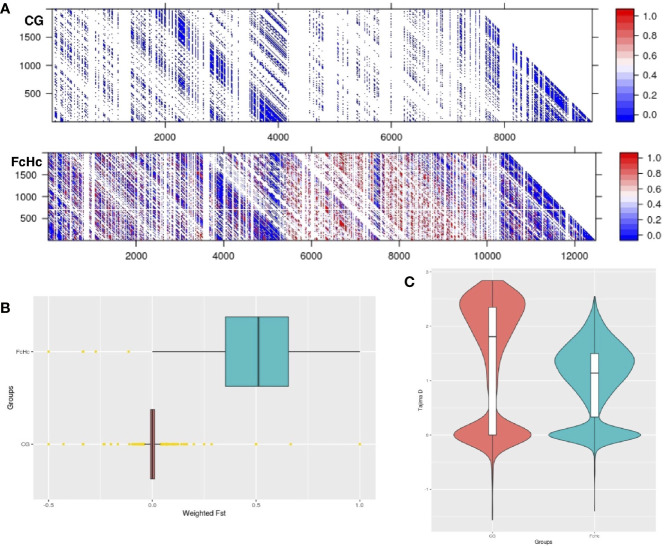
**(A)** Linkage disequilibrium matrix (r2) of chromosome 2 for the Colombian CG and FcHc clones. LD values range from 0 (recombination) to 1 (no recombination). **(B)** Genome-wide *Fst* distribution in 10 Kb bins displaying a state of panmixia for the CG clones and moderate genetic differentiation in the FcHc clones, yellow dots represent outlier bins. The differences in chromosome length are caused by missing sequences in these strains, that resulted in regions with no mapping that were removed from the analysis. **(C)** Distribution of subtelomeric *Tajima’s D* selection test in both groups displaying overall balancing selection (D > 0) in these regions for both clones.

The analysis of the variation between two Colombian TcI isolates made it possible to compare parasites from a HIV-positive patient with fatal cardiomyopathy (CG) and from an acute chagasic patient infected by oral transmission (FcHc). To increase accuracy, repeat elements, repeated genes and repeated surface molecule family genes were excluded from this analysis, while core regions and non-repeated, unique surface molecule genes and other genes were kept, in order to have markers outside the core regions. The latter were selected by lower sequence similarity to known surface molecule genes. A total of 158,565 well-supported SNPs, called by both GATK and FreeBayes, was selected from these clones, and used to calculate global and per-site population genetic statistics. These samples displayed distinctive behavior in a global analysis of genomic diversity by separating into two well-defined clusters, as can be seen in **Figures 2A, B**. Linkage Disequilibrium (LD) analyses were performed genome-wide for both groups using the r2 statistic; revealing a fluctuating pattern of LD across the entire genome with large blocks of low r2 values—implying a recombinatorial process—present at distinctive chromosomal locations that were specific to each group of clones. Particularly, CG clones had less genetic diversity than FcHc clones (**Figure 3A**) and displayed a trend toward LD, whereas FcHc clones presented a more dynamic LD pattern. Values of r2 near zero were more common in LD sliding windows containing genes coding for surface molecules and r2 values closer to one were present exclusively in core regions rich in housekeeping genes, indicating that these regions are more stable. For the CG and FcHc clones we calculated a global Fixation index (*Fst*) value of -0.9377958 and -0.1162212, respectively (**Figure 3B**). These values are consistent with genetic differentiation in recombination hotspots in the multigene family regions. The global Tajima’s D value for the CG clones was 1.373 and 0.9906 for FcHc clones, suggesting the presence of multiple alleles at variable frequencies in both populations (**Figure 3C**). This pattern was more evident in the multigene family regions, which is consistent with balancing selection of surface molecules. The values for each set of clones were calclulated separately, from different sets of SNPs, which makes a direct comparison in smaller regions difficult. We were only able to detect the larger patterns described above.

Analyses of genomic variation between samples isolated from humans and vectors from Mexico, Panama and Ecuador revealed that the global genetic differentiation among samples isolated from vectors was *Fst* = 0.1289547 whereas for samples isolated from humans the observed was *Fst* = -0.05521983. The patterns of linkage disequilibrium between human and vector derived isolates were similar to those observed in the Colombian clones. Estimates of the Tajima’s D statistic revealed a distinctive pattern of selection between the two groups. Balancing selection was detected specifically in regions containing tandem gene arrays coding for surface molecules in all the samples derived from vectors, regardless of their geographical origin; whereas selective sweeps were present in the same regions in human-derived samples. Large genomic areas (> 50 Kb) containing surface molecule genes displayed negative Tajima’s D values in human-derived isolates, in contrast with the pattern observed in vector-derived isolates with long genomic stretches (> 70 Kb) of positive Tajima’s D values and short genomic blocks (< 5 Kb) with negative values. We speculate that these patterns may be caused by selection pressure from the immune system in human-derived strains, which is absent in strains that have grown in insects for extended times.

### Genome Structural Variation

Genomic structural variants, such as deletions, tandem and interspersed duplications, genomic inversions and chromosomal break-ends, were observed ubiquitously throughout the genomes of the analyzed TcI strains. The most common type of intrachromosomal structural variant observed was tandem duplications followed by deletions larger than 50 Kb (**Table 2**). Chromosomal break-ends, similar to the unbalanced chromosomal translocations observed in many eumetazoans, were the most abundant type of structural rearrangement and they were only present in surface molecule multigene family regions that were statistically enriched with retroelements and simple repeats. The recombination breakpoints were found to occur at simple repeats and retrotransposons of the VIPER and L1Tc class.

The detected recombination events were found to involve fragments ranging between 20–150 Kb in length and in most cases contained fragments or even complete coding sequences for surface molecule genes, such as trans-sialidases, mucins and MASP genes and other surface glycoproteins (gp63/gp85). Housekeeping genes did not appear to have been affected by these genomic rearrangements. We detected several instances where rearrangements resulted in altered or longer coding sequences by superimposing fragments—or the entire coding sequence—on genes of the same family located in a different genomic location. This was found to have occurred both in the Colombian and the Texas isolates by recombination between trans-sialidases from different chromosomes. The biological relevance of the new altered gene sequences is not known.

Retroelements could be found within or near genomic regions containing surface molecule gene tandem arrays and L1Tc fragments or their entire sequence were also found near all the observed rearrangements, where they were inserted into regions containing simple repeats composed by AT dimers. Data on the genomic positions for repeat elements have been listed in **Supplementary Tables 3–5**.

Multiple such examples of the generation of possible new surface molecule gene variants were identified in TcI. It is a possibility that the parasite uses specific molecular mechanisms of recombination that can rapidly generate surface molecule diversity, allowing it to increase the genomic plasticity required to adapt to changing environments and evade immune responses during short and long-term infections in various host species.

The sizes of the tandem duplications ranged from 6 to 75 Kb and mainly involved tandem arrays coding for surface molecules, mostly trans-sialidases and mucins, but also Dispersed Gene Family 1 (DGF-1) and several hypothetical proteins. The breakpoints of these duplications were surrounded by simple repeats and retroelements in multigene family regions. A tandem duplication event could involve between four and 25 copies of a specific gene when in the surface multigene family regions, whereas in core regions the number was between two and eight. We observed that large deletions occurring in multigene family regions were surrounded by simple repeats of the type (T)n and (AT)n and retrotransposons of the L1Tc class, containing surface molecule gene tandem arrays. Deletions in these genomic regions tended to be shorter (4–12 Kb) and sample-specific.

### Distribution of Copy Number Variation (CNV) Within the TcI clade

CNV varied extensively between *T. cruzi* TcI strains. There have been previous attempts to assess CNV in the *T. cruzi* genome (Minning et al., 2011), but these studies were performed using DNA tiling microarrays with probes designed using the TcVI CL Brener strain assembly, in which multigenefamily regions are more difficult to study.

**Figure 4:**
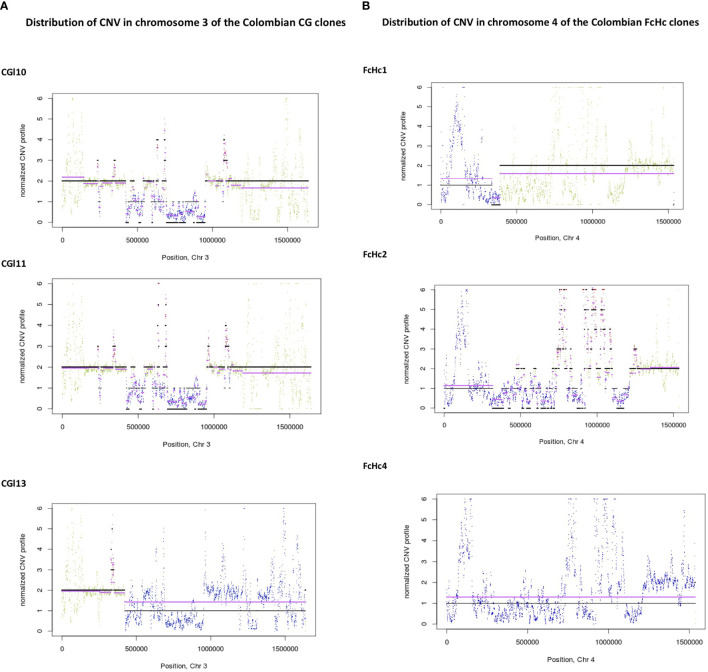
Distribution of CNV changes in chromosome 3 of the Colombian **(A)** CG clones and **(B)** FcHc clones. Black lines represent the reference genome sequence and cyan lines represent the sample under study. Each sliding window for CNV evaluation is represented as a dot. A drastic change in CNV can be noted in the FcHc clones whereas the CG clones seem to be less affected.

The distribution of CNV in the genomes of the studied TcI samples involved segments of an average size of 5 Kb. We observed blocks of segmental CNV within a chromosome with a pattern that was unique to each sample. Notably, the Colombian clones presented individual profiles of CNV (**Figures 4A, B** and **Supplementary Figures 3–8**) despite being derived from the same clinical isolates.

Sequence blocks affected by segmental CNVs contained retrotransposons of the VIPER and L1Tc class, as well as surface molecule genes surrounded by simple repeats. The isolate-specific nature of these CNV events demonstrates the high level of within-clade diversity of the TcI samples. The distribution of CNV across the *T. cruzi* genome reinforces the dynamic nature of the multigene family clusters and the surface molecule gene families.

## Discussion

Complete reconstruction of the *T. cruzi* genome to encompass the subtelomeric regions and surface molecule multigene family clusters proved to be difficult to achieve using short reads, due to sequencing library preparation biases and a genome architecture that is rich in long stretches of simple repeats, large repetitive gene families and multiple retrotransposons. In 2016, we used long PacBio sequencing reads to provide the most complete genome sequence of a *T. cruzi* strain to date and this reference genome was made public through Genbank and TriTrypDB. This allowed us to perform a detailed analysis of the repertoire of complex genes families that encode cell surface molecules, considered to be involved in cell invasion and evasion of the host immune response. We could clearly see the duality in the organisation of the parasite genome, comprised of a core genomic component with few repetitive elements and a slow evolutionary rate, resembling that of other related protozoa, and contrasting, highly plastic multigene family clusters encoding fast-evolving surface antigens, with abundant interspersed retrotransposons. The structural changes that generate and maintain diversity in *T. cruzi* surface molecules have certain mechanistic parallels in other protozoa such as those recently described in *Plasmodium falciparum* (Miles et al., 2016), but differing from the shorter, less repetitive genome of the non-virulent, human-infective *Trypanosoma rangeli*.

In order to overcome the limitations of short read mapping to a highly complex genome such as *T. cruzi*, we first mapped the reads against the repeat masked genome using `bwa-mem` with probabilistic read placement and multi-mapping probability assignment. The mapping results were subsequently submitted for statistical evaluation using Stampy (https://genome.cshlp.org/content/21/6/936). The Stampy algorithm assigns a probability for read-base misplacement and repeats. To evaluate the results we used two different variant callers (GATK + Freebayes) which also take into account these scores. For a variant to be considered, it needed to be reported by both variant calling methods and pass the filters. This strategy and the TcI reference genome made it possible to carry out the Tc1 population genomics study.

Early studies of the genetic diversity of *T. cruzi* using geographically disparate sampling and restricted comparisons of genetic diversity suggested a clonal population structure (Tibayrenc et al., 1990; Tibayrenc and Ayala, 1991); however, population genetics with an expanded set of markers have now challenged this view (Gaunt et al., 2003; Westenberger et al., 2005; Llewellyn et al., 2009). Nevertheless, there are still conflicting views as to which model best describes the population structure of *T. cruzi* (Messenger and Miles, 2015; Tibayrenc and Ayala, 2015). The newer Sylvio X10/1 genome sequence has enabled extensive genome-wide comparative population genomics analyses, which may shed light on this issue (Schwabl et al., 2019; Rose et al., 2020). Our comparative analyses of 34 *T. cruzi* isolates and clones from the TcI clade suggested many recombination events and population indices normally associated with genetic exchange between strains, which are more likely to be caused by the extensive repeat-driven recombination in the subtelomeric regions. The extent of variation in the multigene family clusters rich in surface antigen coding genes and the geographical clustering of strains based on geographic distribution indicates active, on-going adaptation to host and vectors. This need for phenotypic—and thus genomic—versatility may impel the active generation of sequence diversity in *T. cruzi*. Further analyses of the evolution of multigene family clusters will yield much more detailed understanding of diversity within and between the six currently recognised genetic lineages of *T. cruzi* (Andersson, 2011). We have shown how the genome architecture and dynamic multigene family clusters of *T. cruzi* may provide a mechanism to rapidly generate sequence diversity, required to escape the host immune response and adapt in response to new environments. It is the striking richness in simple repeat, retrotransposons and motif conservation in the multigene family clusters that renders these genomic areas susceptible to structural change, similar to yeast and other pathogens (Aksenova et al., 2013; de Jonge et al., 2013; Faino et al., 2016; Weatherly et al., 2016). Retrotransposons have been associated with the generation of complexity in genomic regions in mammals and plants and with control of gene expression (de Jonge et al., 2013; McConnell et al., 2013). In the case of *T. cruzi*, they appear to generate novel variants *via* mechanisms that exploit sequence homology. The presence of the simple repeats and retrotransposons near surface molecule coding genes provides the microhomology for both mechanisms to operate in such regions. Besides retrotransposons, the modular structure of the multigene families MASP and Trans-sialidase, where different genes share conserved motifs, could also provide microhomology needed for this homologous recombination (El-Sayed et al., 2005; Weatherly et al., 2016). Our analysis of INDELs and chromosomal breakpoints in the subtelomeric regions confirmed that a mechanism similar to NAHR or MMEJ operates as source of sequence diversity, for example by transposition of trans-sialidase genes or pseudogenes to produce new sequence mosaics. The required recombination machinery is conserved in *T. cruzi* (Ramesh et al., 2005). Furthermore, these mechanisms would explain the higher amount of pseudogenes observed in the surface molecule regions.

Retrotransposons were first reported from *T. cruzi* in 1991 (Villanueva et al., 1991). The presence of these elements may also partly account for the previously reported widespread observation of copy number variation in different *T. cruzi* strains (Minning et al., 2011). Thus, we find that repeats near the surface molecule genes appear to drive recombination in *T. cruzi*. The apparent inability of *T. cruzi* to condense chromatin may facilitate transposition in a stochastic fashion, facilitating generation of sequence diversity in exposed regions of the genome. A similar process has been described in the neurons of mammals and insects (Erwin et al., 2014) but not in any other unicellular organism. Retrotransposons may also have an important role as gene transcription regulators: they may either silence or promote gene expression, due to their susceptibility to DNA methylation or by providing potential binding sites respectively, as observed previously (Elbarbary et al., 2016). This lack of a well-defined transcriptional regulation machinery in the *T. cruzi* genome may suggest a link to the requirement for retrotransposon closely associated with gene tandem arrays.

## Conclusion

Here we describe the sequencing and assembly of the complete genome of the *Trypanosoma cruzi* TcI strain Sylvio X10/1, which was made public in 2016. This genome sequence enabled the first resolution of the complex multiple gene families that encode *T. cruzi* surface molecules, and provided a basis for *T. cruzi* population genomics. We discovered an extraordinary concentration of retrotransposons among the multigene family clusters and indications of repeat-driven recombination and generation of antigenic diversity, providing the mechanisms for *T. cruzi* to evade the host immune response, and to facilitate the adaption to new host and vectors. This genome will provide an invaluable resource to facilitate the prospective discovery of novel drug targets and vaccine candidates for Chagas disease.

## Data Availability Statement

The datasets presented in this study can be found in online repositories. The names of the repository/repositories and accession number(s) can be found below: https://www.ncbi.nlm.nih.gov/genbank/, ADWP00000000 and https://www.ncbi.nlm.nih.gov/genbank/, SRP076682.

## Author Contributions

CT-L and BA conceived and designed the study. CT-L designed and executed computational analyses. MY prepared Sylvio X10/1 genomic DNA for PacBio sequencing and performed manual annotation of surface molecule genes. JEC, AS, JR, FG, SO-M, JAC, ST, HC, RG, KJ, MB, PH, KM, MJG, and BB provided genomic DNA for TcI isolates. JR-C and DB created chromosome maps for surface molecules. MM, LM, ML, JR-C, GM, and EG contributed to the interpretation of the results. CT-L, EG, MM, and BA wrote the manuscript. All authors contributed to the article and approved the submitted version.

## Funding

This research was funded by grants from the Knut and Alice Wallenberg Foundation, The Swedish Research Council and the European FP7 program, Contract No. 223034. LM was funded by a grant from the NIH (5R01AI107028); DB, EG, and ST were funded by grants from CNPq (Brazilian Government Agency). GM was a recipient of a CAPES/PRINT Program Scholarship.

## Conflict of Interest

The authors declare that the research was conducted in the absence of any commercial or financial relationships that could be construed as a potential conflict of interest.

## Supplementary Material

Supplementary Table 2 A list of coordinates for the synteny analysis comparing the Sylvio X10/1 genome with CL Brener for all chromosomes.

Supplementary Table 3–5 The tables list the genomic coordinates and identity of repeat elements of various types that have been identified in the Sylvio X10/1 genome sequence. The lists have been used for the annotation of the publically available genome sequence.

Supplementary Figure 1 Results from mapping Illumina reads back to the completed genome sequence. The graphs show the overall coverage across the genome.

Supplementary Figure 2 Results from mapping Illumina reads back to the completed genome sequence. The plots show coverage across each chromosome. Local variation in coverage indicate the presence of highly repeated regions.

Supplementary Figure 3–8 Distribution of CNV changes in the Colombian CG clones and FcHc clones for all chromosomes.
