# Supplementary material for: Repeat-Driven Generation of Antigenic Diversity in a Major Human Pathogen, Trypanosoma cruzi
Source: Front Cell Infect Microbiol. 2021 Mar 3;11:614665. doi: 10.3389/fcimb.2021.614665 (PMC7966520; doi:10.3389/fcimb.2021.614665)
Supplement: Supplementary file 1 [file DataSheet_1.pdf]

Supplementary Table 1: *Trypanosoma cruzi* strains used in this study.

Strain	Country	Source	Clinical form	Sequencing coverage*
H1a	Panama	Human	Chronic	31 X
H2	Panama	Human	Asymptomatic	28 X
H3	Panama	Human	Asymptomatic	32 X
H4	Panama	Human	No Disease	29 X
H5	Panama	Human	Chronic	31 X
H6	Panama	Human	Asymptomatic	31 X
H7	Panama	Human	Asymptomatic	28 X
H8	Panama	Human	No Disease	32 X
H12	Panama	Human	No Disease	31 X
H14	Panama	Human	Chronic	32 X
H15	Panama	Human	Chronic	31 X
V1	Panama	*P. geniculatus*	ND	28 X
V2	Panama	*R. pallescens*	ND	25 X
V3	Panama	*T. dimidiata*	ND	28 X
TBM3324	Ecuador	*R. ecuadoriensis*	ND	28 X
TBM3406B1	Ecuador	*R. ecuadoriensis*	ND	25 X
TBM3479B1	Ecuador	*R. ecuadoriensis*	ND	29 X
TBM3519W1	Ecuador	*R. ecuadoriensis*	ND	28 X
X10462-P1C9	Venezuela	Human	ND	31 X
X12422-P1C3	Venezuela	Human	ND	29 X
Colombiana	Colombia	Human	ND	30 X
CGI10	Colombia	Human	Acute Co-infection	30 X
CGI11	Colombia	Human	Acute Co-infection	30 X
CGI12	Colombia	Human	Acute Co-infection	30 X
CGI13	Colombia	Human	Acute Co-infection	30 X
CGI14	Colombia	Human	Acute Co-infection	30 X

CGI15	Colombia	Human	Acute Co-infection	30 X
FcHcl1	Colombia	Human	Acute Oral	30 X
FcHcl2	Colombia	Human	Acute Oral	30 X
FcHcl3	Colombia	Human	Acute Oral	30 X
FcHcl4	Colombia	Human	Acute Oral	30 X
FcHcl5	Colombia	Human	Acute Oral	30 X
H1b	Mexico	Human	Acute	32 X
TD23	Texas	*T. dimidiata*	ND	30 X
TD25	Texas	*T. dimidiata*	ND	30 X

NA = Not determined.

* Illumina data
