# Supplementary material for: Repeat-Driven Generation of Antigenic Diversity in a Major Human Pathogen, Trypanosoma cruzi
Source: Front Cell Infect Microbiol. 2021 Mar 3;11:614665. doi: 10.3389/fcimb.2021.614665 (PMC7966520; doi:10.3389/fcimb.2021.614665)
Supplement: Supplementary Table 2 — A list of coordinates for the synteny analysis comparing the Sylvio X10/1 genome with CL Brener for all chromosomes. [file DataSheet_2.pdf]

Supplementary Table 2.

TcI chromosome	Start	End	TcVI chromosome	Start	End
1	7856	77686	36	929579	450980
1	83489	88732	36	926768	924310
1	133750	167254	36	852285	886001
1	273687	494393	36	612805	744997
1	450605	536813	36	472531	550117
1	558773	585115	36	955061	954896
1	745948	801318	36	452202	408279
1	858072	950310	36	297087	387808
1	951485	966822	36	294640	281513
1	1017124	1090220	36	165729	226185
1	1132170	1195287	36	71875	123492
1	1219022	1226231	36	28750	36118
1	1387897	1399200	37	608730	599838
1	1588345	1620727	37	719134	723564
1	1630647	1641246	37	742449	733498
1	1654666	1834100	37	756633	793427
1	1962391	1980284	37	958682	954960
1	2002380	2073379	37	990728	1019194
1	2120689	2134495	37	1101714	1089576
1	2266301	2486930	37	1288260	1254200
1	2457588	2470650	37	1272816	1275507
1	2705936	2741331	21	509184	492638
1	2764952	3057081	21	151987	451049
1	3077230	3082308	21	100516	105611
2	24587	406636	40	1051196	813126
2	231884	365128	40	901785	965191
2	455356	467278	40	1118606	1114534
2	515174	567806	28	682218	341363
2	748858	787291	40	1758882	1795536
2	811459	855888	40	1860430	1829147
2	874231	878022	40	1882685	1882080
2	910082	953323	40	1973257	1931928
3	150274	311269	29	673901	828309
3	237698	239549	29	743531	747694
3	368762	582555	29	614857	472673
3	417337	683787	29	5663	136301
3	968897	1064333	11	165441	193031
3	1109612	1239660	11	286135	390576
3	1329429	1335991	11	412124	419434
3	1395258	1401140	11	479862	485767
4	11328	70106	31	400527	402233
4	78689	116525	31	388935	356811
4	84626	90244	31	362593	368310
4	278770	300602	31	425887	428888
4	449536	525299	31	416246	442013
4	1073464	1082600	30	46352	56129
4	1157507	1375702	30	231107	71481
4	1494015	1508440	30	327937	339413
5	168032	247858	34	636401	557228
5	375510	392135	34	478981	469467
5	477005	496974	34	374885	356749
5	504483	630637	34	275830	338704
5	584231	586021	34	261540	261142

5	708666	735766	34	136393	110812
5	881049	953130	26	459194	520334
5	1004744	1107077	26	629660	672620
5	1090998	1157855	26	723339	662921
5	1212950	1287111	22	289290	393019
6	37006	248369	3	151803	100844
6	776794	870058	31	456164	295434
6	962796	994842	31	169207	165383
6	1129070	1160194	25	767102	408329
7	167876	328998	2	71990	4855
7	595262	607031	9	457347	469319
7	948185	953722	7	154563	160232
7	963317	1157108	7	192261	261309
7	1440907	1617960	38	552180	762060
7	1512505	1656726	29	200001	375357
7	1697201	1711418	18	297739	340376
7	1720456	1775850	38	726777	897652
7	1791743	1879080	41	2076276	2098145
8	212362	222416	34	670906	290876
8	270925	292231	41	540003	520288
8	757598	894741	4	1580	140382
9	132943	253709	27	120105	179273
9	265682	336709	27	214056	208092
9	441812	667575	27	451676	321428
9	778836	819555	27	611131	572655
9	924249	992063	27	782104	442770
9	1019045	1050287	27	822539	828797
10	521024	522732	39	342553	341783
10	693760	731592	39	411584	432058
10	932197	972406	39	545842	507602
10	1015976	1032451	39	590001	606683
11	206347	308978	23	464864	540015
11	302266	642669	23	398754	427159
11	596278	737402	23	127993	285278
11	800414	817093	23	30248	25891
11	851247	1009042	35	459205	572850
11	900817	904654	35	522354	526242
11	916942	921753	35	538682	543479
11	1017217	1020599	31	648854	765769
11	1030776	1043034	35	620597	614294
12	138083	155473	14	514882	532288
12	286266	329704	14	370376	401923
12	417309	562302	40	166126	1732611
12	539409	552519	14	320717	333817
12	938031	1014502	14	83019	107491
13	278972	298184	28	35950	14587
13	412025	509703	12	123806	181858
13	540150	781065	12	366705	440546
13	624819	756622	12	368712	282329
13	787148	883835	12	461179	449570
14	525496	561759	24	471323	673169
14	806378	911887	26	102142	196621
15	114987	228201	33	700523	619274
15	133787	244189	33	660742	586846

15	265381	277196	33	546091	558009
15	394640	512341	37	498269	416753
15	453188	593585	37	334353	453516
15	613173	615696	37	311833	314550
15	620342	744999	37	270995	284839
15	752073	843026	37	210818	187959
15	853544	929839	37	124702	116133
16	182974	216099	33	134968	107792
16	211716	355944	33	131729	197716
16	385778	443378	33	460303	526425
16	446232	882525	32	167145	548677
16	871229	1045104	9	94085	208835
16	1142767	1194348	35	325939	280327
16	1150201	1456912	35	418504	143405
16	1274500	1313885	35	195045	172318
16	1383492	1427962	35	97700	387801
18	16564	109640	41	2314639	2222571
18	296681	317572	20	40718	34043
18	337689	454007	20	64221	62505
18	407741	415309	20	128227	135821
18	475608	524402	20	229600	201482
18	549119	566830	20	270794	288649
18	601270	696006	20	366089	532553
18	648736	654622	20	352695	348422
18	720384	733176	20	435711	424502
19	249779	264539	39	1817821	1819365
19	280118	304806	39	1774251	1783214
19	378049	395762	39	1679330	1697527
19	418886	430228	39	1659357	1648769
19	447115	720370	39	1630774	1364386
19	701169	739121	39	1381823	1343283
19	739159	776802	39	1337975	1309235
20	26583	184770	16	376916	365088
20	316309	347892	16	538580	521748
20	451945	484407	39	1032275	1288402
20	481924	517113	38	804104	339772
20	485435	532471	16	476408	460428
20	549962	555156	16	409318	414690
20	560887	564962	16	420390	425192
21	50378	52010	26	226619	228336
21	158996	174747	40	118135	134214
21	207272	290184	40	238944	168204
21	322439	696749	40	633398	283435
21	350539	408136	40	367209	311300
22	34303	70754	41	1576114	1050401
22	503823	609479	41	1806623	974933
23	500144	534712	38	235419	200863
23	539689	543963	38	193759	191606
23	564632	672687	38	55314	107492
23	603557	651448	38	79246	125130
24	57258	61659	41	1590720	1135481
24	92474	117966	38	1145835	603942
24	142984	168000	41	870805	875998
24	198339	365874	41	849029	568465

25	49900	56040	39	838177	844105
26	35536	118173	32	454286	473823
26	138951	241566	32	558693	627874
26	253028	280471	32	676177	690927
26	285969	306199	32	723912	723569
26	334962	420984	32	758725	846138
26	342791	349141	32	764921	771487
26	399338	429210	32	820380	853154
27	29202	31988	35	768696	771526
27	85263	379733	35	848356	1074337
27	386785	414388	35	1083248	1085368
28	280381	284143	15	390049	393870
28	289944	335706	15	383171	373573
28	346002	512546	15	121446	341253
28	411420	576889	15	269181	23758
29	74121	285558	8	151616	181606
29	204073	292364	8	334568	238565
30	31580	51972	41	1614684	1086746
30	415099	494433	41	288998	297612
31	9716	62944	41	119463	159972
31	332342	370945	33	873160	842769
31	392913	418156	33	888226	950619
31	430800	442394	33	982667	972255
32	477807	826834	17	283640	604898
32	523385	548251	17	539354	564547
32	896023	932018	17	212711	190476
33	49493	98307	38	947735	947466
33	313139	411647	28	161086	63881
34	373479	405122	21	161131	179455
34	443634	448683	25	658949	663188
35	244386	268575	32	153282	130006
35	275620	296821	32	180941	161796
35	340016	348259	32	221002	229607
35	397152	436735	32	269817	272179
35	520752	525378	32	260562	257305
36	95766	174434	5	172281	119417
36	203387	269661	5	52942	44606
36	341598	501394	10	81256	234890
37	128966	160029	22	213902	286257
37	176059	253406	22	310188	378249
37	291498	327700	22	446340	436210
37	366314	393259	22	487798	518033
37	409440	423762	22	547508	534773
38	90363	439040	6	331705	200376
38	197076	379081	6	214427	210653
39	8358	165147	30	523731	382599
39	123286	135252	30	482114	494109
39	232544	245196	30	582047	589056
40	29310	426004	27	508175	495024
41	154725	246127	25	166168	84892
41	225981	307744	25	226581	150244
41	295566	358697	25	235900	218404
42	35715	611185	24	197730	351772
42	750268	792639	1	42755	28807

43	27839	70867	34	963430	975936
43	80967	386137	34	786390	932980
43	325095	417297	34	779940	783035
44	92955	290340	13	297981	472842
44	416696	423156	13	153107	159843
45	82032	87932	19	541212	539272
45	121216	263207	19	508001	435564
46	233500	261791	38	1290994	1263902
46	319686	339832	8	352018	346408
