# Supplementary material for: Repeat-Driven Generation of Antigenic Diversity in a Major Human Pathogen, Trypanosoma cruzi
Source: Front Cell Infect Microbiol. 2021 Mar 3;11:614665. doi: 10.3389/fcimb.2021.614665 (PMC7966520; doi:10.3389/fcimb.2021.614665)
Supplement: Supplementary Table 3–5 — The tables list the genomic coordinates and identity of repeat elements of various types that have been identified in the Sylvio X10/1 genome sequence. The lists have been used for the annotation of the publically available genome sequence. [file DataSheet_3.pdf]

Supplementary table 3.

Chromosome	startR	stopR	strand	repeat
1	45727	46301	+	Target "Motif:TcVIPER" 18 603
1	46380	46897	+	Target "Motif:TcVIPER" 807 1307
1	46913	49634	+	Target "Motif:TcVIPER" 1381 4182
1	49715	49779	+	Target "Motif:TcVIPER" 4322 4389
1	62471	63040	+	Target "Motif:TcVIPER" 18 601
1	63136	63640	+	Target "Motif:TcVIPER" 823 1307
1	63656	66383	+	Target "Motif:TcVIPER" 1381 4182
1	66464	66529	+	Target "Motif:TcVIPER" 4322 4389
1	70104	70201	+	Target "Motif:TcVIPER" 33 126
1	70194	70715	+	Target "Motif:TcVIPER" 67 601
1	70813	71321	+	Target "Motif:TcVIPER" 823 1307
1	71337	74064	+	Target "Motif:TcVIPER" 1381 4182
1	74145	74211	+	Target "Motif:TcVIPER" 4322 4389
1	96944	97408	+	Target "Motif:TcVIPER" 4169 4390
1	101122	105324	+	Target "Motif:TcVIPER" 1 4221
1	101122	105324	+	Target "Motif:TcVIPER" 1 4221
1	119280	121660	+	Target "Motif:TcVIPER" 1801 4182
1	538936	539433	-	Target "Motif:TcVIPER" 823 1307
1	539530	540105	-	Target "Motif:TcVIPER" 18 601
1	543285	543811	-	Target "Motif:TcVIPER" 1381 1947
1	543827	544332	-	Target "Motif:TcVIPER" 823 1307
1	544430	545000	-	Target "Motif:TcVIPER" 19 601
1	552644	556142	-	Target "Motif:TcVIPER" 666 4182
1	606842	609586	-	Target "Motif:TcVIPER" 1381 4182
1	609602	610109	-	Target "Motif:TcVIPER" 823 1307
1	610207	610780	-	Target "Motif:TcVIPER" 19 601
1	615851	616345	-	Target "Motif:TcVIPER" 1519 2006
1	616379	616436	-	Target "Motif:TcVIPER" 1381 1437
1	616452	616954	-	Target "Motif:TcVIPER" 823 1307
1	617052	617622	-	Target "Motif:TcVIPER" 16 601
1	625217	625287	-	Target "Motif:TcVIPER" 4320 4389
1	625362	628095	-	Target "Motif:TcVIPER" 1381 4182
1	628111	628617	-	Target "Motif:TcVIPER" 823 1307
1	628715	629279	-	Target "Motif:TcVIPER" 27 601
1	641837	641901	-	Target "Motif:TcVIPER" 4322 4389
1	641981	644685	-	Target "Motif:TcVIPER" 1381 4182
1	644701	645207	-	Target "Motif:TcVIPER" 823 1307
1	645306	645894	-	Target "Motif:TcVIPER" 1 601
1	650459	651044	-	Target "Motif:TcVIPER" 1 603
1	658629	661351	-	Target "Motif:TcVIPER" 1381 4182
1	661367	661870	-	Target "Motif:TcVIPER" 823 1307
1	661968	662546	-	Target "Motif:TcVIPER" 1 594
1	675118	677864	-	Target "Motif:TcVIPER" 1381 4182
1	675118	677864	-	Target "Motif:TcVIPER" 1381 4182
1	675118	677864	-	Target "Motif:TcVIPER" 1381 4182

1	675118	677864	-	Target "Motif:TcVIPER" 1381 4182
1	675118	677864	-	Target "Motif:TcVIPER" 1381 4182
1	675118	677864	-	Target "Motif:TcVIPER" 1381 4182
1	675118	677864	-	Target "Motif:TcVIPER" 1381 4182
1	675118	677864	-	Target "Motif:TcVIPER" 1381 4182
1	677880	678388	-	Target "Motif:TcVIPER" 823 1307
1	678486	679080	-	Target "Motif:TcVIPER" 1 601
1	686738	689392	-	Target "Motif:TcVIPER" 1519 4182
1	694289	697035	-	Target "Motif:TcVIPER" 1381 4182
1	697051	697559	-	Target "Motif:TcVIPER" 823 1307
1	697657	698231	-	Target "Motif:TcVIPER" 19 601
1	705804	705874	-	Target "Motif:TcVIPER" 4320 4389
1	705951	708660	-	Target "Motif:TcVIPER" 1381 4182
1	708676	708923	-	Target "Motif:TcVIPER" 1062 1307
1	712121	714829	-	Target "Motif:TcVIPER" 1381 4182
1	714845	714966	-	Target "Motif:TcVIPER" 1186 1307
1	859655	861865	-	Target "Motif:TcVIPER" 382 2644
1	859655	861865	-	Target "Motif:TcVIPER" 382 2644
1	859655	861865	-	Target "Motif:TcVIPER" 382 2644
1	859655	861865	-	Target "Motif:TcVIPER" 382 2644
1	994504	994781	+	Target "Motif:TcVIPER" 1555 1837
1	994775	996052	+	Target "Motif:TcVIPER" 3117 4390
1	1003469	1003741	+	Target "Motif:TcVIPER" 1558 1837
1	1003735	1004995	+	Target "Motif:TcVIPER" 3117 4375
1	1014256	1014694	+	Target "Motif:TcVIPER" 4167 4389
1	1020805	1021252	+	Target "Motif:TcVIPER" 4167 4390
1	1066841	1067292	+	Target "Motif:TcVIPER" 4167 4390
1	1246059	1250451	+	Target "Motif:TcVIPER" 1 4390
1	1246059	1250451	+	Target "Motif:TcVIPER" 1 4390
1	1263334	1264899	+	Target "Motif:TcVIPER" 1 1525
1	1263334	1264899	+	Target "Motif:TcVIPER" 1 1525
1	1264890	1267441	+	Target "Motif:TcVIPER" 471 4221
1	1281670	1282432	+	Target "Motif:TcVIPER" 12 782
1	1282447	1285884	+	Target "Motif:TcVIPER" 917 4389
1	1287805	1290931	+	Target "Motif:TcVIPER" 1238 4389
1	1889507	1890129	+	Target "Motif:TcVIPER" 5 630
1	1890209	1893838	+	Target "Motif:TcVIPER" 485 4224
1	1890209	1893838	+	Target "Motif:TcVIPER" 485 4224
1	1902317	1902939	+	Target "Motif:TcVIPER" 5 630
1	1903019	1903296	+	Target "Motif:TcVIPER" 804 1057
1	2025998	2026040	-	Target "Motif:TcVIPER" 4174 4214
1	2526175	2526238	-	Target "Motif:TcVIPER" 4210 4274
1	2530391	2533479	-	Target "Motif:TcVIPER" 1074 4209
1	2530391	2533479	-	Target "Motif:TcVIPER" 1074 4209
1	2530391	2533479	-	Target "Motif:TcVIPER" 1074 4209
1	2530391	2533479	-	Target "Motif:TcVIPER" 1074 4209

1	2530391	2533479	-	Target "Motif:TcVIPER" 1074 4209
1	2684975	2685423	+	Target "Motif:TcVIPER" 4167 4389
1	3103921	3104139	+	Target "Motif:TcVIPER" 4179 4389
10	525983	526053	+	Target "Motif:TcVIPER" 4326 4389
10	727041	727112	-	Target "Motif:TcVIPER" 4326 4390
10	903254	906691	-	Target "Motif:TcVIPER" 914 4390
10	903254	906691	-	Target "Motif:TcVIPER" 914 4390
10	903254	906691	-	Target "Motif:TcVIPER" 914 4390
10	906781	907408	-	Target "Motif:TcVIPER" 16 692
10	907950	912293	+	Target "Motif:TcVIPER" 1 4390
10	907950	912293	+	Target "Motif:TcVIPER" 1 4390
10	977680	977717	+	Target "Motif:TcVIPER" 7 46
10	977908	978491	+	Target "Motif:TcVIPER" 47 692
10	978580	981769	+	Target "Motif:TcVIPER" 916 4224
10	978580	981769	+	Target "Motif:TcVIPER" 916 4224
10	1067454	1071775	+	Target "Motif:TcVIPER" 1 4390
10	1067454	1071775	+	Target "Motif:TcVIPER" 1 4390
10	1067454	1071775	+	Target "Motif:TcVIPER" 1 4390
10	1067454	1071775	+	Target "Motif:TcVIPER" 1 4390
10	1067454	1071775	+	Target "Motif:TcVIPER" 1 4390
10	1067454	1071775	+	Target "Motif:TcVIPER" 1 4390
11	150211	153210	-	Target "Motif:TcVIPER" 1169 4182
11	153242	153863	-	Target "Motif:TcVIPER" 1 620
11	153242	153863	-	Target "Motif:TcVIPER" 1 620
11	688436	688507	-	Target "Motif:TcVIPER" 4321 4389
11	712254	713097	-	Target "Motif:TcVIPER" 3516 4390
11	713113	713730	-	Target "Motif:TcVIPER" 2 629
11	772523	773392	-	Target "Motif:TcVIPER" 3516 4390
11	773408	773850	-	Target "Motif:TcVIPER" 184 629
12	34475	34596	-	Target "Motif:TcVIPER" 4275 4389
12	45557	45769	-	Target "Motif:TcVIPER" 4179 4389
12	53228	53442	-	Target "Motif:TcVIPER" 4177 4389
12	76713	78856	-	Target "Motif:TcVIPER" 2053 4216
12	78862	79711	-	Target "Motif:TcVIPER" 917 1772
12	79871	80505	-	Target "Motif:TcVIPER" 1 634
12	82984	85115	-	Target "Motif:TcVIPER" 2053 4216
12	85123	85967	-	Target "Motif:TcVIPER" 917 1769
12	86127	86755	-	Target "Motif:TcVIPER" 1 634
12	93621	93831	-	Target "Motif:TcVIPER" 4323 4389
12	93832	94675	-	Target "Motif:TcVIPER" 3385 4231
12	97322	97393	+	Target "Motif:TcVIPER" 4323 4390
12	250176	250810	+	Target "Motif:TcVIPER" 1 634
12	250969	251816	+	Target "Motif:TcVIPER" 917 1772
12	251822	253991	+	Target "Motif:TcVIPER" 717 4216
12	251822	253991	+	Target "Motif:TcVIPER" 717 4216
12	256450	257084	+	Target "Motif:TcVIPER" 1 634

12	257245	258094	+	Target "Motif:TcVIPER" 917 1772
12	258100	260277	+	Target "Motif:TcVIPER" 717 4216
12	258100	260277	+	Target "Motif:TcVIPER" 717 4216
12	262744	263378	+	Target "Motif:TcVIPER" 1 634
12	263539	264383	+	Target "Motif:TcVIPER" 917 1769
12	264391	266557	+	Target "Motif:TcVIPER" 717 4216
12	275268	279510	-	Target "Motif:TcVIPER" 3 4236
12	275268	279510	-	Target "Motif:TcVIPER" 3 4236
12	339975	340606	+	Target "Motif:TcVIPER" 1 634
12	340765	341614	+	Target "Motif:TcVIPER" 917 1772
12	341620	343756	+	Target "Motif:TcVIPER" 2053 4216
12	346221	346848	+	Target "Motif:TcVIPER" 1 630
12	347008	347403	+	Target "Motif:TcVIPER" 917 1304
12	348477	348923	+	Target "Motif:TcVIPER" 1309 1772
12	348929	351063	+	Target "Motif:TcVIPER" 2053 4216
12	364685	365317	+	Target "Motif:TcVIPER" 1 634
12	365385	366317	+	Target "Motif:TcVIPER" 797 1772
12	366323	368455	+	Target "Motif:TcVIPER" 2053 4216
12	370936	371570	+	Target "Motif:TcVIPER" 1 634
12	371637	372578	+	Target "Motif:TcVIPER" 797 1772
12	372584	374730	+	Target "Motif:TcVIPER" 2053 4216
12	388552	392797	-	Target "Motif:TcVIPER" 3 4236
12	388552	392797	-	Target "Motif:TcVIPER" 3 4236
12	399726	400090	+	Target "Motif:TcVIPER" 3847 4216
12	940213	941732	+	Target "Motif:TcVIPER" 1953 3485
12	942203	942317	-	Target "Motif:TcVIPER" 4100 4216
12	947472	947637	-	Target "Motif:TcVIPER" 1953 2121
13	231581	234562	-	Target "Motif:TcVIPER" 1372 4389
13	231581	234562	-	Target "Motif:TcVIPER" 1372 4389
13	234576	234957	-	Target "Motif:TcVIPER" 917 1300
13	235168	235750	-	Target "Motif:TcVIPER" 52 620
13	315339	315511	-	Target "Motif:TcVIPER" 1381 1521
13	315528	316082	-	Target "Motif:TcVIPER" 770 1301
13	316130	316685	-	Target "Motif:TcVIPER" 4 634
13	541229	541484	+	Target "Motif:TcVIPER" 4167 4236
13	568301	570280	-	Target "Motif:TcVIPER" 2410 4389
13	570257	570543	-	Target "Motif:TcVIPER" 1787 2077
13	570547	571664	-	Target "Motif:TcVIPER" 128 1275
13	577234	579600	+	Target "Motif:TcVIPER" 444 4390
14	265683	268852	+	Target "Motif:TcVIPER" 10 3146
14	265683	268852	+	Target "Motif:TcVIPER" 10 3146
14	265683	268852	+	Target "Motif:TcVIPER" 10 3146
14	265683	268852	+	Target "Motif:TcVIPER" 10 3146
14	265683	268852	+	Target "Motif:TcVIPER" 10 3146
14	265683	268852	+	Target "Motif:TcVIPER" 10 3146
14	336872	337457	+	Target "Motif:TcVIPER" 52 620

14	337574	338068	+	Target "Motif:TcVIPER" 819 1296
14	344765	345941	+	Target "Motif:TcVIPER" 3168 4389
14	429584	432522	-	Target "Motif:TcVIPER" 1372 4389
14	429584	432522	-	Target "Motif:TcVIPER" 1372 4389
14	432536	433036	-	Target "Motif:TcVIPER" 819 1300
14	433134	433725	-	Target "Motif:TcVIPER" 52 620
14	437712	440384	-	Target "Motif:TcVIPER" 1372 4117
14	440396	440782	-	Target "Motif:TcVIPER" 917 1300
14	440992	441583	-	Target "Motif:TcVIPER" 52 620
15	18946	23318	-	Target "Motif:TcVIPER" 1 4390
15	18946	23318	-	Target "Motif:TcVIPER" 1 4390
15	31327	34465	-	Target "Motif:TcVIPER" 1074 4221
15	85483	85554	-	Target "Motif:TcVIPER" 4322 4389
15	243093	243554	-	Target "Motif:TcVIPER" 4167 4390
15	284039	286740	+	Target "Motif:TcVIPER" 1 2737
15	378526	382769	-	Target "Motif:TcVIPER" 123 4389
15	378526	382769	-	Target "Motif:TcVIPER" 123 4389
15	378526	382769	-	Target "Motif:TcVIPER" 123 4389
15	378526	382769	-	Target "Motif:TcVIPER" 123 4389
15	389248	390447	-	Target "Motif:TcVIPER" 1 1220
15	408040	408110	-	Target "Motif:TcVIPER" 4325 4390
15	412662	412732	-	Target "Motif:TcVIPER" 4325 4390
15	417265	417335	-	Target "Motif:TcVIPER" 4325 4390
15	421846	421916	-	Target "Motif:TcVIPER" 4325 4390
15	426428	426498	-	Target "Motif:TcVIPER" 4325 4390
15	778941	778988	-	Target "Motif:TcVIPER" 4342 4390
15	779719	781753	+	Target "Motif:TcVIPER" 3 2056
15	779719	781753	+	Target "Motif:TcVIPER" 3 2056
15	779719	781753	+	Target "Motif:TcVIPER" 3 2056
15	933941	937349	-	Target "Motif:TcVIPER" 786 4209
15	933941	937349	-	Target "Motif:TcVIPER" 786 4209
15	937365	937884	-	Target "Motif:TcVIPER" 243 806
15	948314	948753	-	Target "Motif:TcVIPER" 4167 4390
16	1016	5252	+	Target "Motif:TcVIPER" 1 4267
16	532472	533518	-	Target "Motif:TcVIPER" 3336 4389
16	538317	540270	-	Target "Motif:TcVIPER" 1372 3347
16	540284	540669	-	Target "Motif:TcVIPER" 917 1300
16	540882	541457	-	Target "Motif:TcVIPER" 52 620
16	552716	554166	+	Target "Motif:TcVIPER" 2900 4376
16	563465	567768	-	Target "Motif:TcVIPER" 1 4390
16	578608	582948	+	Target "Motif:TcVIPER" 1 4390
16	668089	668118	+	Target "Motif:TcVIPER" 4377 4390
16	747016	747184	-	Target "Motif:TcVIPER" 4271 4390
16	929859	931449	-	Target "Motif:TcVIPER" 2807 4387
16	985639	989996	-	Target "Motif:TcVIPER" 1 4390
16	985639	989996	-	Target "Motif:TcVIPER" 1 4390

16	1115432	1118877	-	Target "Motif:TcVIPER" 935 4390
16	1115432	1118877	-	Target "Motif:TcVIPER" 935 4390
16	1115432	1118877	-	Target "Motif:TcVIPER" 935 4390
16	1115432	1118877	-	Target "Motif:TcVIPER" 935 4390
16	1115432	1118877	-	Target "Motif:TcVIPER" 935 4390
16	1118911	1119674	-	Target "Motif:TcVIPER" 12 782
16	1291859	1292309	+	Target "Motif:TcVIPER" 4167 4390
18	210756	213747	-	Target "Motif:TcVIPER" 1372 4389
18	210756	213747	-	Target "Motif:TcVIPER" 1372 4389
18	210756	213747	-	Target "Motif:TcVIPER" 1372 4389
18	213761	214135	-	Target "Motif:TcVIPER" 917 1300
18	214345	214924	-	Target "Motif:TcVIPER" 52 620
18	225845	226536	-	Target "Motif:TcVIPER" 1169 1854
18	226554	227172	-	Target "Motif:TcVIPER" 1 634
18	251911	254868	-	Target "Motif:TcVIPER" 1372 4389
18	254882	255376	-	Target "Motif:TcVIPER" 819 1300
18	255498	256027	-	Target "Motif:TcVIPER" 66 594
18	571096	571736	+	Target "Motif:TcVIPER" 5 630
18	571788	575323	+	Target "Motif:TcVIPER" 717 4264
18	580298	580936	+	Target "Motif:TcVIPER" 5 630
18	581026	583454	+	Target "Motif:TcVIPER" 816 3239
18	581026	583454	+	Target "Motif:TcVIPER" 816 3239
18	581026	583454	+	Target "Motif:TcVIPER" 816 3239
18	581026	583454	+	Target "Motif:TcVIPER" 816 3239
18	581026	583454	+	Target "Motif:TcVIPER" 816 3239
18	588480	589164	+	Target "Motif:TcVIPER" 5 686
18	589216	592726	+	Target "Motif:TcVIPER" 717 4264
18	597696	598374	+	Target "Motif:TcVIPER" 5 686
18	598436	599022	+	Target "Motif:TcVIPER" 770 1335
18	603854	604543	+	Target "Motif:TcVIPER" 5 686
18	604595	608099	+	Target "Motif:TcVIPER" 770 4264
18	604595	608099	+	Target "Motif:TcVIPER" 770 4264
19	29724	29791	-	Target "Motif:TcVIPER" 4326 4389
19	119322	119992	-	Target "Motif:TcVIPER" 3720 4390
19	126951	127021	-	Target "Motif:TcVIPER" 4323 4389
19	314608	318568	-	Target "Motif:TcVIPER" 443 4390
19	314608	318568	-	Target "Motif:TcVIPER" 443 4390
19	399153	399758	+	Target "Motif:TcVIPER" 3 630
19	399845	400115	+	Target "Motif:TcVIPER" 813 1066
19	400115	402260	+	Target "Motif:TcVIPER" 2234 4389
19	420128	420573	+	Target "Motif:TcVIPER" 4167 4390
2	474899	474961	+	Target "Motif:TcVIPER" 1801 1858
2	476229	476444	+	Target "Motif:TcVIPER" 4177 4388
2	641989	642056	+	Target "Motif:TcVIPER" 4326 4389
2	652628	652695	-	Target "Motif:TcVIPER" 4326 4389
2	903393	906241	-	Target "Motif:TcVIPER" 1372 4221

2	906255	906761	-	Target "Motif:TcVIPER" 819 1300
2	906845	907481	-	Target "Motif:TcVIPER" 2 634
2	998505	1002944	+	Target "Motif:TcVIPER" 27 4390
2	998505	1002944	+	Target "Motif:TcVIPER" 27 4390
2	998505	1002944	+	Target "Motif:TcVIPER" 27 4390
2	1035844	1037408	+	Target "Motif:TcVIPER" 19 1525
2	1037399	1039751	+	Target "Motif:TcVIPER" 1864 4223
2	1098216	1098428	+	Target "Motif:TcVIPER" 4185 4389
2	1128639	1128682	+	Target "Motif:TcVIPER" 1 44
2	1128869	1133037	+	Target "Motif:TcVIPER" 45 4389
2	1128869	1133037	+	Target "Motif:TcVIPER" 45 4389
2	1290660	1291555	+	Target "Motif:TcVIPER" 3441 4389
2	1409104	1412321	-	Target "Motif:TcVIPER" 1074 4389
2	1433150	1433591	-	Target "Motif:TcVIPER" 1 447
2	1437317	1441308	-	Target "Motif:TcVIPER" 160 4209
2	1437317	1441308	-	Target "Motif:TcVIPER" 160 4209
2	1437317	1441308	-	Target "Motif:TcVIPER" 160 4209
2	1544550	1546490	-	Target "Motif:TcVIPER" 1081 3063
2	1641223	1641657	+	Target "Motif:TcVIPER" 4167 4390
2	1728704	1729542	+	Target "Motif:TcVIPER" 1 806
2	1729591	1732997	+	Target "Motif:TcVIPER" 823 4223
2	1729591	1732997	+	Target "Motif:TcVIPER" 823 4223
20	213278	213410	+	Target "Motif:TcVIPER" 452 586
20	213540	214054	+	Target "Motif:TcVIPER" 819 1307
20	214068	216990	+	Target "Motif:TcVIPER" 442 4270
20	214068	216990	+	Target "Motif:TcVIPER" 442 4270
20	214068	216990	+	Target "Motif:TcVIPER" 442 4270
20	214068	216990	+	Target "Motif:TcVIPER" 442 4270
20	214068	216990	+	Target "Motif:TcVIPER" 442 4270
20	214068	216990	+	Target "Motif:TcVIPER" 442 4270
20	343094	343554	-	Target "Motif:TcVIPER" 4167 4390
20	391806	391935	+	Target "Motif:TcVIPER" 453 586
20	392066	392578	+	Target "Motif:TcVIPER" 819 1307
20	392592	395077	+	Target "Motif:TcVIPER" 1379 4028
20	392592	395077	+	Target "Motif:TcVIPER" 1379 4028
20	392592	395077	+	Target "Motif:TcVIPER" 1379 4028
20	392592	395077	+	Target "Motif:TcVIPER" 1379 4028
20	392592	395077	+	Target "Motif:TcVIPER" 1379 4028
20	542578	542707	+	Target "Motif:TcVIPER" 457 586
20	542839	543353	+	Target "Motif:TcVIPER" 819 1307
20	542839	543353	+	Target "Motif:TcVIPER" 819 1307
20	543367	546285	+	Target "Motif:TcVIPER" 442 4270
20	543367	546285	+	Target "Motif:TcVIPER" 442 4270
20	543367	546285	+	Target "Motif:TcVIPER" 442 4270
20	726165	726494	+	Target "Motif:TcVIPER" 4177 4389
21	123791	128151	-	Target "Motif:TcVIPER" 1 4390

21	141114	142612	-	Target "Motif:TcVIPER" 2884 4389
21	145990	150340	+	Target "Motif:TcVIPER" 1 4389
21	145990	150340	+	Target "Motif:TcVIPER" 1 4389
21	145990	150340	+	Target "Motif:TcVIPER" 1 4389
21	145990	150340	+	Target "Motif:TcVIPER" 1 4389
22	4811	5348	+	Target "Motif:TcVIPER" 1 534
22	13342	13784	+	Target "Motif:TcVIPER" 4167 4389
22	13784	14339	+	Target "Motif:TcVIPER" 127 692
22	14566	17865	+	Target "Motif:TcVIPER" 917 4242
23	53141	56059	-	Target "Motif:TcVIPER" 1372 4389
23	56072	56582	-	Target "Motif:TcVIPER" 808 1297
23	56667	57061	-	Target "Motif:TcVIPER" 225 620
23	139629	140219	+	Target "Motif:TcVIPER" 52 620
23	140317	140820	+	Target "Motif:TcVIPER" 819 1300
23	140834	142596	+	Target "Motif:TcVIPER" 1372 3146
23	267582	268125	+	Target "Motif:TcVIPER" 66 594
23	268362	268745	+	Target "Motif:TcVIPER" 917 1300
23	275812	276400	+	Target "Motif:TcVIPER" 52 620
23	276613	276996	+	Target "Motif:TcVIPER" 917 1300
23	277010	279912	+	Target "Motif:TcVIPER" 1372 4389
23	277010	279912	+	Target "Motif:TcVIPER" 1372 4389
23	277010	279912	+	Target "Motif:TcVIPER" 1372 4389
23	277010	279912	+	Target "Motif:TcVIPER" 1372 4389
23	277010	279912	+	Target "Motif:TcVIPER" 1372 4389
23	277010	279912	+	Target "Motif:TcVIPER" 1372 4389
23	277010	279912	+	Target "Motif:TcVIPER" 1372 4389
23	661634	663936	+	Target "Motif:TcVIPER" 2040 4390
23	688606	692938	+	Target "Motif:TcVIPER" 1 4390
23	688606	692938	+	Target "Motif:TcVIPER" 1 4390
23	688606	692938	+	Target "Motif:TcVIPER" 1 4390
23	688606	692938	+	Target "Motif:TcVIPER" 1 4390
23	688606	692938	+	Target "Motif:TcVIPER" 1 4390
23	688606	692938	+	Target "Motif:TcVIPER" 1 4390
23	688606	692938	+	Target "Motif:TcVIPER" 1 4390
23	696598	696809	+	Target "Motif:TcVIPER" 4185 4385
24	71818	76166	-	Target "Motif:TcVIPER" 1 4390
24	71818	76166	-	Target "Motif:TcVIPER" 1 4390
24	109376	109446	-	Target "Motif:TcVIPER" 4323 4389
24	259405	260088	-	Target "Motif:TcVIPER" 3683 4378
24	281283	281969	-	Target "Motif:TcVIPER" 3688 4379
24	325428	325498	+	Target "Motif:TcVIPER" 4323 4389
24	387638	387709	+	Target "Motif:TcVIPER" 4321 4389
24	464964	465034	-	Target "Motif:TcVIPER" 4323 4389
24	489881	490141	-	Target "Motif:TcVIPER" 1372 1634
24	490153	490653	-	Target "Motif:TcVIPER" 819 1300
24	490750	491196	-	Target "Motif:TcVIPER" 162 620

24	602561	603093	-	Target "Motif:TcVIPER" 5 532
24	679125	679365	+	Target "Motif:TcVIPER" 4179 4389
24	686693	687133	+	Target "Motif:TcVIPER" 4167 4389
24	687141	688681	+	Target "Motif:TcVIPER" 27 1525
24	688672	690995	+	Target "Motif:TcVIPER" 717 4209
24	688672	690995	+	Target "Motif:TcVIPER" 717 4209
24	691033	691103	+	Target "Motif:TcVIPER" 767 838
24	692066	692511	+	Target "Motif:TcVIPER" 4167 4389
25	0	2859	-	Target "Motif:TcVIPER" 1 2942
25	0	2859	-	Target "Motif:TcVIPER" 1 2942
25	0	2859	-	Target "Motif:TcVIPER" 1 2942
25	0	2859	-	Target "Motif:TcVIPER" 1 2942
25	0	2859	-	Target "Motif:TcVIPER" 1 2942
25	227384	230727	-	Target "Motif:TcVIPER" 914 4386
25	230800	231438	-	Target "Motif:TcVIPER" 7 750
25	674297	677568	-	Target "Motif:TcVIPER" 914 4386
25	677656	678278	-	Target "Motif:TcVIPER" 7 692
25	680399	681115	+	Target "Motif:TcVIPER" 7 692
25	680493	680536	+	Target "Motif:TcVIPER" 511 683
25	681204	684533	+	Target "Motif:TcVIPER" 914 4386
25	681204	684533	+	Target "Motif:TcVIPER" 914 4386
26	152173	156176	+	Target "Motif:TcVIPER" 1 4024
26	551036	551393	+	Target "Motif:TcVIPER" 4177 4389
26	558767	559203	+	Target "Motif:TcVIPER" 4167 4389
26	559203	563344	+	Target "Motif:TcVIPER" 19 4223
26	668267	668717	+	Target "Motif:TcVIPER" 4167 4390
27	1188	1245	-	Target "Motif:TcVIPER" 4335 4390
27	1621	2245	+	Target "Motif:TcVIPER" 5 630
27	2254	5796	+	Target "Motif:TcVIPER" 717 4241
27	2254	5796	+	Target "Motif:TcVIPER" 717 4241
27	2254	5796	+	Target "Motif:TcVIPER" 717 4241
27	10415	10472	-	Target "Motif:TcVIPER" 4335 4390
27	10858	11494	+	Target "Motif:TcVIPER" 5 630
27	11503	15178	+	Target "Motif:TcVIPER" 762 4390
27	11503	15178	+	Target "Motif:TcVIPER" 762 4390
27	183325	183395	-	Target "Motif:TcVIPER" 4325 4390
27	187869	187939	-	Target "Motif:TcVIPER" 4325 4390
27	304575	305024	-	Target "Motif:TcVIPER" 4167 4390
27	443107	443557	+	Target "Motif:TcVIPER" 4167 4388
27	488542	489008	+	Target "Motif:TcVIPER" 4167 4390
27	509918	510341	+	Target "Motif:TcVIPER" 4177 4389
27	537005	537219	+	Target "Motif:TcVIPER" 4177 4389
28	312786	312823	+	Target "Motif:TcVIPER" 1 40
28	312997	314176	+	Target "Motif:TcVIPER" 41 1326
28	312997	314176	+	Target "Motif:TcVIPER" 41 1326
28	314168	316390	+	Target "Motif:TcVIPER" 1964 4224

28	386111	386140	+	Target "Motif:TcVIPER" 1 32
28	386317	387474	+	Target "Motif:TcVIPER" 33 1325
28	387467	389848	+	Target "Motif:TcVIPER" 1964 4390
28	427322	428048	+	Target "Motif:TcVIPER" 5 762
28	428319	429293	+	Target "Motif:TcVIPER" 1003 1973
29	123419	123863	+	Target "Motif:TcVIPER" 4167 4390
29	123863	125540	+	Target "Motif:TcVIPER" 138 1810
29	125537	127985	+	Target "Motif:TcVIPER" 1928 4390
29	136578	138120	+	Target "Motif:TcVIPER" 277 1810
29	138115	140560	+	Target "Motif:TcVIPER" 1926 4390
29	138115	140560	+	Target "Motif:TcVIPER" 1926 4390
29	141365	141807	+	Target "Motif:TcVIPER" 4167 4390
29	141807	143483	+	Target "Motif:TcVIPER" 138 1810
29	141807	143483	+	Target "Motif:TcVIPER" 138 1810
29	141807	143483	+	Target "Motif:TcVIPER" 138 1810
29	143480	145926	+	Target "Motif:TcVIPER" 1928 4390
29	143480	145926	+	Target "Motif:TcVIPER" 1928 4390
29	147164	148703	+	Target "Motif:TcVIPER" 277 1810
29	148698	151145	+	Target "Motif:TcVIPER" 1926 4390
29	151950	152395	+	Target "Motif:TcVIPER" 4167 4390
29	152395	154069	+	Target "Motif:TcVIPER" 138 1810
29	154064	156496	+	Target "Motif:TcVIPER" 1926 4390
29	302629	307003	+	Target "Motif:TcVIPER" 17 4389
29	315883	320262	-	Target "Motif:TcVIPER" 17 4389
29	315883	320262	-	Target "Motif:TcVIPER" 17 4389
29	395405	395617	+	Target "Motif:TcVIPER" 4179 4389
29	497365	497515	+	Target "Motif:TcVIPER" 477 626
29	503480	507831	-	Target "Motif:TcVIPER" 2 4388
29	509499	513847	+	Target "Motif:TcVIPER" 1 4388
3	10	991	-	Target "Motif:TcVIPER" 20 979
3	8811	9023	-	Target "Motif:TcVIPER" 4177 4389
3	34771	39019	-	Target "Motif:TcVIPER" 19 4223
3	34771	39019	-	Target "Motif:TcVIPER" 19 4223
3	34771	39019	-	Target "Motif:TcVIPER" 19 4223
3	34771	39019	-	Target "Motif:TcVIPER" 19 4223
3	34771	39019	-	Target "Motif:TcVIPER" 19 4223
3	41497	41563	-	Target "Motif:TcVIPER" 4333 4390
3	70095	70561	-	Target "Motif:TcVIPER" 4167 4390
3	645341	646117	-	Target "Motif:TcVIPER" 10 800
3	669654	669721	-	Target "Motif:TcVIPER" 4326 4389
3	685477	687126	-	Target "Motif:TcVIPER" 1498 3146
3	685477	687126	-	Target "Motif:TcVIPER" 1498 3146
3	685477	687126	-	Target "Motif:TcVIPER" 1498 3146
3	685477	687126	-	Target "Motif:TcVIPER" 1498 3146
3	687095	688461	-	Target "Motif:TcVIPER" 10 1345
3	1072836	1073132	+	Target "Motif:TcVIPER" 4167 4389

3	1629513	1633780	+	Target "Motif:TcVIPER" 1 4267
3	1629513	1633780	+	Target "Motif:TcVIPER" 1 4267
30	146542	146620	-	Target "Motif:TcVIPER" 4320 4390
30	259395	259473	+	Target "Motif:TcVIPER" 4320 4390
30	465769	466453	-	Target "Motif:TcVIPER" 3689 4379
30	491631	492316	+	Target "Motif:TcVIPER" 3689 4379
30	515478	517144	+	Target "Motif:TcVIPER" 2694 4389
31	287543	288172	+	Target "Motif:TcVIPER" 20 630
31	288379	288645	+	Target "Motif:TcVIPER" 918 1178
31	292922	293549	+	Target "Motif:TcVIPER" 20 630
31	293638	297081	+	Target "Motif:TcVIPER" 717 4240
31	293638	297081	+	Target "Motif:TcVIPER" 717 4240
31	293638	297081	+	Target "Motif:TcVIPER" 717 4240
31	293638	297081	+	Target "Motif:TcVIPER" 717 4240
31	297146	297212	+	Target "Motif:TcVIPER" 4326 4389
31	303950	304568	+	Target "Motif:TcVIPER" 20 630
31	304657	308085	+	Target "Motif:TcVIPER" 717 4240
31	304657	308085	+	Target "Motif:TcVIPER" 717 4240
31	304657	308085	+	Target "Motif:TcVIPER" 717 4240
31	304657	308085	+	Target "Motif:TcVIPER" 717 4240
31	308150	308216	+	Target "Motif:TcVIPER" 4326 4389
31	315028	315655	+	Target "Motif:TcVIPER" 20 630
31	315707	316397	+	Target "Motif:TcVIPER" 770 1491
31	316475	319125	+	Target "Motif:TcVIPER" 717 4240
31	319190	319257	+	Target "Motif:TcVIPER" 4326 4389
31	446546	446612	+	Target "Motif:TcVIPER" 4326 4389
32	13819	14398	+	Target "Motif:TcVIPER" 5 679
32	21417	21879	+	Target "Motif:TcVIPER" 400 913
32	327219	329315	-	Target "Motif:TcVIPER" 2226 4389
32	334152	337407	-	Target "Motif:TcVIPER" 1074 4389
32	381362	385565	-	Target "Motif:TcVIPER" 47 4390
32	385755	385798	-	Target "Motif:TcVIPER" 1 46
32	441238	445451	-	Target "Motif:TcVIPER" 96 4223
32	441238	445451	-	Target "Motif:TcVIPER" 96 4223
32	468206	468655	-	Target "Motif:TcVIPER" 4167 4390
32	609068	609096	-	Target "Motif:TcVIPER" 4361 4389
32	609485	613700	-	Target "Motif:TcVIPER" 1 4147
32	609485	613700	-	Target "Motif:TcVIPER" 1 4147
32	609485	613700	-	Target "Motif:TcVIPER" 1 4147
32	609485	613700	-	Target "Motif:TcVIPER" 1 4147
32	648811	653062	+	Target "Motif:TcVIPER" 123 4389
32	648811	653062	+	Target "Motif:TcVIPER" 123 4389
32	658345	659792	+	Target "Motif:TcVIPER" 2914 4389
32	665091	666542	+	Target "Motif:TcVIPER" 2914 4383
32	806144	806442	+	Target "Motif:TcVIPER" 230 612
32	806512	807065	+	Target "Motif:TcVIPER" 770 1300

32	807084	809895	+	Target "Motif:TcVIPER" 1381 4185
32	809964	810033	+	Target "Motif:TcVIPER" 4320 4389
32	982530	983977	+	Target "Motif:TcVIPER" 2914 4383
33	209000	213143	-	Target "Motif:TcVIPER" 1 4156
33	209000	213143	-	Target "Motif:TcVIPER" 1 4156
33	209000	213143	-	Target "Motif:TcVIPER" 1 4156
33	209000	213143	-	Target "Motif:TcVIPER" 1 4156
33	209000	213143	-	Target "Motif:TcVIPER" 1 4156
33	264841	268987	+	Target "Motif:TcVIPER" 1 4156
33	264841	268987	+	Target "Motif:TcVIPER" 1 4156
33	264841	268987	+	Target "Motif:TcVIPER" 1 4156
33	264841	268987	+	Target "Motif:TcVIPER" 1 4156
33	264841	268987	+	Target "Motif:TcVIPER" 1 4156
33	269229	269612	-	Target "Motif:TcVIPER" 4167 4390
33	269614	270038	-	Target "Motif:TcVIPER" 4167 4390
33	288637	289128	-	Target "Motif:TcVIPER" 4167 4390
33	472229	475362	+	Target "Motif:TcVIPER" 1074 4221
33	472229	475362	+	Target "Motif:TcVIPER" 1074 4221
34	167924	172352	-	Target "Motif:TcVIPER" 5 4267
34	167924	172352	-	Target "Motif:TcVIPER" 5 4267
34	167924	172352	-	Target "Motif:TcVIPER" 5 4267
34	167924	172352	-	Target "Motif:TcVIPER" 5 4267
34	167924	172352	-	Target "Motif:TcVIPER" 5 4267
34	167924	172352	-	Target "Motif:TcVIPER" 5 4267
34	247895	250417	-	Target "Motif:TcVIPER" 1846 4386
34	415556	419869	+	Target "Motif:TcVIPER" 12 4390
34	415556	419869	+	Target "Motif:TcVIPER" 12 4390
34	415556	419869	+	Target "Motif:TcVIPER" 12 4390
34	415556	419869	+	Target "Motif:TcVIPER" 12 4390
34	415556	419869	+	Target "Motif:TcVIPER" 12 4390
34	486337	486767	+	Target "Motif:TcVIPER" 4167 4389
35	58290	58357	+	Target "Motif:TcVIPER" 4326 4389
35	77914	77981	+	Target "Motif:TcVIPER" 4326 4389
35	121336	121773	-	Target "Motif:TcVIPER" 4167 4389
35	161675	162718	-	Target "Motif:TcVIPER" 3336 4389
35	167505	169466	-	Target "Motif:TcVIPER" 1372 3347
35	169480	169864	-	Target "Motif:TcVIPER" 917 1300
35	170078	170518	-	Target "Motif:TcVIPER" 162 620
35	184052	185214	-	Target "Motif:TcVIPER" 3212 4389
35	191989	192275	-	Target "Motif:TcVIPER" 1372 1666
35	192289	192667	-	Target "Motif:TcVIPER" 917 1300
35	192877	193455	-	Target "Motif:TcVIPER" 52 620
35	381560	382773	-	Target "Motif:TcVIPER" 2972 4210
35	383032	386008	-	Target "Motif:TcVIPER" 1 2988
35	383032	386008	-	Target "Motif:TcVIPER" 1 2988
35	390048	390118	-	Target "Motif:TcVIPER" 4323 4389

35	390254	391466	-	Target "Motif:TcVIPER" 2972 4210
35	391726	394704	-	Target "Motif:TcVIPER" 1 2988
35	391726	394704	-	Target "Motif:TcVIPER" 1 2988
35	391726	394704	-	Target "Motif:TcVIPER" 1 2988
35	391726	394704	-	Target "Motif:TcVIPER" 1 2988
35	397772	400751	+	Target "Motif:TcVIPER" 1 2988
35	397772	400751	+	Target "Motif:TcVIPER" 1 2988
35	397772	400751	+	Target "Motif:TcVIPER" 1 2988
35	401009	402222	+	Target "Motif:TcVIPER" 2972 4210
35	402356	402426	+	Target "Motif:TcVIPER" 4323 4389
35	505416	505497	-	Target "Motif:TcVIPER" 4323 4389
35	505646	506864	-	Target "Motif:TcVIPER" 2972 4214
35	507122	510103	-	Target "Motif:TcVIPER" 1 2988
35	507122	510103	-	Target "Motif:TcVIPER" 1 2988
35	513171	516155	+	Target "Motif:TcVIPER" 1 2988
35	516415	517632	+	Target "Motif:TcVIPER" 2972 4214
36	40228	40554	-	Target "Motif:TcVIPER" 4177 4389
36	299827	299977	-	Target "Motif:TcVIPER" 477 626
36	311812	314020	-	Target "Motif:TcVIPER" 444 4389
36	314010	315494	-	Target "Motif:TcVIPER" 718 2176
36	315582	316164	-	Target "Motif:TcVIPER" 50 620
36	435226	435675	+	Target "Motif:TcVIPER" 4167 4390
37	33129	33279	+	Target "Motif:TcVIPER" 477 626
37	38574	38707	-	Target "Motif:TcVIPER" 644 778
37	38653	41862	-	Target "Motif:TcVIPER" 922 4221
37	42202	42828	-	Target "Motif:TcVIPER" 2 620
37	44495	48673	+	Target "Motif:TcVIPER" 1 4233
37	44495	48673	+	Target "Motif:TcVIPER" 1 4233
37	44495	48673	+	Target "Motif:TcVIPER" 1 4233
37	51291	54001	+	Target "Motif:TcVIPER" 644 4241
37	61206	64083	+	Target "Motif:TcVIPER" 1512 4390
37	75947	76454	+	Target "Motif:TcVIPER" 19 705
37	76545	78710	+	Target "Motif:TcVIPER" 816 2984
37	92699	93335	+	Target "Motif:TcVIPER" 5 630
37	93344	96919	+	Target "Motif:TcVIPER" 717 4224
37	100349	102675	+	Target "Motif:TcVIPER" 1877 4224
37	124546	125178	+	Target "Motif:TcVIPER" 5 630
37	125187	128758	+	Target "Motif:TcVIPER" 717 4224
37	125187	128758	+	Target "Motif:TcVIPER" 717 4224
37	191829	191869	+	Target "Motif:TcVIPER" 3 42
37	192039	196209	+	Target "Motif:TcVIPER" 43 4233
37	204651	205246	+	Target "Motif:TcVIPER" 5 589
37	205294	208953	+	Target "Motif:TcVIPER" 485 4233
37	256698	260919	-	Target "Motif:TcVIPER" 5 4233
37	274138	277815	-	Target "Motif:TcVIPER" 485 4233
37	274138	277815	-	Target "Motif:TcVIPER" 485 4233

37	277824	278465	-	Target "Motif:TcVIPER" 5 630
37	311002	311070	-	Target "Motif:TcVIPER" 4326 4390
37	328495	329136	+	Target "Motif:TcVIPER" 5 630
37	329145	332700	+	Target "Motif:TcVIPER" 717 4233
37	341489	341556	-	Target "Motif:TcVIPER" 4326 4389
37	359285	359353	+	Target "Motif:TcVIPER" 4326 4390
37	361528	365795	+	Target "Motif:TcVIPER" 3 4224
37	361528	365795	+	Target "Motif:TcVIPER" 3 4224
37	361528	365795	+	Target "Motif:TcVIPER" 3 4224
37	361528	365795	+	Target "Motif:TcVIPER" 3 4224
37	361528	365795	+	Target "Motif:TcVIPER" 3 4224
37	361528	365795	+	Target "Motif:TcVIPER" 3 4224
37	361528	365795	+	Target "Motif:TcVIPER" 3 4224
37	365861	365929	+	Target "Motif:TcVIPER" 4326 4390
38	21273	25693	-	Target "Motif:TcVIPER" 2 4389
38	42541	46909	-	Target "Motif:TcVIPER" 60 4389
38	48971	52223	-	Target "Motif:TcVIPER" 60 3290
38	48971	52223	-	Target "Motif:TcVIPER" 60 3290
38	48971	52223	-	Target "Motif:TcVIPER" 60 3290
38	306185	306919	+	Target "Motif:TcVIPER" 60 806
38	306185	306919	+	Target "Motif:TcVIPER" 60 806
38	306960	310557	+	Target "Motif:TcVIPER" 814 4389
38	306960	310557	+	Target "Motif:TcVIPER" 814 4389
38	306960	310557	+	Target "Motif:TcVIPER" 814 4389
38	419991	424160	+	Target "Motif:TcVIPER" 14 4209
38	432668	432733	+	Target "Motif:TcVIPER" 4210 4274
39	150164	153473	+	Target "Motif:TcVIPER" 1074 4388
39	150164	153473	+	Target "Motif:TcVIPER" 1074 4388
39	150164	153473	+	Target "Motif:TcVIPER" 1074 4388
39	150164	153473	+	Target "Motif:TcVIPER" 1074 4388
39	150164	153473	+	Target "Motif:TcVIPER" 1074 4388
4	32231	32299	+	Target "Motif:TcVIPER" 4170 4237
4	46272	46677	+	Target "Motif:TcVIPER" 4167 4237
4	70389	70864	+	Target "Motif:TcVIPER" 4167 4237
4	179843	180309	+	Target "Motif:TcVIPER" 4167 4237
4	355698	355770	+	Target "Motif:TcVIPER" 4167 4233
4	572538	572970	+	Target "Motif:TcVIPER" 4167 4388
4	573012	577385	+	Target "Motif:TcVIPER" 1 4390
4	573012	577385	+	Target "Motif:TcVIPER" 1 4390
4	573012	577385	+	Target "Motif:TcVIPER" 1 4390
4	594198	594250	+	Target "Motif:TcVIPER" 1 54
4	594427	598579	+	Target "Motif:TcVIPER" 55 4389
4	637499	637972	+	Target "Motif:TcVIPER" 4167 4237
4	646122	646193	+	Target "Motif:TcVIPER" 4167 4237
4	660583	660656	+	Target "Motif:TcVIPER" 4167 4237
4	667868	668185	+	Target "Motif:TcVIPER" 4170 4237

4	688967	689289	+	Target "Motif:TcVIPER" 4167 4237
4	703068	703441	+	Target "Motif:TcVIPER" 4167 4237
4	780184	784554	+	Target "Motif:TcVIPER" 1 4390
4	780184	784554	+	Target "Motif:TcVIPER" 1 4390
4	780184	784554	+	Target "Motif:TcVIPER" 1 4390
4	791035	791465	-	Target "Motif:TcVIPER" 4172 4389
4	816787	816858	+	Target "Motif:TcVIPER" 4321 4389
4	837549	837620	-	Target "Motif:TcVIPER" 4321 4389
4	864590	864661	-	Target "Motif:TcVIPER" 4321 4389
4	882487	882558	-	Target "Motif:TcVIPER" 4321 4389
4	898575	898646	-	Target "Motif:TcVIPER" 4321 4389
4	1467982	1472360	+	Target "Motif:TcVIPER" 1 4389
4	1467982	1472360	+	Target "Motif:TcVIPER" 1 4389
4	1467982	1472360	+	Target "Motif:TcVIPER" 1 4389
40	79229	80655	-	Target "Motif:TcVIPER" 2914 4233
40	246811	251181	-	Target "Motif:TcVIPER" 1 4390
40	309658	311093	-	Target "Motif:TcVIPER" 2914 4233
40	342201	343634	+	Target "Motif:TcVIPER" 2914 4233
40	342201	343634	+	Target "Motif:TcVIPER" 2914 4233
41	277537	277552	+	Target "Motif:TcVIPER" 4345 4390
41	424822	425254	-	Target "Motif:TcVIPER" 3769 4226
41	425247	428253	-	Target "Motif:TcVIPER" 98 3102
42	30497	30564	-	Target "Motif:TcVIPER" 4326 4389
42	36928	36995	-	Target "Motif:TcVIPER" 4326 4389
42	138708	138921	-	Target "Motif:TcVIPER" 4177 4389
42	145192	145406	-	Target "Motif:TcVIPER" 4177 4389
42	160773	161679	-	Target "Motif:TcVIPER" 3291 4221
42	383713	388144	-	Target "Motif:TcVIPER" 7 4390
42	435285	438631	+	Target "Motif:TcVIPER" 68 3347
42	435285	438631	+	Target "Motif:TcVIPER" 68 3347
42	435285	438631	+	Target "Motif:TcVIPER" 68 3347
42	714616	714845	+	Target "Motif:TcVIPER" 4167 4387
42	727690	727726	+	Target "Motif:TcVIPER" 471 683
42	727931	728197	+	Target "Motif:TcVIPER" 4193 4387
42	827405	831865	+	Target "Motif:TcVIPER" 1 4387
42	827405	831865	+	Target "Motif:TcVIPER" 1 4387
42	827405	831865	+	Target "Motif:TcVIPER" 1 4387
43	76614	76695	+	Target "Motif:TcVIPER" 4319 4390
43	119149	123417	-	Target "Motif:TcVIPER" 3 4262
44	10280	11193	-	Target "Motif:TcVIPER" 3291 4221
44	15925	16494	-	Target "Motif:TcVIPER" 2735 3304
44	16489	19160	-	Target "Motif:TcVIPER" 1 2613
44	16489	19160	-	Target "Motif:TcVIPER" 1 2613
44	47364	48832	-	Target "Motif:TcVIPER" 2735 4221
44	48827	51494	-	Target "Motif:TcVIPER" 1 2613
44	48827	51494	-	Target "Motif:TcVIPER" 1 2613

44	91199	91278	+	Target "Motif:TcVIPER" 4166 4240
44	93537	93979	-	Target "Motif:TcVIPER" 4172 4390
44	153697	156272	-	Target "Motif:TcVIPER" 770 3304
44	156324	157012	-	Target "Motif:TcVIPER" 5 684
45	269753	269978	-	Target "Motif:TcVIPER" 4167 4389
45	271012	271376	-	Target "Motif:TcVIPER" 4167 4390
46	64154	67082	-	Target "Motif:TcVIPER" 1372 4389
46	67096	67482	-	Target "Motif:TcVIPER" 917 1300
46	67726	68277	-	Target "Motif:TcVIPER" 52 594
46	181833	186012	-	Target "Motif:TcVIPER" 1 4242
46	181833	186012	-	Target "Motif:TcVIPER" 1 4242
46	224089	224630	-	Target "Motif:TcVIPER" 1 534
46	351987	352205	+	Target "Motif:TcVIPER" 4179 4389
46	357128	357557	+	Target "Motif:TcVIPER" 4167 4389
46	357894	358296	+	Target "Motif:TcVIPER" 4179 4389
47	189777	194136	-	Target "Motif:TcVIPER" 1 4390
47	189777	194136	-	Target "Motif:TcVIPER" 1 4390
47	194180	194614	-	Target "Motif:TcVIPER" 4167 4388
47	201771	204808	-	Target "Motif:TcVIPER" 1 3051
47	201771	204808	-	Target "Motif:TcVIPER" 1 3051
47	201771	204808	-	Target "Motif:TcVIPER" 1 3051
47	204851	205282	-	Target "Motif:TcVIPER" 4167 4388
47	212435	216541	-	Target "Motif:TcVIPER" 1 4135
47	212435	216541	-	Target "Motif:TcVIPER" 1 4135
47	216584	217014	-	Target "Motif:TcVIPER" 4167 4388
47	243864	245577	-	Target "Motif:TcVIPER" 1 1730
47	245620	246050	-	Target "Motif:TcVIPER" 4167 4388
47	365042	365505	-	Target "Motif:TcVIPER" 4167 4237
47	394468	394693	+	Target "Motif:TcVIPER" 4167 4389
5	104090	107641	-	Target "Motif:TcVIPER" 816 4390
5	107728	108268	-	Target "Motif:TcVIPER" 7 634
5	455698	456179	+	Target "Motif:TcVIPER" 4167 4390
5	523113	527339	-	Target "Motif:TcVIPER" 3 4262
5	765100	765322	+	Target "Motif:TcVIPER" 4186 4388
5	765603	766227	+	Target "Motif:TcVIPER" 16 634
5	766440	766816	+	Target "Motif:TcVIPER" 929 1302
5	766839	769462	+	Target "Motif:TcVIPER" 1383 4212
5	780104	780732	+	Target "Motif:TcVIPER" 16 634
5	780823	781337	+	Target "Motif:TcVIPER" 820 1302
5	781360	784014	+	Target "Motif:TcVIPER" 1383 4220
5	787728	788351	+	Target "Motif:TcVIPER" 16 634
5	788553	788953	+	Target "Motif:TcVIPER" 916 1302
5	788974	791310	+	Target "Motif:TcVIPER" 1383 3910
5	825684	826253	+	Target "Motif:TcVIPER" 16 573
5	826530	826903	+	Target "Motif:TcVIPER" 931 1302
5	827011	829568	+	Target "Motif:TcVIPER" 1512 4220

5	839286	839862	+	Target "Motif:TcVIPER" 16 586
5	840139	840508	+	Target "Motif:TcVIPER" 948 1302
5	840675	840967	+	Target "Motif:TcVIPER" 1563 1855
5	840963	843159	+	Target "Motif:TcVIPER" 1975 4220
5	845720	845973	+	Target "Motif:TcVIPER" 368 620
5	846189	846574	+	Target "Motif:TcVIPER" 916 1301
5	846682	849243	+	Target "Motif:TcVIPER" 1512 4220
5	852960	853575	+	Target "Motif:TcVIPER" 16 620
5	853792	854188	+	Target "Motif:TcVIPER" 916 1302
5	854296	856557	+	Target "Motif:TcVIPER" 1512 3910
5	1184566	1188796	-	Target "Motif:TcVIPER" 25 4389
5	1188982	1189004	-	Target "Motif:TcVIPER" 1 24
6	2572	2807	-	Target "Motif:TcVIPER" 4191 4389
6	3024	3039	-	Target "Motif:TcVIPER" 4177 4190
6	32803	33235	-	Target "Motif:TcVIPER" 4167 4390
6	264382	268741	-	Target "Motif:TcVIPER" 1 4390
6	297810	302143	+	Target "Motif:TcVIPER" 1 4390
6	446453	449724	+	Target "Motif:TcVIPER" 1074 4389
6	492398	496784	-	Target "Motif:TcVIPER" 1 4390
6	492398	496784	-	Target "Motif:TcVIPER" 1 4390
6	496828	497258	-	Target "Motif:TcVIPER" 4167 4388
6	504185	506801	-	Target "Motif:TcVIPER" 1 2628
6	504185	506801	-	Target "Motif:TcVIPER" 1 2628
6	506843	507272	-	Target "Motif:TcVIPER" 4167 4388
6	717784	717852	-	Target "Motif:TcVIPER" 4172 4237
6	732516	732984	-	Target "Motif:TcVIPER" 4167 4237
6	740183	740255	-	Target "Motif:TcVIPER" 4170 4237
6	753885	754062	-	Target "Motif:TcVIPER" 4167 4237
6	761461	761528	-	Target "Motif:TcVIPER" 4173 4237
6	796498	796564	-	Target "Motif:TcVIPER" 4173 4237
6	806141	806592	-	Target "Motif:TcVIPER" 4167 4237
6	842185	846551	-	Target "Motif:TcVIPER" 1 4389
6	931711	932154	+	Target "Motif:TcVIPER" 4167 4389
6	1091235	1091694	+	Target "Motif:TcVIPER" 4167 4390
6	1283757	1283965	+	Target "Motif:TcVIPER" 4184 4389
7	13281	13740	+	Target "Motif:TcVIPER" 4167 4390
7	102942	105097	-	Target "Motif:TcVIPER" 717 4224
7	102942	105097	-	Target "Motif:TcVIPER" 717 4224
7	107098	109653	-	Target "Motif:TcVIPER" 1864 4390
7	109644	110258	-	Target "Motif:TcVIPER" 919 1525
7	110419	111196	-	Target "Motif:TcVIPER" 18 806
7	111196	111651	-	Target "Motif:TcVIPER" 4167 4388
7	119080	119292	-	Target "Motif:TcVIPER" 4179 4389
7	1115202	1115274	+	Target "Motif:TcVIPER" 4323 4390
7	1174661	1174730	+	Target "Motif:TcVIPER" 4323 4386
7	1175132	1179470	-	Target "Motif:TcVIPER" 4 4376

7	1175132	1179470	-	Target "Motif:TcVIPER" 4 4376
7	1175132	1179470	-	Target "Motif:TcVIPER" 4 4376
7	1304453	1308811	-	Target "Motif:TcVIPER" 1 4389
7	1304453	1308811	-	Target "Motif:TcVIPER" 1 4389
7	1304453	1308811	-	Target "Motif:TcVIPER" 1 4389
7	1523488	1523554	-	Target "Motif:TcVIPER" 4326 4389
7	1658736	1658765	+	Target "Motif:TcVIPER" 1 29
7	1658941	1663169	+	Target "Motif:TcVIPER" 30 4390
7	1658941	1663169	+	Target "Motif:TcVIPER" 30 4390
7	1906778	1907754	-	Target "Motif:TcVIPER" 1003 1973
7	1908026	1908610	-	Target "Motif:TcVIPER" 6 762
7	2125237	2125304	-	Target "Motif:TcVIPER" 4326 4389
8	103323	107655	+	Target "Motif:TcVIPER" 1 4390
8	103323	107655	+	Target "Motif:TcVIPER" 1 4390
8	103323	107655	+	Target "Motif:TcVIPER" 1 4390
8	136385	137073	-	Target "Motif:TcVIPER" 3683 4379
8	350496	353732	-	Target "Motif:TcVIPER" 1074 4389
8	359091	362341	-	Target "Motif:TcVIPER" 1074 4389
8	359091	362341	-	Target "Motif:TcVIPER" 1074 4389
8	359091	362341	-	Target "Motif:TcVIPER" 1074 4389
8	370518	373770	-	Target "Motif:TcVIPER" 1074 4389
8	370518	373770	-	Target "Motif:TcVIPER" 1074 4389
8	370518	373770	-	Target "Motif:TcVIPER" 1074 4389
8	370518	373770	-	Target "Motif:TcVIPER" 1074 4389
8	398604	401822	-	Target "Motif:TcVIPER" 1074 4389
8	398604	401822	-	Target "Motif:TcVIPER" 1074 4389
8	422336	425568	-	Target "Motif:TcVIPER" 1074 4389
8	682379	682867	-	Target "Motif:TcVIPER" 1074 1555
8	743625	746207	+	Target "Motif:TcVIPER" 1 2641
8	743625	746207	+	Target "Motif:TcVIPER" 1 2641
8	750158	754128	-	Target "Motif:TcVIPER" 160 4209
8	750158	754128	-	Target "Motif:TcVIPER" 160 4209
8	750158	754128	-	Target "Motif:TcVIPER" 160 4209
8	750158	754128	-	Target "Motif:TcVIPER" 160 4209
8	807125	807255	+	Target "Motif:TcVIPER" 4259 4389
8	954874	955304	+	Target "Motif:TcVIPER" 4167 4389
8	1093523	1093593	-	Target "Motif:TcVIPER" 4323 4389
8	1120326	1124266	+	Target "Motif:TcVIPER" 443 4390
8	1120326	1124266	+	Target "Motif:TcVIPER" 443 4390
8	1120326	1124266	+	Target "Motif:TcVIPER" 443 4390
9	5283	5733	+	Target "Motif:TcVIPER" 4167 4390
9	7322	7922	+	Target "Motif:TcVIPER" 19 620
9	8139	8383	+	Target "Motif:TcVIPER" 917 1155
9	88042	88287	-	Target "Motif:TcVIPER" 917 1155
9	88489	89102	-	Target "Motif:TcVIPER" 19 634
9	889520	889603	+	Target "Motif:TcVIPER" 4323 4389

9	1059129	1059343	+	Target "Motif:TcVIPER" 4178 4389
9	1110548	1110758	+	Target "Motif:TcVIPER" 4178 4389
9	1119142	1119378	+	Target "Motif:TcVIPER" 4179 4389
