# Supplementary material for: Repeat-Driven Generation of Antigenic Diversity in a Major Human Pathogen, Trypanosoma cruzi
Source: Front Cell Infect Microbiol. 2021 Mar 3;11:614665. doi: 10.3389/fcimb.2021.614665 (PMC7966520; doi:10.3389/fcimb.2021.614665)
Supplement: Supplementary file 4 [file DataSheet_4.pdf]

Supplementary table 4, part 1.

Chromosome	startR	stopR	strand	repeat
1	42888	43071	+	Target "Motif:SIRE3_TC" 576 759
1	45664	45855	+	Target "Motif:SIRE" 99 278
1	59617	59853	+	Target "Motif:SIRE3_TC" 511 744
1	62410	62595	+	Target "Motif:SIRE" 99 281
1	78843	79105	+	Target "Motif:SIRE3_TC" 511 766
1	81653	82116	+	Target "Motif:SIRE3_TC" 272 920
1	93784	94243	+	Target "Motif:SIRE3_TC" 276 920
1	101064	101122	+	Target "Motif:SIRE" 87 146
1	105349	105536	+	Target "Motif:SIRE_TC" 480 668
1	105473	105545	+	Target "Motif:SIRE" 34 102
1	204738	205116	-	Target "Motif:SIRE3_TC" 504 782
1	395175	395611	-	Target "Motif:SIRE3_TC" 504 920
1	434031	434215	+	Target "Motif:SIRE3_TC" 664 837
1	434274	434344	+	Target "Motif:SIRE" 32 98
1	540010	540168	-	Target "Motif:SIRE" 99 251
1	544878	545063	-	Target "Motif:SIRE" 99 281
1	547616	547863	-	Target "Motif:SIRE3_TC" 511 750
1	601707	601968	-	Target "Motif:SIRE3_TC" 511 766
1	610653	610844	-	Target "Motif:SIRE" 99 278
1	613383	613643	-	Target "Motif:SIRE3_TC" 511 766
1	617500	617687	-	Target "Motif:SIRE" 99 281
1	620235	620497	-	Target "Motif:SIRE3_TC" 511 766
1	629161	629350	-	Target "Motif:SIRE" 99 278
1	636965	637223	-	Target "Motif:SIRE3_TC" 511 766
1	645894	645940	-	Target "Motif:SIRE" 99 137
1	651044	651091	-	Target "Motif:SIRE" 99 142
1	653630	653874	-	Target "Motif:SIRE3_TC" 511 750
1	662546	662592	-	Target "Motif:SIRE" 99 138
1	669994	670256	-	Target "Motif:SIRE3_TC" 511 766
1	679080	679126	-	Target "Motif:SIRE" 99 139
1	681692	681936	-	Target "Motif:SIRE3_TC" 511 750
1	698110	698296	-	Target "Motif:SIRE" 99 281
1	700843	701105	-	Target "Motif:SIRE3_TC" 511 766
1	762282	762715	+	Target "Motif:SIRE3_TC" 509 918
1	806990	807439	+	Target "Motif:SIRE3_TC" 504 920
1	1004938	1005185	+	Target "Motif:SIRE" 32 282
1	1005528	1005992	+	Target "Motif:SIRE3_TC" 283 763
1	1016649	1016918	+	Target "Motif:SIRE3_TC" 503 763
1	1017524	1017973	+	Target "Motif:SIRE3_TC" 501 920
1	1019932	1020200	+	Target "Motif:SIRE3_TC" 503 763
1	1056345	1056661	+	Target "Motif:SIRE3_TC" 526 831
1	1232878	1233192	+	Target "Motif:SIRE3_TC" 492 771
1	1233207	1233424	+	Target "Motif:SIRE_TC" 464 668
1	1246012	1246059	+	Target "Motif:SIRE" 97 144
1	1254202	1254423	+	Target "Motif:SIRE" 95 310
1	1254236	1254726	+	Target "Motif:SIRE7_TC" 250 741

1	1260764	1260985	+	Target "Motif:SIRE" 95 315
1	1260985	1261251	+	Target "Motif:SIRE3_TC" 487 782
1	1263286	1263334	+	Target "Motif:SIRE" 95 150
1	1267377	1267450	+	Target "Motif:SIRE" 33 102
1	1281613	1281796	+	Target "Motif:SIRE" 93 281
1	1369141	1369322	+	Target "Motif:SIRE" 104 279
1	1459799	1460269	+	Target "Motif:SIRE3_TC" 504 919
1	1650298	1650636	-	Target "Motif:SIRE3_TC" 508 920
1	1650636	1650705	-	Target "Motif:SIRE" 98 172
1	1677854	1678320	-	Target "Motif:SIRE3_TC" 485 832
1	1842498	1843002	-	Target "Motif:SIRE3_TC" 280 920
1	1856957	1857407	-	Target "Motif:SIRE3_TC" 495 920
1	1888624	1888907	+	Target "Motif:SIRE3_TC" 511 776
1	1889459	1889646	+	Target "Motif:SIRE" 97 281
1	1901895	1902194	-	Target "Motif:SIRE3_TC" 646 923
1	1902125	1902247	-	Target "Motif:SIRE" 190 313
1	1902273	1902455	+	Target "Motif:SIRE" 101 281
1	2025801	2025986	+	Target "Motif:SIRE" 107 303
1	2682404	2682834	+	Target "Motif:SIRE3_TC" 511 915
1	2986628	2987004	+	Target "Motif:SIRE3_TC" 511 837
1	3057165	3057624	+	Target "Motif:SIRE3_TC" 494 920
1	3103693	3103921	+	Target "Motif:SIRE" 94 313
10	206144	206598	-	Target "Motif:SIRE3_TC" 495 920
10	293539	293979	+	Target "Motif:SIRE3_TC" 508 920
10	523855	524052	+	Target "Motif:SIRE" 94 281
10	524071	524329	+	Target "Motif:SIRE3_TC" 669 902
10	525623	525676	+	Target "Motif:SIRE" 94 146
10	525673	525800	-	Target "Motif:SIRE" 144 271
10	525797	525894	+	Target "Motif:SIRE3_TC" 678 774
10	526020	526064	+	Target "Motif:SIRE7_TC" 650 693
10	543017	543517	+	Target "Motif:SIRE3_TC" 339 920
10	727199	727315	-	Target "Motif:SIRE3_TC" 645 774
10	907408	907555	-	Target "Motif:SIRE" 102 241
10	907907	907950	+	Target "Motif:SIRE" 101 144
10	926261	926633	+	Target "Motif:SIRE3_TC" 504 837
10	977717	977908	+	Target "Motif:SIRE" 101 274
10	990842	991179	+	Target "Motif:SIRE3_TC" 468 774
10	996100	996547	+	Target "Motif:SIRE3_TC" 504 920
10	1067400	1067454	+	Target "Motif:SIRE" 91 146
11	153863	153912	-	Target "Motif:SIRE" 95 145
11	258295	258752	-	Target "Motif:SIRE3_TC" 257 920
11	309173	309558	+	Target "Motif:SIRE3_TC" 496 843
11	634919	635390	+	Target "Motif:SIRE3_TC" 482 920
11	688568	688910	-	Target "Motif:SIRE3_TC" 510 779
11	697427	697875	-	Target "Motif:SIRE3_TC" 504 920
11	713730	713776	-	Target "Motif:SIRE" 95 148

11	716219	716650	+	Target "Motif:SIRE3_TC" 508 920
11	745430	745866	+	Target "Motif:SIRE3_TC" 511 920
11	827100	827391	+	Target "Motif:SIRE3_TC" 480 768
11	845848	846322	+	Target "Motif:SIRE3_TC" 339 920
11	847580	847758	+	Target "Motif:SIRE3_TC" 475 629
11	847603	847761	+	Target "Motif:SIRE" 98 226
11	1014851	1015305	+	Target "Motif:SIRE3_TC" 257 920
11	1020947	1021274	+	Target "Motif:SIRE3_TC" 516 829
11	1021324	1021794	+	Target "Motif:SIRE3_TC" 257 920
12	45769	45979	-	Target "Motif:SIRE" 94 297
12	53440	53652	-	Target "Motif:SIRE" 94 297
12	76444	76517	-	Target "Motif:SIRE7_TC" 646 709
12	76471	76544	-	Target "Motif:SIRE" 37 109
12	82717	82789	-	Target "Motif:SIRE7_TC" 646 709
12	82743	82816	-	Target "Motif:SIRE" 37 109
12	95493	95566	-	Target "Motif:SIRE7_TC" 646 709
12	95520	95576	-	Target "Motif:SIRE" 54 109
12	95786	96079	+	Target "Motif:SIRE3_TC" 544 840
12	96883	97260	+	Target "Motif:SIRE3_TC" 468 838
12	254132	254205	+	Target "Motif:SIRE" 37 109
12	254159	254220	+	Target "Motif:SIRE7_TC" 646 705
12	260420	260493	+	Target "Motif:SIRE" 37 109
12	260447	260508	+	Target "Motif:SIRE7_TC" 646 705
12	266700	266773	+	Target "Motif:SIRE" 37 109
12	266727	266788	+	Target "Motif:SIRE7_TC" 646 705
12	279510	279554	-	Target "Motif:SIRE" 101 147
12	279553	279724	-	Target "Motif:SIRE" 98 272
12	343923	343996	+	Target "Motif:SIRE" 37 109
12	343950	344023	+	Target "Motif:SIRE7_TC" 646 709
12	351232	351304	+	Target "Motif:SIRE" 37 109
12	351258	351330	+	Target "Motif:SIRE7_TC" 646 709
12	368622	368695	+	Target "Motif:SIRE" 37 109
12	368649	368722	+	Target "Motif:SIRE7_TC" 646 709
12	374899	374972	+	Target "Motif:SIRE" 37 109
12	374926	374999	+	Target "Motif:SIRE7_TC" 646 709
12	392797	392841	-	Target "Motif:SIRE" 101 147
12	392854	393011	-	Target "Motif:SIRE" 98 257
12	400256	400330	+	Target "Motif:SIRE" 37 111
12	941933	942031	-	Target "Motif:SIRE7_TC" 620 709
12	941960	942033	-	Target "Motif:SIRE" 37 109
12	1001433	1001951	+	Target "Motif:SIRE3_TC" 463 920
13	316685	316740	-	Target "Motif:SIRE" 93 145
13	396933	397250	+	Target "Motif:SIRE3_TC" 511 771
13	397232	397440	+	Target "Motif:SIRE_TC" 480 688
13	541442	541513	+	Target "Motif:SIRE" 31 101
13	571654	571932	-	Target "Motif:SIRE7_TC" 396 693

13	614662	614992	-	Target "Motif:SIRE3_TC" 505 828
13	619922	620383	-	Target "Motif:SIRE3_TC" 474 920
13	631180	631485	-	Target "Motif:SIRE3_TC" 707 920
13	805733	806193	-	Target "Motif:SIRE3_TC" 503 920
13	807926	808247	-	Target "Motif:SIRE3_TC" 514 777
13	809797	809815	-	Target "Motif:SIRE" 73 90
13	809815	810070	-	Target "Motif:SIRE3_TC" 669 920
13	810022	810240	-	Target "Motif:SIRE" 98 313
13	853768	854229	-	Target "Motif:SIRE3_TC" 503 920
13	855957	856124	-	Target "Motif:SIRE3_TC" 590 777
13	856071	856291	-	Target "Motif:SIRE" 94 312
13	857811	857829	-	Target "Motif:SIRE" 73 90
13	857829	858081	-	Target "Motif:SIRE3_TC" 670 920
13	858034	858250	-	Target "Motif:SIRE" 98 313
13	866100	866563	-	Target "Motif:SIRE3_TC" 503 920
13	868296	868464	-	Target "Motif:SIRE3_TC" 590 777
13	868410	868631	-	Target "Motif:SIRE" 94 312
13	870171	870189	-	Target "Motif:SIRE" 73 90
13	870189	870443	-	Target "Motif:SIRE3_TC" 670 920
13	870396	870613	-	Target "Motif:SIRE" 98 313
13	882013	882475	-	Target "Motif:SIRE3_TC" 503 920
13	884209	884376	-	Target "Motif:SIRE3_TC" 590 777
13	884323	884543	-	Target "Motif:SIRE" 94 312
13	886081	886099	-	Target "Motif:SIRE" 73 90
13	886099	886354	-	Target "Motif:SIRE3_TC" 669 920
13	886306	886525	-	Target "Motif:SIRE" 98 313
14	257770	258213	-	Target "Motif:SIRE3_TC" 510 776
14	258007	258215	-	Target "Motif:SIRE" 98 315
14	264939	265442	+	Target "Motif:SIRE3_TC" 507 920
14	265442	265609	+	Target "Motif:SIRE" 106 261
14	265618	265809	+	Target "Motif:SIRE" 98 276
14	877922	878352	-	Target "Motif:SIRE3_TC" 511 920
14	904554	904992	-	Target "Motif:SIRE3_TC" 524 920
15	23318	23367	-	Target "Motif:SIRE" 95 144
15	31120	31189	-	Target "Motif:SIRE" 37 102
15	267712	267947	-	Target "Motif:SIRE3_TC" 728 920
15	283990	284039	+	Target "Motif:SIRE" 95 151
15	301856	302324	+	Target "Motif:SIRE3_TC" 393 781
15	305096	305570	+	Target "Motif:SIRE3_TC" 474 920
15	390447	390489	-	Target "Motif:SIRE" 102 150
15	408231	408560	-	Target "Motif:SIRE3_TC" 492 771
15	412853	413204	-	Target "Motif:SIRE3_TC" 475 771
15	417451	417783	-	Target "Motif:SIRE3_TC" 492 771
15	422036	422364	-	Target "Motif:SIRE3_TC" 492 771
15	426619	426969	-	Target "Motif:SIRE3_TC" 475 771
15	433289	433700	-	Target "Motif:SIRE3_TC" 514 913

15	633140	633474	-	Target "Motif:SIRE3_TC" 510 788
15	636101	636452	-	Target "Motif:SIRE3_TC" 504 790
15	647389	647729	-	Target "Motif:SIRE3_TC" 504 788
15	708522	708859	-	Target "Motif:SIRE3_TC" 504 788
15	736705	737140	-	Target "Motif:SIRE3_TC" 423 788
15	739788	740122	-	Target "Motif:SIRE3_TC" 510 788
15	742771	743107	-	Target "Motif:SIRE3_TC" 510 788
15	745757	746093	-	Target "Motif:SIRE3_TC" 510 788
15	748741	749075	-	Target "Motif:SIRE3_TC" 510 788
15	779668	779719	+	Target "Motif:SIRE" 95 148
15	783762	784037	+	Target "Motif:SIRE3_TC" 511 759
15	889262	889317	-	Target "Motif:SIRE" 54 108
15	889399	889730	-	Target "Motif:SIRE3_TC" 504 770
15	949794	949812	-	Target "Motif:SIRE" 73 90
15	949812	950064	-	Target "Motif:SIRE3_TC" 672 920
15	950017	950232	-	Target "Motif:SIRE" 98 313
16	969	1016	+	Target "Motif:SIRE" 95 145
16	327346	327784	-	Target "Motif:SIRE3_TC" 504 919
16	335318	335771	+	Target "Motif:SIRE3_TC" 257 920
16	500120	500325	+	Target "Motif:SIRE3_TC" 728 920
16	567768	567817	-	Target "Motif:SIRE" 95 145
16	578559	578608	+	Target "Motif:SIRE" 95 144
16	667669	668089	+	Target "Motif:SIRE3_TC" 511 920
16	669831	670001	+	Target "Motif:SIRE3_TC" 2911 3081
16	674604	675087	-	Target "Motif:SIRE3_TC" 480 837
16	679961	680342	-	Target "Motif:SIRE3_TC" 511 837
16	710178	710660	-	Target "Motif:SIRE3_TC" 480 837
16	725667	725878	-	Target "Motif:SIRE" 79 312
16	747160	747290	-	Target "Motif:SIRE" 100 223
16	747290	747312	-	Target "Motif:SIRE3_TC" 488 628
16	780280	780740	-	Target "Motif:SIRE3_TC" 504 911
16	850882	851322	-	Target "Motif:SIRE3_TC" 504 920
16	972811	973311	-	Target "Motif:SIRE3_TC" 477 917
16	989996	990045	-	Target "Motif:SIRE" 95 145
16	995686	996199	-	Target "Motif:SIRE3_TC" 479 771
16	1119547	1119731	-	Target "Motif:SIRE" 93 281
16	1130707	1130810	-	Target "Motif:SIRE" 101 202
16	1154102	1154595	+	Target "Motif:SIRE3_TC" 257 920
16	1169227	1169586	+	Target "Motif:SIRE3_TC" 561 919
16	1387711	1388164	+	Target "Motif:SIRE3_TC" 499 920
16	1391277	1391761	+	Target "Motif:SIRE3_TC" 257 912
18	227172	227220	-	Target "Motif:SIRE" 95 148
18	280540	280990	-	Target "Motif:SIRE3_TC" 507 920
18	477703	478173	+	Target "Motif:SIRE3_TC" 315 920
18	571046	571242	+	Target "Motif:SIRE" 91 291
18	579958	580185	-	Target "Motif:SIRE3_TC" 601 793

18	580255	580443	+	Target "Motif:SIRE" 101 291
18	588205	588415	-	Target "Motif:SIRE3_TC" 637 833
18	597405	597633	-	Target "Motif:SIRE3_TC" 601 793
18	603562	603790	-	Target "Motif:SIRE3_TC" 601 793
18	612885	613089	-	Target "Motif:SIRE3_TC" 601 771
18	613153	613405	+	Target "Motif:SIRE3_TC" 505 750
19	29853	30196	-	Target "Motif:SIRE3_TC" 510 838
19	127083	127424	-	Target "Motif:SIRE3_TC" 510 779
19	326997	327463	+	Target "Motif:SIRE3_TC" 339 829
19	399092	399153	+	Target "Motif:SIRE" 98 139
19	403772	404236	+	Target "Motif:SIRE3_TC" 494 919
19	442001	442413	+	Target "Motif:SIRE3_TC" 512 908
19	475586	476030	+	Target "Motif:SIRE3_TC" 503 920
19	515991	516399	+	Target "Motif:SIRE3_TC" 511 908
19	544285	544728	-	Target "Motif:SIRE3_TC" 504 920
2	15362	15776	-	Target "Motif:SIRE3_TC" 511 913
2	18586	18638	-	Target "Motif:SIRE3_TC" 505 770
2	18638	18855	-	Target "Motif:SIRE" 95 315
2	114925	115381	+	Target "Motif:SIRE3_TC" 390 824
2	136967	137254	+	Target "Motif:SIRE3_TC" 400 920
2	218239	218436	+	Target "Motif:SIRE3_TC" 593 777
2	231536	231736	+	Target "Motif:SIRE" 103 307
2	347913	348239	+	Target "Motif:SIRE3_TC" 513 828
2	438346	438805	+	Target "Motif:SIRE3_TC" 482 920
2	476022	476230	+	Target "Motif:SIRE" 94 296
2	627954	628387	+	Target "Motif:SIRE3_TC" 511 920
2	641576	641924	+	Target "Motif:SIRE3_TC" 510 782
2	652761	653106	-	Target "Motif:SIRE3_TC" 510 838
2	907481	907521	-	Target "Motif:SIRE" 101 147
2	911412	911769	-	Target "Motif:SIRE3_TC" 514 838
2	957732	958204	+	Target "Motif:SIRE3_TC" 339 920
2	995546	995767	+	Target "Motif:SIRE" 95 315
2	995767	995910	+	Target "Motif:SIRE3_TC" 505 782
2	998038	998497	+	Target "Motif:SIRE3_TC" 481 920
2	1028884	1029422	+	Target "Motif:SIRE7_TC" 271 920
2	1035402	1035844	+	Target "Motif:SIRE3_TC" 504 911
2	1039889	1039961	+	Target "Motif:SIRE" 34 102
2	1098009	1098216	+	Target "Motif:SIRE" 95 297
2	1128682	1128869	+	Target "Motif:SIRE" 94 281
2	1433591	1433633	-	Target "Motif:SIRE" 102 146
2	1643791	1644233	+	Target "Motif:SIRE3_TC" 499 911
2	1718851	1719284	+	Target "Motif:SIRE3_TC" 511 918
2	1728654	1728704	+	Target "Motif:SIRE" 95 144
2	1733017	1733199	+	Target "Motif:SIRE_TC" 485 668
2	1756659	1757098	+	Target "Motif:SIRE3_TC" 511 918
2	1764012	1764184	+	Target "Motif:SIRE3_TC" 595 774

2	1764202	1764348	+	Target "Motif:SIRE3_TC" 784 918
2	1781173	1781611	+	Target "Motif:SIRE3_TC" 511 918
20	194694	194874	+	Target "Motif:SIRE" 94 278
20	194911	195055	+	Target "Motif:SIRE7_TC" 532 682
20	236629	236812	+	Target "Motif:SIRE" 94 278
20	236847	236991	+	Target "Motif:SIRE7_TC" 532 682
20	336275	336737	-	Target "Motif:SIRE3_TC" 492 920
20	339197	339661	-	Target "Motif:SIRE3_TC" 492 920
20	341149	341611	-	Target "Motif:SIRE3_TC" 492 920
20	346018	346478	-	Target "Motif:SIRE3_TC" 494 920
20	359329	359687	+	Target "Motif:SIRE3_TC" 596 920
20	360366	360836	+	Target "Motif:SIRE3_TC" 393 781
20	505263	505443	+	Target "Motif:SIRE" 94 278
20	505482	505626	+	Target "Motif:SIRE7_TC" 532 682
20	518488	518666	+	Target "Motif:SIRE" 94 278
20	518668	518846	+	Target "Motif:SIRE7_TC" 532 674
20	726065	726272	+	Target "Motif:SIRE" 94 297
21	10132	10470	+	Target "Motif:SIRE3_TC" 505 828
21	102625	102973	-	Target "Motif:SIRE3_TC" 504 832
21	109455	109901	-	Target "Motif:SIRE3_TC" 504 920
21	128151	128200	-	Target "Motif:SIRE" 95 144
21	139481	139960	-	Target "Motif:SIRE3_TC" 486 920
21	140256	140499	-	Target "Motif:SIRE3_TC" 260 920
21	140499	140712	-	Target "Motif:SIRE" 98 315
21	145941	145990	+	Target "Motif:SIRE" 95 145
21	165280	165618	-	Target "Motif:SIRE3_TC" 503 788
21	301538	301609	-	Target "Motif:SIRE7_TC" 607 682
21	301689	301828	-	Target "Motif:SIRE3_TC" 520 780
21	301828	302053	-	Target "Motif:SIRE" 101 315
21	446300	446439	-	Target "Motif:SIRE3_TC" 520 780
21	446439	446664	-	Target "Motif:SIRE" 101 315
21	499810	500277	+	Target "Motif:SIRE3_TC" 489 920
21	702392	702702	-	Target "Motif:SIRE3_TC" 511 771
22	4761	4811	+	Target "Motif:SIRE" 94 143
22	13127	13170	+	Target "Motif:SIRE3_TC" 463 673
22	13170	13351	+	Target "Motif:SIRE" 94 270
23	515710	516140	-	Target "Motif:SIRE3_TC" 508 920
23	688557	688606	+	Target "Motif:SIRE" 95 145
23	696377	696598	+	Target "Motif:SIRE" 101 313
24	76166	76210	-	Target "Motif:SIRE" 100 144
24	109513	109850	-	Target "Motif:SIRE3_TC" 510 771
24	325025	325362	+	Target "Motif:SIRE3_TC" 510 771
24	387371	387573	+	Target "Motif:SIRE3_TC" 597 771
24	465102	465436	-	Target "Motif:SIRE3_TC" 510 771
24	600789	601223	+	Target "Motif:SIRE3_TC" 505 911
24	678916	679125	+	Target "Motif:SIRE" 94 297

24	684209	684421	+	Target "Motif:SIRE" 95 315
24	684421	684552	+	Target "Motif:SIRE3_TC" 505 776
24	691160	691232	+	Target "Motif:SIRE" 34 102
25	2859	2913	-	Target "Motif:SIRE" 91 142
25	191912	192404	+	Target "Motif:SIRE3_TC" 257 920
25	231438	231585	-	Target "Motif:SIRE" 101 241
25	678278	678422	-	Target "Motif:SIRE" 103 241
25	680346	680493	+	Target "Motif:SIRE" 101 241
26	73092	73540	+	Target "Motif:SIRE3_TC" 290 910
26	152130	152173	+	Target "Motif:SIRE" 101 144
26	235535	235554	-	Target "Motif:SIRE" 102 125
26	235554	235987	-	Target "Motif:SIRE3_TC" 508 920
26	323982	324225	-	Target "Motif:SIRE3_TC" 516 768
26	550955	551134	+	Target "Motif:SIRE" 101 281
26	556257	556476	+	Target "Motif:SIRE" 95 315
26	556476	556620	+	Target "Motif:SIRE3_TC" 505 782
26	563478	563549	+	Target "Motif:SIRE" 34 102
26	627565	628107	+	Target "Motif:SIRE7_TC" 265 920
26	669268	669493	+	Target "Motif:SIRE" 95 315
26	669301	669790	+	Target "Motif:SIRE7_TC" 250 741
27	1311	1502	-	Target "Motif:SIRE3_TC" 646 831
27	1433	1573	-	Target "Motif:SIRE" 170 313
27	1582	1753	+	Target "Motif:SIRE" 105 281
27	10538	10736	-	Target "Motif:SIRE3_TC" 646 831
27	10667	10810	-	Target "Motif:SIRE" 168 313
27	10816	10992	+	Target "Motif:SIRE" 103 281
27	158369	158824	-	Target "Motif:SIRE3_TC" 511 920
27	171164	171616	-	Target "Motif:SIRE3_TC" 511 920
27	178461	178910	-	Target "Motif:SIRE3_TC" 511 920
27	183515	183844	-	Target "Motif:SIRE3_TC" 492 771
27	188060	188390	-	Target "Motif:SIRE3_TC" 492 771
27	194679	195178	-	Target "Motif:SIRE3_TC" 511 913
27	203153	203605	-	Target "Motif:SIRE3_TC" 511 920
27	239553	239983	-	Target "Motif:SIRE3_TC" 511 920
27	408006	408298	-	Target "Motif:SIRE3_TC" 669 920
27	411639	411923	-	Target "Motif:SIRE3_TC" 669 920
27	415279	415564	-	Target "Motif:SIRE3_TC" 669 920
27	418919	419204	-	Target "Motif:SIRE3_TC" 669 920
27	422551	422841	-	Target "Motif:SIRE3_TC" 669 920
27	430794	431234	+	Target "Motif:SIRE3_TC" 341 909
27	484089	484314	+	Target "Motif:SIRE" 94 313
27	484314	484525	+	Target "Motif:SIRE3_TC" 357 898
27	509905	510104	+	Target "Motif:SIRE" 101 295
27	536804	537008	+	Target "Motif:SIRE" 101 300
27	578348	578781	+	Target "Motif:SIRE3_TC" 502 920
28	226507	226726	+	Target "Motif:SIRE" 110 313

28	226595	226986	+	Target "Motif:SIRE3_TC" 319 920
28	279577	279797	+	Target "Motif:SIRE" 110 313
28	279666	280057	+	Target "Motif:SIRE3_TC" 319 920
28	287027	287537	+	Target "Motif:SIRE3_TC" 319 920
28	312823	312997	+	Target "Motif:SIRE" 99 276
28	386140	386317	+	Target "Motif:SIRE" 95 277
28	427276	427474	+	Target "Motif:SIRE" 98 294
29	122413	122912	-	Target "Motif:SIRE3_TC" 485 782
29	122941	122994	-	Target "Motif:SIRE3_TC" 468 511
29	136143	136578	+	Target "Motif:SIRE3_TC" 504 920
29	146730	147164	+	Target "Motif:SIRE3_TC" 504 920
29	297573	297785	+	Target "Motif:SIRE" 102 315
29	297785	298045	+	Target "Motif:SIRE3_TC" 468 778
29	302572	302759	+	Target "Motif:SIRE" 98 281
29	320132	320319	-	Target "Motif:SIRE" 98 281
29	337401	337849	+	Target "Motif:SIRE3_TC" 504 920
29	395194	395405	+	Target "Motif:SIRE" 94 297
29	497610	497666	-	Target "Motif:SIRE3_TC" 568 771
29	497666	497881	-	Target "Motif:SIRE" 98 313
29	497881	497901	-	Target "Motif:SIRE3_TC" 487 567
29	502813	503096	-	Target "Motif:SIRE3_TC" 561 778
29	507831	507870	-	Target "Motif:SIRE" 98 140
29	509449	509499	+	Target "Motif:SIRE" 94 144
29	514255	514669	+	Target "Motif:SIRE3_TC" 280 920
29	516507	517008	+	Target "Motif:SIRE3_TC" 283 920
3	991	1433	-	Target "Motif:SIRE3_TC" 481 920
3	3426	3615	-	Target "Motif:SIRE_TC" 485 667
3	3590	3694	-	Target "Motif:SIRE3_TC" 507 774
3	3694	3905	-	Target "Motif:SIRE" 98 315
3	9017	9234	-	Target "Motif:SIRE" 94 304
3	34561	34633	-	Target "Motif:SIRE" 34 102
3	39019	39483	-	Target "Motif:SIRE3_TC" 481 920
3	41629	41769	-	Target "Motif:SIRE3_TC" 505 777
3	41769	41992	-	Target "Motif:SIRE" 95 315
3	47978	48473	-	Target "Motif:SIRE7_TC" 250 741
3	48279	48507	-	Target "Motif:SIRE" 95 315
3	52585	52755	-	Target "Motif:SIRE" 94 268
3	104060	104493	-	Target "Motif:SIRE3_TC" 502 920
3	109718	109945	-	Target "Motif:SIRE3_TC" 506 899
3	109945	110165	-	Target "Motif:SIRE" 94 313
3	143755	144209	-	Target "Motif:SIRE3_TC" 510 920
3	246770	247257	-	Target "Motif:SIRE3_TC" 303 899
3	251674	251743	-	Target "Motif:SIRE" 53 121
3	251697	252181	-	Target "Motif:SIRE3_TC" 370 899
3	292143	292376	-	Target "Motif:SIRE3_TC" 703 912
3	296396	296842	-	Target "Motif:SIRE3_TC" 504 920

3	611076	611578	+	Target "Motif:SIRE3_TC" 507 920
3	645992	646181	-	Target "Motif:SIRE" 98 276
3	646190	646357	-	Target "Motif:SIRE" 106 261
3	646357	646855	-	Target "Motif:SIRE3_TC" 507 920
3	669785	670135	-	Target "Motif:SIRE3_TC" 510 838
3	688336	688526	-	Target "Motif:SIRE" 98 276
3	688535	688700	-	Target "Motif:SIRE" 106 261
3	688700	689205	-	Target "Motif:SIRE3_TC" 507 920
3	957961	958433	-	Target "Motif:SIRE3_TC" 474 920
3	961206	961675	-	Target "Motif:SIRE3_TC" 486 781
3	1082110	1082627	+	Target "Motif:SIRE3_TC" 482 771
3	1082562	1082894	+	Target "Motif:SIRE" 38 315
3	1082838	1083101	+	Target "Motif:SIRE3_TC" 664 903
3	1092993	1093192	+	Target "Motif:SIRE3_TC" 482 680
3	1093230	1093516	+	Target "Motif:SIRE3_TC" 603 770
3	1093451	1093781	+	Target "Motif:SIRE" 38 315
3	1093716	1094037	+	Target "Motif:SIRE3_TC" 391 771
3	1235721	1235999	+	Target "Motif:SIRE3_TC" 671 771
3	1317774	1317818	+	Target "Motif:SIRE3_TC" 507 550
3	1317779	1317998	+	Target "Motif:SIRE" 102 281
3	1317870	1318119	+	Target "Motif:SIRE3_TC" 562 769
3	1324351	1324693	+	Target "Motif:SIRE3_TC" 393 907
3	1363890	1364055	+	Target "Motif:SIRE3_TC" 660 822
3	1454612	1455090	+	Target "Motif:SIRE3_TC" 464 920
3	1629465	1629513	+	Target "Motif:SIRE" 95 143
30	146674	147046	-	Target "Motif:SIRE3_TC" 510 782
30	258969	259341	+	Target "Motif:SIRE3_TC" 510 782
31	323850	324334	-	Target "Motif:SIRE3_TC" 511 837
31	392189	392670	+	Target "Motif:SIRE3_TC" 480 836
31	421720	421996	+	Target "Motif:SIRE3_TC" 429 920
31	446089	446132	+	Target "Motif:SIRE3_TC" 464 552
31	446132	446349	+	Target "Motif:SIRE" 98 310
31	446349	446482	+	Target "Motif:SIRE3_TC" 553 836
31	453830	454198	+	Target "Motif:SIRE3_TC" 494 837
31	487519	487984	+	Target "Motif:SIRE3_TC" 492 920
32	385565	385755	-	Target "Motif:SIRE" 94 281
32	407769	408211	-	Target "Motif:SIRE3_TC" 511 918
32	441036	441218	-	Target "Motif:SIRE_TC" 485 668
32	449841	450284	-	Target "Motif:SIRE3_TC" 511 918
32	548355	548792	+	Target "Motif:SIRE3_TC" 511 919
32	608794	609044	-	Target "Motif:SIRE7_TC" 444 708
32	609096	609508	-	Target "Motif:SIRE3_TC" 504 920
32	613700	613748	-	Target "Motif:SIRE" 98 143
32	710044	710254	+	Target "Motif:SIRE" 101 314
32	710254	710311	+	Target "Motif:SIRE3_TC" 511 771
32	710325	710875	+	Target "Motif:SIRE_TC" 469 920

32	713068	713282	+	Target "Motif:SIRE" 95 314
32	713282	713339	+	Target "Motif:SIRE3_TC" 530 771
32	713353	713904	+	Target "Motif:SIRE_TC" 469 920
32	719472	719688	+	Target "Motif:SIRE" 95 314
32	719688	719745	+	Target "Motif:SIRE3_TC" 530 771
32	719759	720308	+	Target "Motif:SIRE_TC" 469 920
32	724196	724412	+	Target "Motif:SIRE" 95 314
32	724412	724469	+	Target "Motif:SIRE3_TC" 530 771
32	724482	725033	+	Target "Motif:SIRE_TC" 469 920
32	728923	729139	+	Target "Motif:SIRE" 95 314
32	729139	729760	+	Target "Motif:SIRE_TC" 476 920
32	733651	733867	+	Target "Motif:SIRE" 95 314
32	733867	734489	+	Target "Motif:SIRE_TC" 476 920
32	736683	736899	+	Target "Motif:SIRE" 95 314
32	736899	737519	+	Target "Motif:SIRE_TC" 476 920
32	741247	741463	+	Target "Motif:SIRE" 95 314
32	741463	742084	+	Target "Motif:SIRE_TC" 476 920
32	744281	744497	+	Target "Motif:SIRE" 95 314
32	744497	745118	+	Target "Motif:SIRE_TC" 476 920
32	748953	748986	+	Target "Motif:SIRE_TC" 469 491
32	748986	749201	+	Target "Motif:SIRE" 95 314
32	749201	749822	+	Target "Motif:SIRE_TC" 492 920
32	752009	752225	+	Target "Motif:SIRE" 95 314
32	752225	752844	+	Target "Motif:SIRE_TC" 476 920
32	757261	757479	+	Target "Motif:SIRE" 92 314
32	757262	757530	+	Target "Motif:SIRE3_TC" 502 765
32	757555	757745	+	Target "Motif:SIRE3_TC" 473 667
32	757715	757751	+	Target "Motif:SIRE7_TC" 646 681
32	761817	762153	+	Target "Motif:SIRE3_TC" 647 920
32	813156	813343	+	Target "Motif:SIRE" 93 281
32	905801	906248	+	Target "Motif:SIRE3_TC" 494 920
32	992763	993055	-	Target "Motif:SIRE3_TC" 511 771
32	992848	993061	-	Target "Motif:SIRE" 95 315
32	999217	999491	-	Target "Motif:SIRE3_TC" 513 772
33	208317	208534	+	Target "Motif:SIRE" 98 315
33	208320	208759	+	Target "Motif:SIRE3_TC" 511 920
33	213143	213192	-	Target "Motif:SIRE" 95 141
33	264795	264841	+	Target "Motif:SIRE" 95 139
33	426642	427137	-	Target "Motif:SIRE3_TC" 464 770
33	436559	436960	-	Target "Motif:SIRE3_TC" 556 911
33	436989	437036	-	Target "Motif:SIRE3_TC" 509 555
33	475500	475568	+	Target "Motif:SIRE" 37 102
34	172219	172405	-	Target "Motif:SIRE" 95 281
34	376135	376244	+	Target "Motif:SIRE" 98 206
34	376138	376518	+	Target "Motif:SIRE3_TC" 511 837
34	415504	415682	+	Target "Motif:SIRE" 95 281

34	422677	423154	+	Target "Motif:SIRE3_TC" 290 920
34	487088	487318	+	Target "Motif:SIRE" 94 313
34	487318	487534	+	Target "Motif:SIRE3_TC" 506 827
35	57880	58228	+	Target "Motif:SIRE3_TC" 510 838
35	77509	77852	+	Target "Motif:SIRE3_TC" 510 838
35	215413	215752	-	Target "Motif:SIRE3_TC" 597 919
35	362032	362495	-	Target "Motif:SIRE3_TC" 504 920
35	381196	381208	-	Target "Motif:SIRE" 106 119
35	381353	381425	-	Target "Motif:SIRE" 35 105
35	386008	386050	-	Target "Motif:SIRE" 102 147
35	394704	394746	-	Target "Motif:SIRE" 102 147
35	397730	397772	+	Target "Motif:SIRE" 102 146
35	510103	510149	-	Target "Motif:SIRE" 98 146
35	513125	513171	+	Target "Motif:SIRE" 98 146
35	517787	517859	+	Target "Motif:SIRE" 34 104
35	517948	517960	+	Target "Motif:SIRE" 105 118
36	40435	40646	-	Target "Motif:SIRE" 95 313
36	65873	66154	-	Target "Motif:SIRE3_TC" 671 903
36	90223	90506	-	Target "Motif:SIRE3_TC" 671 903
36	299441	299461	+	Target "Motif:SIRE3_TC" 487 556
36	299461	299675	+	Target "Motif:SIRE" 98 313
36	299675	299732	+	Target "Motif:SIRE3_TC" 557 771
36	309474	309961	-	Target "Motif:SIRE3_TC" 511 827
36	316076	316188	-	Target "Motif:SIRE" 165 281
36	316216	316279	-	Target "Motif:SIRE" 99 164
36	412484	412996	+	Target "Motif:SIRE3_TC" 504 920
37	12153	12662	+	Target "Motif:SIRE3_TC" 290 920
37	33374	33431	-	Target "Motif:SIRE3_TC" 567 771
37	33431	33646	-	Target "Motif:SIRE" 98 313
37	33646	33666	-	Target "Motif:SIRE3_TC" 487 566
37	42828	42867	-	Target "Motif:SIRE" 98 140
37	44446	44495	+	Target "Motif:SIRE" 94 144
37	75397	75694	-	Target "Motif:SIRE3_TC" 647 915
37	75774	75947	+	Target "Motif:SIRE" 101 282
37	92285	92577	-	Target "Motif:SIRE3_TC" 646 916
37	92508	92660	-	Target "Motif:SIRE" 163 313
37	92655	92827	+	Target "Motif:SIRE" 101 282
37	124128	124424	-	Target "Motif:SIRE3_TC" 646 916
37	124354	124469	-	Target "Motif:SIRE" 198 313
37	124502	124672	+	Target "Motif:SIRE" 101 282
37	151696	151764	+	Target "Motif:SIRE" 98 163
37	191869	192039	+	Target "Motif:SIRE" 99 274
37	204241	204529	-	Target "Motif:SIRE3_TC" 646 916
37	204462	204612	-	Target "Motif:SIRE" 163 313
37	204607	204780	+	Target "Motif:SIRE" 101 282
37	216956	217247	-	Target "Motif:SIRE3_TC" 646 916

37	217182	217293	-	Target "Motif:SIRE" 197 309
37	260790	260963	-	Target "Motif:SIRE" 101 282
37	260980	261107	+	Target "Motif:SIRE" 181 309
37	261042	261335	+	Target "Motif:SIRE3_TC" 646 916
37	278322	278509	-	Target "Motif:SIRE" 101 295
37	311131	311495	-	Target "Motif:SIRE3_TC" 511 782
37	328454	328629	+	Target "Motif:SIRE" 104 281
37	341617	341976	-	Target "Motif:SIRE3_TC" 504 782
37	353969	354428	+	Target "Motif:SIRE3_TC" 260 920
37	358862	359224	+	Target "Motif:SIRE3_TC" 504 782
37	361477	361528	+	Target "Motif:SIRE" 95 148
37	503769	504247	+	Target "Motif:SIRE3_TC" 251 911
38	419945	420120	+	Target "Motif:SIRE" 107 281
39	93176	93515	-	Target "Motif:SIRE3_TC" 505 828
39	96405	96817	-	Target "Motif:SIRE3_TC" 505 903
39	99707	100120	-	Target "Motif:SIRE3_TC" 505 903
39	103006	103419	-	Target "Motif:SIRE3_TC" 505 903
39	106301	106714	-	Target "Motif:SIRE3_TC" 505 903
39	365085	365422	+	Target "Motif:SIRE3_TC" 505 828
39	368379	368715	+	Target "Motif:SIRE3_TC" 505 828
4	60503	60930	+	Target "Motif:SIRE3_TC" 483 870
4	94753	95271	+	Target "Motif:SIRE3_TC" 390 919
4	101028	101494	+	Target "Motif:SIRE7_TC" 231 694
4	211431	211717	+	Target "Motif:SIRE3_TC" 504 774
4	213471	213799	+	Target "Motif:SIRE3_TC" 504 774
4	215515	215843	+	Target "Motif:SIRE3_TC" 504 774
4	217547	217894	+	Target "Motif:SIRE3_TC" 504 776
4	242035	242363	+	Target "Motif:SIRE3_TC" 504 774
4	273368	273695	+	Target "Motif:SIRE3_TC" 504 774
4	277013	277340	+	Target "Motif:SIRE3_TC" 504 774
4	292255	292540	+	Target "Motif:SIRE3_TC" 504 774
4	295873	296320	+	Target "Motif:SIRE3_TC" 504 778
4	308375	308703	+	Target "Motif:SIRE3_TC" 504 774
4	316748	317195	+	Target "Motif:SIRE3_TC" 504 778
4	328418	328737	+	Target "Motif:SIRE3_TC" 504 774
4	330432	330880	+	Target "Motif:SIRE3_TC" 504 774
4	332477	332920	+	Target "Motif:SIRE3_TC" 504 774
4	334488	334929	+	Target "Motif:SIRE3_TC" 504 774
4	357511	357776	+	Target "Motif:SIRE3_TC" 518 774
4	359518	359843	+	Target "Motif:SIRE3_TC" 504 774
4	361563	362001	+	Target "Motif:SIRE3_TC" 515 774
4	363590	363914	+	Target "Motif:SIRE3_TC" 504 774
4	365601	366051	+	Target "Motif:SIRE3_TC" 504 774
4	367632	367957	+	Target "Motif:SIRE3_TC" 505 774
4	369665	369989	+	Target "Motif:SIRE3_TC" 505 774
4	371699	372024	+	Target "Motif:SIRE3_TC" 504 774

4	383754	384080	+	Target "Motif:SIRE3_TC" 504 774
4	385787	385987	+	Target "Motif:SIRE" 98 302
4	403580	404029	+	Target "Motif:SIRE3_TC" 504 774
4	405616	405943	+	Target "Motif:SIRE3_TC" 504 774
4	407650	407931	+	Target "Motif:SIRE3_TC" 504 774
4	409686	410136	+	Target "Motif:SIRE3_TC" 504 774
4	434632	434959	+	Target "Motif:SIRE3_TC" 504 774
4	436668	436996	+	Target "Motif:SIRE3_TC" 504 774
4	438707	439035	+	Target "Motif:SIRE3_TC" 504 774
4	440750	441078	+	Target "Motif:SIRE3_TC" 504 774
4	442792	443120	+	Target "Motif:SIRE3_TC" 504 774
4	444835	445163	+	Target "Motif:SIRE3_TC" 504 774
4	446876	447204	+	Target "Motif:SIRE3_TC" 504 774
4	448917	449244	+	Target "Motif:SIRE3_TC" 504 774
4	450958	451408	+	Target "Motif:SIRE3_TC" 504 774
4	452990	453316	+	Target "Motif:SIRE3_TC" 504 774
4	455015	455299	+	Target "Motif:SIRE3_TC" 504 774
4	463089	463539	+	Target "Motif:SIRE3_TC" 504 774
4	493696	494146	+	Target "Motif:SIRE3_TC" 504 774
4	562605	562726	+	Target "Motif:SIRE" 97 220
4	572534	572747	+	Target "Motif:SIRE" 97 315
4	572970	573012	+	Target "Motif:SIRE" 101 146
4	577639	578129	+	Target "Motif:SIRE3_TC" 480 836
4	594250	594427	+	Target "Motif:SIRE" 98 281
4	627733	628207	+	Target "Motif:SIRE3_TC" 492 920
4	653561	653836	+	Target "Motif:SIRE3_TC" 667 920
4	726019	726522	+	Target "Motif:SIRE3_TC" 480 779
4	738284	738788	+	Target "Motif:SIRE3_TC" 480 771
4	762204	762708	-	Target "Motif:SIRE3_TC" 480 771
4	780131	780184	+	Target "Motif:SIRE" 91 146
4	808867	809375	+	Target "Motif:SIRE3_TC" 480 771
4	816365	816726	+	Target "Motif:SIRE3_TC" 491 779
4	837681	838043	-	Target "Motif:SIRE3_TC" 491 779
4	851609	852117	-	Target "Motif:SIRE3_TC" 480 771
4	859137	859643	-	Target "Motif:SIRE3_TC" 480 771
4	864722	865077	-	Target "Motif:SIRE3_TC" 495 779
4	872112	872171	-	Target "Motif:SIRE" 45 101
4	872171	872598	-	Target "Motif:SIRE3_TC" 480 771
4	882617	882979	-	Target "Motif:SIRE3_TC" 491 779
4	889972	890474	-	Target "Motif:SIRE3_TC" 480 771
4	898707	899067	-	Target "Motif:SIRE3_TC" 491 779
4	906088	906602	-	Target "Motif:SIRE3_TC" 480 774
4	920529	920960	-	Target "Motif:SIRE3_TC" 511 918
4	920761	920963	-	Target "Motif:SIRE" 98 312
4	942867	943309	-	Target "Motif:SIRE3_TC" 511 918
4	978520	978961	-	Target "Motif:SIRE3_TC" 511 918

4	995398	995837	-	Target "Motif:SIRE3_TC" 511 918
4	1037780	1038223	-	Target "Motif:SIRE3_TC" 511 918
4	1093491	1093826	+	Target "Motif:SIRE3_TC" 504 828
4	1244863	1245196	-	Target "Motif:SIRE3_TC" 504 828
4	1254059	1254396	-	Target "Motif:SIRE3_TC" 504 828
4	1280991	1281329	-	Target "Motif:SIRE3_TC" 503 838
4	1283081	1283553	-	Target "Motif:SIRE3_TC" 468 920
4	1289182	1289635	-	Target "Motif:SIRE3_TC" 494 920
4	1392161	1392625	-	Target "Motif:SIRE3_TC" 491 920
4	1433865	1434202	+	Target "Motif:SIRE3_TC" 505 828
4	1437187	1437525	+	Target "Motif:SIRE3_TC" 505 828
4	1440517	1440855	+	Target "Motif:SIRE3_TC" 505 828
4	1443849	1444187	+	Target "Motif:SIRE3_TC" 505 828
4	1447174	1447512	+	Target "Motif:SIRE3_TC" 505 828
4	1450500	1450837	+	Target "Motif:SIRE3_TC" 505 828
4	1453828	1454166	+	Target "Motif:SIRE3_TC" 505 828
4	1467933	1467982	+	Target "Motif:SIRE" 95 144
4	1529620	1529950	+	Target "Motif:SIRE3_TC" 502 822
40	28	299	-	Target "Motif:SIRE3_TC" 504 777
40	2293	2617	-	Target "Motif:SIRE3_TC" 504 830
40	4525	4941	-	Target "Motif:SIRE3_TC" 504 919
40	6933	7256	-	Target "Motif:SIRE3_TC" 504 830
40	15362	15771	-	Target "Motif:SIRE3_TC" 504 919
40	17664	18083	-	Target "Motif:SIRE3_TC" 504 919
40	20000	20420	-	Target "Motif:SIRE3_TC" 504 919
40	22324	22740	-	Target "Motif:SIRE3_TC" 504 919
40	24643	24936	-	Target "Motif:SIRE3_TC" 637 919
40	24855	25000	-	Target "Motif:SIRE" 167 313
40	37796	38178	-	Target "Motif:SIRE3_TC" 577 920
40	38294	38402	-	Target "Motif:SIRE" 98 207
40	40277	40700	-	Target "Motif:SIRE3_TC" 504 919
40	42617	43037	-	Target "Motif:SIRE3_TC" 504 919
40	46179	46598	-	Target "Motif:SIRE3_TC" 504 919
40	48521	48941	-	Target "Motif:SIRE3_TC" 504 919
40	50860	51281	-	Target "Motif:SIRE3_TC" 504 919
40	53206	53627	-	Target "Motif:SIRE3_TC" 504 919
40	55545	55966	-	Target "Motif:SIRE3_TC" 504 919
40	57885	58307	-	Target "Motif:SIRE3_TC" 504 919
40	60167	60497	-	Target "Motif:SIRE3_TC" 504 830
40	62412	62832	-	Target "Motif:SIRE3_TC" 504 919
40	64745	65165	-	Target "Motif:SIRE3_TC" 504 919
40	67166	67495	-	Target "Motif:SIRE3_TC" 504 830
40	69402	69820	-	Target "Motif:SIRE3_TC" 504 919
40	74559	74979	-	Target "Motif:SIRE3_TC" 504 919
40	76896	77316	-	Target "Motif:SIRE3_TC" 504 919
40	85775	85928	+	Target "Motif:SIRE" 159 313
