# Supplementary material for: Repeat-Driven Generation of Antigenic Diversity in a Major Human Pathogen, Trypanosoma cruzi
Source: Front Cell Infect Microbiol. 2021 Mar 3;11:614665. doi: 10.3389/fcimb.2021.614665 (PMC7966520; doi:10.3389/fcimb.2021.614665)
Supplement: Supplementary file 5 [file DataSheet_5.pdf]

Supplementary table 4, part 2.

40	85849	86049	+	Target "Motif:SIRE3_TC" 637 830
40	87659	88080	+	Target "Motif:SIRE3_TC" 504 919
40	90002	90423	+	Target "Motif:SIRE3_TC" 504 919
40	92345	92766	+	Target "Motif:SIRE3_TC" 504 919
40	94687	95107	+	Target "Motif:SIRE3_TC" 504 919
40	97028	97450	+	Target "Motif:SIRE3_TC" 504 919
40	100228	100375	+	Target "Motif:SIRE" 159 313
40	100299	100494	+	Target "Motif:SIRE3_TC" 637 830
40	102488	102901	+	Target "Motif:SIRE3_TC" 504 919
40	104806	105221	+	Target "Motif:SIRE3_TC" 504 919
40	107128	107546	+	Target "Motif:SIRE3_TC" 504 919
40	109465	109886	+	Target "Motif:SIRE3_TC" 504 919
40	111809	112230	+	Target "Motif:SIRE3_TC" 504 919
40	114154	114575	+	Target "Motif:SIRE3_TC" 504 919
40	116496	116917	+	Target "Motif:SIRE3_TC" 504 919
40	118774	119191	+	Target "Motif:SIRE3_TC" 505 919
40	121111	121444	+	Target "Motif:SIRE3_TC" 504 830
40	125131	125551	+	Target "Motif:SIRE3_TC" 504 919
40	127468	127889	+	Target "Motif:SIRE3_TC" 504 919
40	129810	130231	+	Target "Motif:SIRE3_TC" 504 919
40	132154	132575	+	Target "Motif:SIRE3_TC" 504 919
40	134496	134916	+	Target "Motif:SIRE3_TC" 504 919
40	136835	137256	+	Target "Motif:SIRE3_TC" 504 919
40	139193	139426	+	Target "Motif:SIRE3_TC" 504 740
40	139426	139665	+	Target "Motif:SIRE3_TC" 691 919
40	143594	143867	+	Target "Motif:SIRE3_TC" 561 830
40	145848	146178	+	Target "Motif:SIRE3_TC" 504 830
40	148177	148595	+	Target "Motif:SIRE3_TC" 504 919
40	150476	150896	+	Target "Motif:SIRE3_TC" 504 919
40	152812	153233	+	Target "Motif:SIRE3_TC" 504 919
40	155151	155572	+	Target "Motif:SIRE3_TC" 504 919
40	157489	157908	+	Target "Motif:SIRE3_TC" 504 919
40	159822	160242	+	Target "Motif:SIRE3_TC" 504 919
40	162162	162583	+	Target "Motif:SIRE3_TC" 504 919
40	164505	164925	+	Target "Motif:SIRE3_TC" 504 919
40	166845	167266	+	Target "Motif:SIRE3_TC" 504 919
40	169189	169609	+	Target "Motif:SIRE3_TC" 504 919
40	171526	171946	+	Target "Motif:SIRE3_TC" 504 919
40	173807	173914	+	Target "Motif:SIRE" 98 207
40	174028	174403	+	Target "Motif:SIRE3_TC" 318 920
40	184795	185232	+	Target "Motif:SIRE3_TC" 504 919
40	187111	187529	+	Target "Motif:SIRE3_TC" 504 919
40	189380	189489	+	Target "Motif:SIRE" 98 207
40	189605	189954	+	Target "Motif:SIRE3_TC" 312 920
40	196618	196720	+	Target "Motif:SIRE" 98 201
40	196852	197219	+	Target "Motif:SIRE3_TC" 320 920

40	246540	246811	-	Target "Motif:SIRE3_TC" 685 919
40	251181	251227	-	Target "Motif:SIRE" 98 145
40	251224	251404	-	Target "Motif:SIRE" 95 281
40	253328	253748	-	Target "Motif:SIRE3_TC" 504 919
40	255671	256092	-	Target "Motif:SIRE3_TC" 504 919
40	258017	258438	-	Target "Motif:SIRE3_TC" 504 919
40	260362	260782	-	Target "Motif:SIRE3_TC" 504 919
40	262704	263121	-	Target "Motif:SIRE3_TC" 504 919
40	265026	265448	-	Target "Motif:SIRE3_TC" 504 919
40	267558	267674	-	Target "Motif:SIRE3_TC" 835 920
40	267701	268044	-	Target "Motif:SIRE3_TC" 504 834
40	271785	272234	-	Target "Motif:SIRE3_TC" 504 919
40	274150	274571	-	Target "Motif:SIRE3_TC" 504 919
40	276491	276910	-	Target "Motif:SIRE3_TC" 504 919
40	278832	279254	-	Target "Motif:SIRE3_TC" 504 919
40	280773	281062	-	Target "Motif:SIRE3_TC" 637 919
40	280982	281136	-	Target "Motif:SIRE" 159 313
40	286130	286551	-	Target "Motif:SIRE3_TC" 504 919
40	288473	288894	-	Target "Motif:SIRE3_TC" 504 919
40	290818	291237	-	Target "Motif:SIRE3_TC" 504 919
40	297963	298384	-	Target "Motif:SIRE3_TC" 504 919
40	300300	300720	-	Target "Motif:SIRE3_TC" 504 919
40	302637	303057	-	Target "Motif:SIRE3_TC" 504 919
40	304976	305400	-	Target "Motif:SIRE3_TC" 504 919
40	307317	307738	-	Target "Motif:SIRE3_TC" 504 919
40	317423	317841	-	Target "Motif:SIRE3_TC" 504 919
40	319757	320178	-	Target "Motif:SIRE3_TC" 504 919
40	322094	322516	-	Target "Motif:SIRE3_TC" 504 919
40	324431	324850	-	Target "Motif:SIRE3_TC" 504 919
40	326759	327180	-	Target "Motif:SIRE3_TC" 504 919
40	329097	329519	-	Target "Motif:SIRE3_TC" 504 919
40	332437	332857	-	Target "Motif:SIRE3_TC" 504 919
40	334772	335193	-	Target "Motif:SIRE3_TC" 504 919
40	336709	336998	-	Target "Motif:SIRE3_TC" 637 919
40	336918	337072	-	Target "Motif:SIRE" 159 313
40	345554	345974	+	Target "Motif:SIRE3_TC" 504 919
40	347892	348312	+	Target "Motif:SIRE3_TC" 504 919
40	350224	350553	+	Target "Motif:SIRE3_TC" 504 830
40	351988	352410	+	Target "Motif:SIRE3_TC" 504 919
40	354286	354703	+	Target "Motif:SIRE3_TC" 504 919
40	356593	357010	+	Target "Motif:SIRE3_TC" 504 916
40	358895	359311	+	Target "Motif:SIRE3_TC" 504 919
40	361214	361540	+	Target "Motif:SIRE3_TC" 504 830
40	377484	377907	+	Target "Motif:SIRE3_TC" 504 919
40	379826	380249	+	Target "Motif:SIRE3_TC" 504 919
40	381959	382381	+	Target "Motif:SIRE3_TC" 504 919

40	384300	384718	+	Target "Motif:SIRE3_TC" 504 919
40	386624	387041	+	Target "Motif:SIRE3_TC" 504 919
40	388953	389390	+	Target "Motif:SIRE3_TC" 504 830
40	391290	391707	+	Target "Motif:SIRE3_TC" 504 919
40	393625	394029	+	Target "Motif:SIRE3_TC" 504 919
40	395795	396217	+	Target "Motif:SIRE3_TC" 504 919
40	398131	398551	+	Target "Motif:SIRE3_TC" 504 919
40	400467	400889	+	Target "Motif:SIRE3_TC" 504 919
40	402769	402876	+	Target "Motif:SIRE" 98 207
40	402991	403368	+	Target "Motif:SIRE3_TC" 318 920
41	135274	135707	-	Target "Motif:SIRE3_TC" 505 911
41	188080	188112	+	Target "Motif:SIRE3_TC" 647 682
41	188145	188392	+	Target "Motif:SIRE3_TC" 683 919
41	277256	277323	+	Target "Motif:SIRE3_TC" 706 771
41	277467	277537	+	Target "Motif:SIRE" 35 101
42	30628	30976	-	Target "Motif:SIRE3_TC" 510 838
42	37059	37406	-	Target "Motif:SIRE3_TC" 510 838
42	102039	102496	-	Target "Motif:SIRE3_TC" 474 920
42	138919	139125	-	Target "Motif:SIRE" 95 297
42	145404	145609	-	Target "Motif:SIRE" 94 293
42	160559	160631	-	Target "Motif:SIRE" 33 102
42	168384	168840	-	Target "Motif:SIRE3_TC" 474 920
42	321704	322186	-	Target "Motif:SIRE3_TC" 471 920
42	323013	323496	-	Target "Motif:SIRE3_TC" 471 920
42	324321	324804	-	Target "Motif:SIRE3_TC" 471 920
42	325631	326114	-	Target "Motif:SIRE3_TC" 471 920
42	326944	327427	-	Target "Motif:SIRE3_TC" 471 920
42	328260	328742	-	Target "Motif:SIRE3_TC" 471 920
42	329565	330047	-	Target "Motif:SIRE3_TC" 471 920
42	330873	331314	-	Target "Motif:SIRE3_TC" 504 920
42	338802	338989	-	Target "Motif:SIRE3_TC" 645 832
42	388009	388218	-	Target "Motif:SIRE" 98 282
42	390608	390797	-	Target "Motif:SIRE3_TC" 645 834
42	405784	405859	-	Target "Motif:SIRE7_TC" 612 682
42	406063	406337	-	Target "Motif:SIRE3_TC" 502 771
42	447134	447473	+	Target "Motif:SIRE3_TC" 471 771
42	448730	449162	+	Target "Motif:SIRE3_TC" 504 920
42	449583	450043	+	Target "Motif:SIRE3_TC" 469 836
42	461447	461777	+	Target "Motif:SIRE3_TC" 471 771
42	463026	463446	+	Target "Motif:SIRE3_TC" 504 920
42	463856	464192	+	Target "Motif:SIRE3_TC" 511 837
42	468902	469218	+	Target "Motif:SIRE3_TC" 468 771
42	470473	470904	+	Target "Motif:SIRE3_TC" 257 911
42	471317	471777	+	Target "Motif:SIRE3_TC" 469 836
42	677621	677907	+	Target "Motif:SIRE3_TC" 524 771
42	679161	679587	+	Target "Motif:SIRE3_TC" 504 920

42	680003	680224	+	Target "Motif:SIRE" 95 315
42	680009	680331	+	Target "Motif:SIRE3_TC" 511 774
42	680380	680506	+	Target "Motif:SIRE3_TC" 4269 4386
42	702676	703157	+	Target "Motif:SIRE3_TC" 471 920
42	703994	704469	+	Target "Motif:SIRE3_TC" 394 920
42	705284	705764	+	Target "Motif:SIRE3_TC" 471 920
42	706582	707072	+	Target "Motif:SIRE3_TC" 394 920
42	707887	708354	+	Target "Motif:SIRE3_TC" 471 920
42	709171	709624	+	Target "Motif:SIRE3_TC" 471 920
42	709583	709635	+	Target "Motif:SIRE" 31 83
42	710468	710948	+	Target "Motif:SIRE3_TC" 471 920
42	711771	712249	+	Target "Motif:SIRE3_TC" 471 920
42	713068	713556	+	Target "Motif:SIRE3_TC" 394 920
42	714364	714616	+	Target "Motif:SIRE3_TC" 394 900
42	717725	718002	+	Target "Motif:SIRE3_TC" 667 920
42	718831	719311	+	Target "Motif:SIRE3_TC" 471 920
42	720106	720587	+	Target "Motif:SIRE3_TC" 471 920
42	721384	721863	+	Target "Motif:SIRE3_TC" 471 920
42	722661	723140	+	Target "Motif:SIRE3_TC" 471 920
42	723945	724428	+	Target "Motif:SIRE3_TC" 471 920
42	726393	726871	+	Target "Motif:SIRE3_TC" 471 920
42	727726	727904	+	Target "Motif:SIRE" 95 280
42	729026	729507	+	Target "Motif:SIRE3_TC" 471 920
42	730335	730816	+	Target "Motif:SIRE3_TC" 471 920
42	731638	732128	+	Target "Motif:SIRE3_TC" 394 920
42	732945	733427	+	Target "Motif:SIRE3_TC" 471 920
42	734246	734726	+	Target "Motif:SIRE3_TC" 471 920
42	735551	736040	+	Target "Motif:SIRE3_TC" 394 920
42	736830	737310	+	Target "Motif:SIRE3_TC" 471 920
42	738139	738622	+	Target "Motif:SIRE3_TC" 471 920
42	739494	739643	+	Target "Motif:SIRE" 95 253
42	740639	740926	+	Target "Motif:SIRE3_TC" 394 920
42	741749	742229	+	Target "Motif:SIRE3_TC" 471 920
42	743053	743533	+	Target "Motif:SIRE3_TC" 471 920
42	744358	744840	+	Target "Motif:SIRE3_TC" 471 920
42	745662	746143	+	Target "Motif:SIRE3_TC" 471 920
42	746972	747454	+	Target "Motif:SIRE3_TC" 471 920
42	748282	748769	+	Target "Motif:SIRE3_TC" 394 920
42	749589	750093	+	Target "Motif:SIRE3_TC" 322 920
42	827356	827405	+	Target "Motif:SIRE" 95 141
43	76228	76529	+	Target "Motif:SIRE3_TC" 510 771
43	88538	88809	+	Target "Motif:SIRE3_TC" 518 772
43	89122	89336	+	Target "Motif:SIRE" 95 315
43	89153	89581	+	Target "Motif:SIRE7_TC" 257 696
43	109757	110033	+	Target "Motif:SIRE3_TC" 513 772
43	116215	116627	+	Target "Motif:SIRE3_TC" 511 908

43	123417	123474	-	Target "Motif:SIRE" 91 147
43	241728	241753	-	Target "Motif:SIRE" 284 307
43	241789	241968	-	Target "Motif:SIRE" 96 283
43	245587	245612	-	Target "Motif:SIRE" 284 307
43	245648	245826	-	Target "Motif:SIRE" 96 283
43	249434	249462	-	Target "Motif:SIRE" 285 312
43	249498	249677	-	Target "Motif:SIRE" 96 284
43	253567	253746	-	Target "Motif:SIRE" 95 281
43	257409	257591	-	Target "Motif:SIRE" 95 281
43	261210	261235	-	Target "Motif:SIRE" 284 307
43	261265	261444	-	Target "Motif:SIRE" 96 283
43	265062	265087	-	Target "Motif:SIRE" 284 307
43	265122	265301	-	Target "Motif:SIRE" 96 283
43	269840	269865	-	Target "Motif:SIRE" 284 307
43	269899	270078	-	Target "Motif:SIRE" 96 283
43	273724	273908	-	Target "Motif:SIRE" 95 283
43	277521	277541	-	Target "Motif:SIRE" 284 303
43	277576	277755	-	Target "Motif:SIRE" 96 283
43	281365	281390	-	Target "Motif:SIRE" 284 307
43	281426	281604	-	Target "Motif:SIRE" 96 283
43	285212	285237	-	Target "Motif:SIRE" 284 307
43	285272	285447	-	Target "Motif:SIRE" 96 283
43	290928	291144	-	Target "Motif:SIRE" 95 303
43	294716	294738	-	Target "Motif:SIRE" 282 303
43	294764	294946	-	Target "Motif:SIRE" 96 281
43	298525	298550	-	Target "Motif:SIRE" 284 307
43	298585	298761	-	Target "Motif:SIRE" 96 283
43	302360	302589	-	Target "Motif:SIRE" 95 307
43	306194	306421	-	Target "Motif:SIRE" 95 307
43	310789	310968	-	Target "Motif:SIRE" 95 278
43	314588	314612	-	Target "Motif:SIRE" 284 307
43	314648	314827	-	Target "Motif:SIRE" 96 283
43	318447	318472	-	Target "Motif:SIRE" 284 307
43	318502	318680	-	Target "Motif:SIRE" 96 283
43	322304	322524	-	Target "Motif:SIRE" 95 303
43	326143	326168	-	Target "Motif:SIRE" 284 307
43	326198	326377	-	Target "Motif:SIRE" 96 283
43	329952	330180	-	Target "Motif:SIRE" 95 307
43	333793	333818	-	Target "Motif:SIRE" 284 307
43	333848	334026	-	Target "Motif:SIRE" 96 283
43	337642	337667	-	Target "Motif:SIRE" 284 307
43	337703	337881	-	Target "Motif:SIRE" 96 283
43	341501	341522	-	Target "Motif:SIRE" 284 303
43	341558	341736	-	Target "Motif:SIRE" 96 283
43	345356	345381	-	Target "Motif:SIRE" 284 307
43	345411	345587	-	Target "Motif:SIRE" 96 283

43	349185	349211	-	Target "Motif:SIRE" 282 307
43	349243	349417	-	Target "Motif:SIRE" 96 281
43	353031	353055	-	Target "Motif:SIRE" 284 307
43	353091	353269	-	Target "Motif:SIRE" 96 283
43	356923	357105	-	Target "Motif:SIRE" 95 281
43	360741	360920	-	Target "Motif:SIRE" 95 281
43	365643	365668	-	Target "Motif:SIRE" 284 307
43	365702	365881	-	Target "Motif:SIRE" 96 283
43	369488	369716	-	Target "Motif:SIRE" 95 307
43	373325	373548	-	Target "Motif:SIRE" 95 307
43	377210	377391	-	Target "Motif:SIRE" 95 281
43	379560	379742	-	Target "Motif:SIRE" 95 281
43	385097	385121	-	Target "Motif:SIRE" 284 307
43	385157	385336	-	Target "Motif:SIRE" 96 283
43	388952	388977	-	Target "Motif:SIRE" 284 307
43	389010	389188	-	Target "Motif:SIRE" 96 283
43	392807	392832	-	Target "Motif:SIRE" 284 307
43	392867	393045	-	Target "Motif:SIRE" 96 283
43	396663	396682	-	Target "Motif:SIRE" 285 303
43	396717	396895	-	Target "Motif:SIRE" 96 284
43	400478	400498	-	Target "Motif:SIRE" 284 303
43	400534	400713	-	Target "Motif:SIRE" 96 283
43	404330	404354	-	Target "Motif:SIRE" 284 307
43	404390	404569	-	Target "Motif:SIRE" 96 283
43	408190	408210	-	Target "Motif:SIRE" 284 303
43	408246	408424	-	Target "Motif:SIRE" 96 283
43	412048	412270	-	Target "Motif:SIRE" 95 303
43	415891	415916	-	Target "Motif:SIRE" 284 307
43	415951	416130	-	Target "Motif:SIRE" 96 283
43	419749	419970	-	Target "Motif:SIRE" 95 307
43	423590	423812	-	Target "Motif:SIRE" 95 307
44	10065	10138	-	Target "Motif:SIRE" 33 102
44	19025	19185	-	Target "Motif:SIRE" 119 280
44	47149	47222	-	Target "Motif:SIRE" 33 102
44	51358	51519	-	Target "Motif:SIRE" 119 280
44	89192	89621	-	Target "Motif:SIRE3_TC" 510 920
44	324980	325345	+	Target "Motif:SIRE6_TC" 534 900
44	325595	326180	-	Target "Motif:SIRE3_TC" 347 920
44	326026	326528	+	Target "Motif:SIRE6_TC" 399 900
44	326778	327360	-	Target "Motif:SIRE3_TC" 347 920
44	327206	327708	+	Target "Motif:SIRE6_TC" 399 900
44	327958	328544	-	Target "Motif:SIRE3_TC" 347 920
44	328363	328892	+	Target "Motif:SIRE6_TC" 371 900
44	329141	329725	-	Target "Motif:SIRE3_TC" 347 920
44	329571	330073	+	Target "Motif:SIRE6_TC" 399 900
44	330321	330905	-	Target "Motif:SIRE3_TC" 347 920

44	330751	331253	+	Target "Motif:SIRE6_TC" 399 900
44	331502	332086	-	Target "Motif:SIRE3_TC" 347 920
44	331905	332434	+	Target "Motif:SIRE6_TC" 371 900
44	332684	333268	-	Target "Motif:SIRE3_TC" 347 920
44	333114	333616	+	Target "Motif:SIRE6_TC" 399 900
44	333865	334446	-	Target "Motif:SIRE3_TC" 347 920
44	334293	334793	+	Target "Motif:SIRE6_TC" 400 900
44	334293	334793	+	Target "Motif:SIRE6_TC" 400 900
44	335041	335624	-	Target "Motif:SIRE3_TC" 347 920
44	335470	335971	+	Target "Motif:SIRE6_TC" 399 900
44	335470	335971	+	Target "Motif:SIRE6_TC" 399 900
44	336218	336798	-	Target "Motif:SIRE3_TC" 347 920
44	336645	337144	+	Target "Motif:SIRE6_TC" 400 900
44	336645	337144	+	Target "Motif:SIRE6_TC" 400 900
44	336645	337144	+	Target "Motif:SIRE6_TC" 400 900
44	337389	337969	-	Target "Motif:SIRE3_TC" 347 920
44	337788	338313	+	Target "Motif:SIRE6_TC" 371 900
44	338556	339135	-	Target "Motif:SIRE3_TC" 347 920
44	338984	339478	+	Target "Motif:SIRE6_TC" 400 900
44	339725	340302	-	Target "Motif:SIRE3_TC" 347 920
44	340151	340650	+	Target "Motif:SIRE6_TC" 400 900
44	340900	341486	-	Target "Motif:SIRE3_TC" 347 920
44	341305	341833	+	Target "Motif:SIRE6_TC" 371 900
44	341305	341833	+	Target "Motif:SIRE6_TC" 371 900
44	342082	342665	-	Target "Motif:SIRE3_TC" 347 920
44	342485	343010	+	Target "Motif:SIRE6_TC" 371 900
44	342485	343010	+	Target "Motif:SIRE6_TC" 371 900
44	342485	343010	+	Target "Motif:SIRE6_TC" 371 900
44	343259	343844	-	Target "Motif:SIRE3_TC" 347 920
44	343691	344190	+	Target "Motif:SIRE6_TC" 400 900
44	343691	344190	+	Target "Motif:SIRE6_TC" 400 900
44	343691	344190	+	Target "Motif:SIRE6_TC" 400 900
44	344438	345020	-	Target "Motif:SIRE3_TC" 347 920
44	344867	345368	+	Target "Motif:SIRE6_TC" 400 900
44	345617	346202	-	Target "Motif:SIRE3_TC" 347 920
44	346049	346535	+	Target "Motif:SIRE6_TC" 400 900
44	346782	347367	-	Target "Motif:SIRE3_TC" 347 920
44	347186	347713	+	Target "Motif:SIRE6_TC" 371 900
44	347186	347713	+	Target "Motif:SIRE6_TC" 371 900
44	347186	347713	+	Target "Motif:SIRE6_TC" 371 900
44	347962	348546	-	Target "Motif:SIRE3_TC" 347 920
44	348365	348893	+	Target "Motif:SIRE6_TC" 371 900
44	348365	348893	+	Target "Motif:SIRE6_TC" 371 900
44	349142	349728	-	Target "Motif:SIRE3_TC" 347 920
44	349547	350075	+	Target "Motif:SIRE6_TC" 371 900
44	349547	350075	+	Target "Motif:SIRE6_TC" 371 900

44	350321	350902	-	Target "Motif:SIRE3_TC" 347 920
44	350750	351248	+	Target "Motif:SIRE6_TC" 400 900
44	350750	351248	+	Target "Motif:SIRE6_TC" 400 900
44	351496	352076	-	Target "Motif:SIRE3_TC" 347 920
44	351922	352422	+	Target "Motif:SIRE6_TC" 399 900
44	351922	352422	+	Target "Motif:SIRE6_TC" 399 900
44	351922	352422	+	Target "Motif:SIRE6_TC" 399 900
44	352669	353249	-	Target "Motif:SIRE3_TC" 347 920
44	353097	353594	+	Target "Motif:SIRE6_TC" 400 900
44	353097	353594	+	Target "Motif:SIRE6_TC" 400 900
44	353097	353594	+	Target "Motif:SIRE6_TC" 400 900
44	353097	353594	+	Target "Motif:SIRE6_TC" 400 900
44	353838	354414	-	Target "Motif:SIRE3_TC" 347 920
44	354261	354747	+	Target "Motif:SIRE6_TC" 400 891
44	355000	355571	-	Target "Motif:SIRE3_TC" 347 920
44	355424	355913	+	Target "Motif:SIRE6_TC" 399 900
44	355424	355913	+	Target "Motif:SIRE6_TC" 399 900
44	355424	355913	+	Target "Motif:SIRE6_TC" 399 900
44	356158	356731	-	Target "Motif:SIRE3_TC" 347 920
44	356584	357074	+	Target "Motif:SIRE6_TC" 400 900
44	356584	357074	+	Target "Motif:SIRE6_TC" 400 900
44	356584	357074	+	Target "Motif:SIRE6_TC" 400 900
44	356584	357074	+	Target "Motif:SIRE6_TC" 400 900
44	356584	357074	+	Target "Motif:SIRE6_TC" 400 900
44	357316	357881	-	Target "Motif:SIRE3_TC" 347 920
44	357732	358209	+	Target "Motif:SIRE6_TC" 400 900
44	357732	358209	+	Target "Motif:SIRE6_TC" 400 900
44	357732	358209	+	Target "Motif:SIRE6_TC" 400 900
44	357732	358209	+	Target "Motif:SIRE6_TC" 400 900
44	357732	358209	+	Target "Motif:SIRE6_TC" 400 900
44	357732	358209	+	Target "Motif:SIRE6_TC" 400 900
44	357732	358209	+	Target "Motif:SIRE6_TC" 400 900
44	358450	359031	-	Target "Motif:SIRE3_TC" 347 920
44	358850	359379	+	Target "Motif:SIRE6_TC" 371 900
44	359629	360215	-	Target "Motif:SIRE3_TC" 347 920
44	360034	360557	+	Target "Motif:SIRE6_TC" 371 900
44	360807	361392	-	Target "Motif:SIRE3_TC" 347 920
44	361238	361740	+	Target "Motif:SIRE6_TC" 399 900
44	361989	362574	-	Target "Motif:SIRE3_TC" 347 920
44	362420	362922	+	Target "Motif:SIRE6_TC" 399 900
44	363172	363756	-	Target "Motif:SIRE3_TC" 347 920
44	363602	364104	+	Target "Motif:SIRE6_TC" 399 900
44	364353	364938	-	Target "Motif:SIRE3_TC" 347 920
44	364784	365286	+	Target "Motif:SIRE6_TC" 399 900
44	365535	366119	-	Target "Motif:SIRE3_TC" 347 920
44	365965	366467	+	Target "Motif:SIRE6_TC" 399 900

44	366716	367300	-	Target "Motif:SIRE3_TC" 347 920
44	367147	367648	+	Target "Motif:SIRE6_TC" 400 900
44	367897	368482	-	Target "Motif:SIRE3_TC" 347 920
44	368328	368830	+	Target "Motif:SIRE6_TC" 399 900
44	369079	369664	-	Target "Motif:SIRE3_TC" 347 920
44	369510	370011	+	Target "Motif:SIRE6_TC" 399 900
44	369510	370011	+	Target "Motif:SIRE6_TC" 399 900
44	370261	370845	-	Target "Motif:SIRE3_TC" 347 920
44	370691	371193	+	Target "Motif:SIRE6_TC" 399 900
44	371442	372026	-	Target "Motif:SIRE3_TC" 347 920
44	371872	372373	+	Target "Motif:SIRE6_TC" 399 900
44	371872	372373	+	Target "Motif:SIRE6_TC" 399 900
44	372622	373207	-	Target "Motif:SIRE3_TC" 347 920
44	373053	373555	+	Target "Motif:SIRE6_TC" 399 900
44	373804	374389	-	Target "Motif:SIRE3_TC" 347 920
44	374235	374735	+	Target "Motif:SIRE6_TC" 399 900
44	374984	375567	-	Target "Motif:SIRE3_TC" 347 920
44	375413	375913	+	Target "Motif:SIRE6_TC" 399 900
44	375413	375913	+	Target "Motif:SIRE6_TC" 399 900
44	375413	375913	+	Target "Motif:SIRE6_TC" 399 900
44	376161	376740	-	Target "Motif:SIRE3_TC" 347 920
44	376589	377088	+	Target "Motif:SIRE6_TC" 401 900
44	377338	377924	-	Target "Motif:SIRE3_TC" 347 920
44	377743	378272	+	Target "Motif:SIRE6_TC" 371 900
44	378522	379099	-	Target "Motif:SIRE3_TC" 347 920
44	378949	379445	+	Target "Motif:SIRE6_TC" 400 900
44	379694	380273	-	Target "Motif:SIRE3_TC" 347 920
44	380119	380620	+	Target "Motif:SIRE6_TC" 399 900
44	380869	381449	-	Target "Motif:SIRE3_TC" 347 920
44	381295	381795	+	Target "Motif:SIRE6_TC" 399 900
44	382045	382624	-	Target "Motif:SIRE3_TC" 347 920
44	382470	382972	+	Target "Motif:SIRE6_TC" 399 900
44	383221	383801	-	Target "Motif:SIRE3_TC" 347 920
44	383647	384147	+	Target "Motif:SIRE6_TC" 399 900
44	384395	384973	-	Target "Motif:SIRE3_TC" 347 920
44	384783	385307	+	Target "Motif:SIRE6_TC" 358 900
44	384783	385307	+	Target "Motif:SIRE6_TC" 358 900
44	384783	385307	+	Target "Motif:SIRE6_TC" 358 900
44	384783	385307	+	Target "Motif:SIRE6_TC" 358 900
44	385544	386112	-	Target "Motif:SIRE3_TC" 347 920
44	385934	386457	+	Target "Motif:SIRE6_TC" 371 900
44	385934	386457	+	Target "Motif:SIRE6_TC" 371 900
44	385934	386457	+	Target "Motif:SIRE6_TC" 371 900
44	385934	386457	+	Target "Motif:SIRE6_TC" 371 900
44	386707	387287	-	Target "Motif:SIRE3_TC" 347 920
44	387133	387629	+	Target "Motif:SIRE6_TC" 399 900

44	387879	388460	-	Target "Motif:SIRE3_TC" 347 920
44	388307	388807	+	Target "Motif:SIRE6_TC" 399 900
44	389055	389640	-	Target "Motif:SIRE3_TC" 347 920
44	389486	389987	+	Target "Motif:SIRE6_TC" 399 900
44	390237	390820	-	Target "Motif:SIRE3_TC" 347 920
44	390666	391168	+	Target "Motif:SIRE6_TC" 399 900
44	391418	392002	-	Target "Motif:SIRE3_TC" 347 920
44	391848	392348	+	Target "Motif:SIRE6_TC" 399 900
44	392598	393180	-	Target "Motif:SIRE3_TC" 347 920
44	393027	393528	+	Target "Motif:SIRE6_TC" 400 900
44	393776	394361	-	Target "Motif:SIRE3_TC" 347 920
44	394207	394708	+	Target "Motif:SIRE6_TC" 399 900
44	394207	394708	+	Target "Motif:SIRE6_TC" 399 900
44	394956	395535	-	Target "Motif:SIRE3_TC" 347 920
44	395385	395866	+	Target "Motif:SIRE6_TC" 399 900
44	396110	396258	-	Target "Motif:SIRE3_TC" 779 916
44	403242	403743	-	Target "Motif:SIRE3_TC" 347 851
44	403589	404091	+	Target "Motif:SIRE6_TC" 399 900
44	404351	404850	+	Target "Motif:SIRE6_TC" 400 900
44	405113	405614	+	Target "Motif:SIRE6_TC" 400 900
44	405876	406377	+	Target "Motif:SIRE6_TC" 400 900
44	406640	407141	+	Target "Motif:SIRE6_TC" 400 900
44	407403	407905	+	Target "Motif:SIRE6_TC" 400 900
44	408167	408668	+	Target "Motif:SIRE6_TC" 400 900
44	408930	409430	+	Target "Motif:SIRE6_TC" 400 900
45	9608	9740	-	Target "Motif:SIRE3_TC" 834 920
45	9775	10088	-	Target "Motif:SIRE3_TC" 506 833
45	57578	58011	-	Target "Motif:SIRE3_TC" 502 920
45	276166	276270	-	Target "Motif:SIRE" 98 202
45	285642	285833	+	Target "Motif:SIRE" 119 313
45	343392	343641	+	Target "Motif:SIRE3_TC" 529 771
46	186012	186061	-	Target "Motif:SIRE" 95 145
46	224630	224680	-	Target "Motif:SIRE" 94 142
46	351757	351987	+	Target "Motif:SIRE" 94 313
46	357879	358089	+	Target "Motif:SIRE" 95 300
46	366238	366457	+	Target "Motif:SIRE" 95 313
46	366457	366672	+	Target "Motif:SIRE3_TC" 359 901
47	194136	194180	-	Target "Motif:SIRE" 101 145
47	204808	204851	-	Target "Motif:SIRE" 101 145
47	205073	205286	-	Target "Motif:SIRE" 97 315
47	216541	216584	-	Target "Motif:SIRE" 100 145
47	245577	245620	-	Target "Motif:SIRE" 99 147
47	315503	315831	-	Target "Motif:SIRE3_TC" 504 774
47	319156	319482	-	Target "Motif:SIRE3_TC" 504 774
47	344953	345395	-	Target "Motif:SIRE3_TC" 504 774
47	347069	347412	-	Target "Motif:SIRE3_TC" 504 774

47	348967	349402	-	Target "Motif:SIRE3_TC" 504 774
47	372535	373002	-	Target "Motif:SIRE3_TC" 483 920
5	862	1088	-	Target "Motif:SIRE" 95 307
5	4697	4926	-	Target "Motif:SIRE" 95 312
5	8536	8771	-	Target "Motif:SIRE" 95 312
5	12432	12615	-	Target "Motif:SIRE" 95 283
5	18300	18525	-	Target "Motif:SIRE" 95 312
5	22130	22357	-	Target "Motif:SIRE" 95 307
5	25977	26002	-	Target "Motif:SIRE" 284 307
5	26037	26216	-	Target "Motif:SIRE" 96 283
5	29836	29859	-	Target "Motif:SIRE" 284 307
5	29894	30073	-	Target "Motif:SIRE" 96 283
5	108120	108337	-	Target "Motif:SIRE" 98 293
5	246385	246830	-	Target "Motif:SIRE3_TC" 504 920
5	431500	431879	+	Target "Motif:SIRE3_TC" 395 821
5	520148	520364	+	Target "Motif:SIRE" 95 315
5	520154	520452	+	Target "Motif:SIRE3_TC" 511 771
5	527339	527392	-	Target "Motif:SIRE" 95 147
5	765322	765544	+	Target "Motif:SIRE" 102 281
5	765547	765729	+	Target "Motif:SIRE" 95 281
5	779362	779846	+	Target "Motif:SIRE3_TC" 390 832
5	779751	780044	+	Target "Motif:SIRE" 31 281
5	780047	780230	+	Target "Motif:SIRE" 95 281
5	786990	787468	+	Target "Motif:SIRE3_TC" 390 832
5	787377	787669	+	Target "Motif:SIRE" 31 281
5	787671	787854	+	Target "Motif:SIRE" 95 281
5	825431	825625	+	Target "Motif:SIRE" 97 281
5	825628	825810	+	Target "Motif:SIRE" 95 281
5	838552	839032	+	Target "Motif:SIRE3_TC" 390 911
5	838939	839226	+	Target "Motif:SIRE" 31 281
5	839229	839412	+	Target "Motif:SIRE" 95 281
5	852221	852702	+	Target "Motif:SIRE3_TC" 390 911
5	852706	852900	+	Target "Motif:SIRE" 97 281
5	852903	853086	+	Target "Motif:SIRE" 95 281
5	1188796	1188982	-	Target "Motif:SIRE" 95 281
6	2807	3024	-	Target "Motif:SIRE" 101 313
6	120341	120661	+	Target "Motif:SIRE3_TC" 494 771
6	268741	268787	-	Target "Motif:SIRE" 98 145
6	297764	297810	+	Target "Motif:SIRE" 98 146
6	463411	463724	-	Target "Motif:SIRE3_TC" 670 920
6	491628	492144	-	Target "Motif:SIRE3_TC" 480 836
6	496784	496828	-	Target "Motif:SIRE" 100 145
6	506801	506843	-	Target "Motif:SIRE" 100 146
6	507064	507276	-	Target "Motif:SIRE" 97 315
6	590637	590964	-	Target "Motif:SIRE3_TC" 504 774
6	592671	592999	-	Target "Motif:SIRE3_TC" 504 774

6	594699	595027	-	Target "Motif:SIRE3_TC" 504 774
6	596734	597062	-	Target "Motif:SIRE3_TC" 504 774
6	605963	606411	-	Target "Motif:SIRE3_TC" 504 774
6	608121	608448	-	Target "Motif:SIRE3_TC" 504 774
6	610033	610482	-	Target "Motif:SIRE3_TC" 504 774
6	612197	612523	-	Target "Motif:SIRE3_TC" 504 774
6	614271	614554	-	Target "Motif:SIRE3_TC" 504 774
6	665690	666018	-	Target "Motif:SIRE3_TC" 504 774
6	667733	668059	-	Target "Motif:SIRE3_TC" 504 774
6	669649	670096	-	Target "Motif:SIRE3_TC" 504 774
6	671851	672136	-	Target "Motif:SIRE3_TC" 504 774
6	686822	687150	-	Target "Motif:SIRE3_TC" 504 774
6	750735	751013	-	Target "Motif:SIRE3_TC" 667 920
6	768858	769249	-	Target "Motif:SIRE3_TC" 513 870
6	783002	783458	-	Target "Motif:SIRE3_TC" 513 920
6	819020	819493	-	Target "Motif:SIRE3_TC" 492 920
6	846551	846597	-	Target "Motif:SIRE" 98 146
6	874090	874584	+	Target "Motif:SIRE3_TC" 480 782
6	881351	881860	+	Target "Motif:SIRE3_TC" 480 780
6	888853	889361	+	Target "Motif:SIRE3_TC" 480 839
6	912902	913406	-	Target "Motif:SIRE3_TC" 480 836
6	950518	951021	+	Target "Motif:SIRE3_TC" 480 836
6	958032	958518	+	Target "Motif:SIRE3_TC" 480 836
6	1283552	1283757	+	Target "Motif:SIRE" 95 297
7	113663	113929	-	Target "Motif:SIRE3_TC" 485 836
7	113929	114146	-	Target "Motif:SIRE" 95 315
7	119292	119506	-	Target "Motif:SIRE" 94 297
7	160549	160966	+	Target "Motif:SIRE3_TC" 509 919
7	175255	175725	+	Target "Motif:SIRE3_TC" 492 920
7	607672	608149	-	Target "Motif:SIRE3_TC" 474 920
7	1114741	1115046	+	Target "Motif:SIRE3_TC" 519 771
7	1174198	1174504	+	Target "Motif:SIRE3_TC" 512 771
7	1179470	1179518	-	Target "Motif:SIRE" 95 150
7	1308811	1308860	-	Target "Motif:SIRE" 95 151
7	1523618	1523969	-	Target "Motif:SIRE3_TC" 510 782
7	1597767	1597845	-	Target "Motif:SIRE" 195 275
7	1597846	1597884	-	Target "Motif:SIRE" 95 132
7	1658765	1658941	+	Target "Motif:SIRE" 100 281
7	1908610	1908796	-	Target "Motif:SIRE" 98 282
7	2093177	2093680	-	Target "Motif:SIRE3_TC" 480 779
7	2125369	2125709	-	Target "Motif:SIRE3_TC" 510 779
8	72459	72794	-	Target "Motif:SIRE3_TC" 510 771
8	103282	103323	+	Target "Motif:SIRE" 103 140
8	743583	743625	+	Target "Motif:SIRE" 102 146
8	846719	846891	+	Target "Motif:SIRE3_TC" 680 841
8	896195	896336	+	Target "Motif:SIRE3_TC" 709 841

8	910243	910694	+	Target "Motif:SIRE3_TC" 494 920
8	919549	919774	+	Target "Motif:SIRE" 94 313
8	919774	919991	+	Target "Motif:SIRE3_TC" 506 899
8	929180	929647	+	Target "Motif:SIRE3_TC" 394 894
8	932865	933324	+	Target "Motif:SIRE3_TC" 394 894
8	936574	937035	+	Target "Motif:SIRE3_TC" 394 894
8	940295	940754	+	Target "Motif:SIRE3_TC" 260 894
8	944017	944483	+	Target "Motif:SIRE3_TC" 394 894
8	947741	948207	+	Target "Motif:SIRE3_TC" 394 894
8	951450	951916	+	Target "Motif:SIRE3_TC" 394 894
8	955628	955813	+	Target "Motif:SIRE" 94 279
8	967440	967886	+	Target "Motif:SIRE3_TC" 501 920
8	1093687	1093997	-	Target "Motif:SIRE3_TC" 510 771
8	1111458	1111914	-	Target "Motif:SIRE3_TC" 257 855
9	7273	7431	+	Target "Motif:SIRE" 105 275
9	88993	89151	-	Target "Motif:SIRE" 105 275
9	103749	103878	-	Target "Motif:SIRE3_TC" 508 776
9	103878	104101	-	Target "Motif:SIRE" 98 316
9	122968	123398	-	Target "Motif:SIRE3_TC" 509 920
9	313633	314113	-	Target "Motif:SIRE3_TC" 491 920
9	338428	338911	-	Target "Motif:SIRE3_TC" 491 920
9	370451	370647	-	Target "Motif:SIRE3_TC" 725 898
9	388808	388853	-	Target "Motif:SIRE3_TC" 503 547
9	500630	501051	-	Target "Motif:SIRE3_TC" 504 919
9	502976	503397	-	Target "Motif:SIRE3_TC" 504 919
9	505322	505742	-	Target "Motif:SIRE3_TC" 504 919
9	507667	508090	-	Target "Motif:SIRE3_TC" 504 919
9	510008	510429	-	Target "Motif:SIRE3_TC" 504 919
9	512351	512771	-	Target "Motif:SIRE3_TC" 504 919
9	514696	515116	-	Target "Motif:SIRE3_TC" 504 919
9	517006	517427	-	Target "Motif:SIRE3_TC" 504 919
9	519341	519762	-	Target "Motif:SIRE3_TC" 504 919
9	521685	522105	-	Target "Motif:SIRE3_TC" 504 919
9	524024	524444	-	Target "Motif:SIRE3_TC" 504 919
9	526367	526788	-	Target "Motif:SIRE3_TC" 504 919
9	539607	540028	-	Target "Motif:SIRE3_TC" 504 919
9	541955	542374	-	Target "Motif:SIRE3_TC" 504 919
9	544296	544720	-	Target "Motif:SIRE3_TC" 504 919
9	546643	547062	-	Target "Motif:SIRE3_TC" 504 919
9	548986	549404	-	Target "Motif:SIRE3_TC" 504 919
9	551324	551744	-	Target "Motif:SIRE3_TC" 504 919
9	553660	554078	-	Target "Motif:SIRE3_TC" 504 919
9	555995	556413	-	Target "Motif:SIRE3_TC" 504 919
9	558416	558788	-	Target "Motif:SIRE3_TC" 474 830
9	560652	561074	-	Target "Motif:SIRE3_TC" 504 919
9	564672	565122	-	Target "Motif:SIRE3_TC" 504 919

9	567036	567456	-	Target "Motif:SIRE3_TC" 504 919
9	569377	569794	-	Target "Motif:SIRE3_TC" 504 913
9	571710	572126	-	Target "Motif:SIRE3_TC" 504 913
9	574048	574462	-	Target "Motif:SIRE3_TC" 504 913
9	578694	579108	-	Target "Motif:SIRE3_TC" 504 913
9	581113	581444	-	Target "Motif:SIRE3_TC" 504 828
9	583377	583793	-	Target "Motif:SIRE3_TC" 504 913
9	585806	586137	-	Target "Motif:SIRE3_TC" 504 830
9	588066	588482	-	Target "Motif:SIRE3_TC" 504 913
9	590405	590820	-	Target "Motif:SIRE3_TC" 504 913
9	592749	593164	-	Target "Motif:SIRE3_TC" 504 913
9	595094	595509	-	Target "Motif:SIRE3_TC" 504 913
9	597433	597872	-	Target "Motif:SIRE3_TC" 494 913
9	700752	701172	+	Target "Motif:SIRE3_TC" 504 919
9	703095	703515	+	Target "Motif:SIRE3_TC" 504 919
9	705439	705859	+	Target "Motif:SIRE3_TC" 504 919
9	707786	708097	+	Target "Motif:SIRE3_TC" 504 826
9	710064	710482	+	Target "Motif:SIRE3_TC" 504 919
9	712391	712814	+	Target "Motif:SIRE3_TC" 504 919
9	714729	715151	+	Target "Motif:SIRE3_TC" 504 919
9	717062	717484	+	Target "Motif:SIRE3_TC" 504 919
9	719401	719821	+	Target "Motif:SIRE3_TC" 504 919
9	721730	722150	+	Target "Motif:SIRE3_TC" 504 919
9	724066	724488	+	Target "Motif:SIRE3_TC" 504 919
9	726410	726850	+	Target "Motif:SIRE3_TC" 504 830
9	730084	730505	+	Target "Motif:SIRE3_TC" 504 919
9	732422	732843	+	Target "Motif:SIRE3_TC" 504 919
9	734767	735188	+	Target "Motif:SIRE3_TC" 504 919
9	737112	737533	+	Target "Motif:SIRE3_TC" 504 919
9	739457	739877	+	Target "Motif:SIRE3_TC" 504 919
9	741799	742213	+	Target "Motif:SIRE3_TC" 504 913
9	744142	744556	+	Target "Motif:SIRE3_TC" 504 913
9	746476	746892	+	Target "Motif:SIRE3_TC" 504 913
9	748784	749200	+	Target "Motif:SIRE3_TC" 504 913
9	751124	751542	+	Target "Motif:SIRE3_TC" 504 913
9	753470	753885	+	Target "Motif:SIRE3_TC" 504 913
9	755810	756142	+	Target "Motif:SIRE3_TC" 504 830
9	758151	758566	+	Target "Motif:SIRE3_TC" 504 913
9	760488	760905	+	Target "Motif:SIRE3_TC" 504 913
9	764678	765165	+	Target "Motif:SIRE3_TC" 503 920
9	767158	767610	+	Target "Motif:SIRE3_TC" 468 913
9	769539	769953	+	Target "Motif:SIRE3_TC" 504 913
9	773730	774210	+	Target "Motif:SIRE3_TC" 503 920
9	889106	889429	+	Target "Motif:SIRE3_TC" 480 771
9	1058921	1059129	+	Target "Motif:SIRE" 94 297
9	1110341	1110548	+	Target "Motif:SIRE" 94 297

9	1118937	1119142	+	Target "Motif:SIRE" 94 297
