# Supplementary material for: Repeat-Driven Generation of Antigenic Diversity in a Major Human Pathogen, Trypanosoma cruzi
Source: Front Cell Infect Microbiol. 2021 Mar 3;11:614665. doi: 10.3389/fcimb.2021.614665 (PMC7966520; doi:10.3389/fcimb.2021.614665)
Supplement: Supplementary file 6 [file DataSheet_6.pdf]

Supplementary table 5.

Chromosome	startR	stopR	strand	repeat
1	430608	431780	+	Target "Motif:L1_TC" 3641 4823
1	1001422	1003258	+	Target "Motif:L1_TC" 2382 4225
1	1001422	1003258	+	Target "Motif:L1_TC" 2382 4225
1	1003249	1003440	+	Target "Motif:L1_TC" 4624 4814
1	1008658	1008733	+	Target "Motif:L1_TC" 137 212
1	1031930	1035365	+	Target "Motif:L1_TC" 694 4225
1	1031930	1035365	+	Target "Motif:L1_TC" 694 4225
1	1035356	1035551	+	Target "Motif:L1_TC" 4624 4819
1	1058840	1059670	+	Target "Motif:L1_TC" 3395 4225
1	1058840	1059670	+	Target "Motif:L1_TC" 3395 4225
1	1059661	1059857	+	Target "Motif:L1_TC" 4624 4819
1	1679513	1679729	-	Target "Motif:L1_TC" 1 213
1	1916646	1916877	+	Target "Motif:L1_TC" 1 248
1	2499942	2500018	-	Target "Motif:L1_TC" 1 55
1	2502956	2503186	-	Target "Motif:L1_TC" 1 248
1	2675347	2680117	+	Target "Motif:L1_TC" 4 4815
1	2675347	2680117	+	Target "Motif:L1_TC" 4 4815
10	381843	383172	-	Target "Motif:L1_TC" 3483 4826
10	381843	383172	-	Target "Motif:L1_TC" 3483 4826
11	109985	114884	-	Target "Motif:L1_TC" 1 4831
11	109985	114884	-	Target "Motif:L1_TC" 1 4831
11	109985	114884	-	Target "Motif:L1_TC" 1 4831
11	197619	202508	-	Target "Motif:L1_TC" 1 4831
11	197619	202508	-	Target "Motif:L1_TC" 1 4831
11	197619	202508	-	Target "Motif:L1_TC" 1 4831
11	197619	202508	-	Target "Motif:L1_TC" 1 4831
11	259362	264081	+	Target "Motif:L1_TC" 2 4821
11	259362	264081	+	Target "Motif:L1_TC" 2 4821
11	268396	273114	-	Target "Motif:L1_TC" 2 4818
11	268396	273114	-	Target "Motif:L1_TC" 2 4818
11	268396	273114	-	Target "Motif:L1_TC" 2 4818
11	1009721	1014428	+	Target "Motif:L1_TC" 1 4817
11	1009721	1014428	+	Target "Motif:L1_TC" 1 4817
11	1009721	1014428	+	Target "Motif:L1_TC" 1 4817
11	1009721	1014428	+	Target "Motif:L1_TC" 1 4817
11	1022495	1027206	+	Target "Motif:L1_TC" 1 4817
12	11016	11233	+	Target "Motif:L1_TC" 1 219
12	18312	18531	+	Target "Motif:L1_TC" 1 219
12	942743	947477	-	Target "Motif:L1_TC" 139 4821
12	942743	947477	-	Target "Motif:L1_TC" 139 4821
12	942743	947477	-	Target "Motif:L1_TC" 139 4821
13	9258	9477	+	Target "Motif:L1_TC" 1 219

13	141400	146029	-	Target "Motif:L1_TC" 1 4820
13	141400	146029	-	Target "Motif:L1_TC" 1 4820
13	141400	146029	-	Target "Motif:L1_TC" 1 4820
13	181360	185978	-	Target "Motif:L1_TC" 1 4820
13	210763	215392	+	Target "Motif:L1_TC" 1 4820
13	572525	577231	+	Target "Motif:L1_TC" 1 4819
13	572525	577231	+	Target "Motif:L1_TC" 1 4819
13	591542	596233	+	Target "Motif:L1_TC" 1 4816
13	935561	940228	+	Target "Motif:L1_TC" 1 4818
13	951407	956105	+	Target "Motif:L1_TC" 1 4821
13	951407	956105	+	Target "Motif:L1_TC" 1 4821
13	951407	956105	+	Target "Motif:L1_TC" 1 4821
13	951407	956105	+	Target "Motif:L1_TC" 1 4821
14	276423	277747	-	Target "Motif:L1_TC" 3481 4815
14	419944	421253	+	Target "Motif:L1_TC" 3496 4824
14	451996	456860	-	Target "Motif:L1_TC" 1 4825
14	508542	509847	-	Target "Motif:L1_TC" 3496 4821
14	548430	549743	+	Target "Motif:L1_TC" 3496 4823
14	567042	568356	+	Target "Motif:L1_TC" 3496 4816
15	114454	114684	+	Target "Motif:L1_TC" 1 255
15	268014	268351	-	Target "Motif:L1_TC" 4477 4815
15	279491	279810	-	Target "Motif:L1_TC" 165 488
15	290295	290565	-	Target "Motif:L1_TC" 4546 4820
15	290295	290565	-	Target "Motif:L1_TC" 4546 4820
15	290295	290565	-	Target "Motif:L1_TC" 4546 4820
15	290295	290565	-	Target "Motif:L1_TC" 4546 4820
15	290557	290915	-	Target "Motif:L1_TC" 3655 4049
15	297023	297242	-	Target "Motif:L1_TC" 1 219
15	307262	312058	-	Target "Motif:L1_TC" 1 4819
15	307262	312058	-	Target "Motif:L1_TC" 1 4819
15	307262	312058	-	Target "Motif:L1_TC" 1 4819
15	436115	440815	-	Target "Motif:L1_TC" 4 4824
15	447695	448874	-	Target "Motif:L1_TC" 3641 4821
15	781761	781976	+	Target "Motif:L1_TC" 1 213
16	168555	168783	+	Target "Motif:L1_TC" 1 248
16	358227	358512	-	Target "Motif:L1_TC" 4546 4830
16	358505	358870	-	Target "Motif:L1_TC" 3655 4048
16	363652	363929	-	Target "Motif:L1_TC" 4546 4822
16	363922	364285	-	Target "Motif:L1_TC" 3655 4048
16	368651	368923	-	Target "Motif:L1_TC" 4546 4821
16	368915	372585	-	Target "Motif:L1_TC" 180 4049
16	368915	372585	-	Target "Motif:L1_TC" 180 4049
16	387900	388176	-	Target "Motif:L1_TC" 4546 4822

16	388168	391842	-	Target "Motif:L1_TC" 180 4049
16	405912	406186	-	Target "Motif:L1_TC" 4546 4821
16	406178	409851	-	Target "Motif:L1_TC" 180 4049
16	414878	415153	-	Target "Motif:L1_TC" 4546 4821
16	415145	415511	-	Target "Motif:L1_TC" 3655 4049
16	420825	421100	-	Target "Motif:L1_TC" 4546 4821
16	421092	424768	-	Target "Motif:L1_TC" 180 4049
16	443712	443927	+	Target "Motif:L1_TC" 1 213
16	450012	450369	+	Target "Motif:L1_TC" 3655 4049
16	450361	450634	+	Target "Motif:L1_TC" 4546 4821
16	451879	455539	+	Target "Motif:L1_TC" 180 4049
16	455531	455806	+	Target "Motif:L1_TC" 4546 4822
16	476035	479707	+	Target "Motif:L1_TC" 180 4049
16	479699	479984	+	Target "Motif:L1_TC" 4546 4830
16	481295	481511	-	Target "Motif:L1_TC" 1 213
16	481522	485970	-	Target "Motif:L1_TC" 180 4822
16	481522	485970	-	Target "Motif:L1_TC" 180 4822
16	488310	488621	+	Target "Motif:L1_TC" 180 491
16	499728	500053	+	Target "Motif:L1_TC" 4481 4815
16	503986	506375	+	Target "Motif:L1_TC" 2388 4826
16	508899	510037	+	Target "Motif:L1_TC" 3681 4823
16	510048	514788	+	Target "Motif:L1_TC" 1 4823
16	510048	514788	+	Target "Motif:L1_TC" 1 4823
16	514799	519539	+	Target "Motif:L1_TC" 1 4823
16	514799	519539	+	Target "Motif:L1_TC" 1 4823
16	514799	519539	+	Target "Motif:L1_TC" 1 4823
16	519550	524290	+	Target "Motif:L1_TC" 1 4823
16	519550	524290	+	Target "Motif:L1_TC" 1 4823
16	524301	528596	+	Target "Motif:L1_TC" 1 4382
16	533518	538319	+	Target "Motif:L1_TC" 1 4826
16	533518	538319	+	Target "Motif:L1_TC" 1 4826
16	533518	538319	+	Target "Motif:L1_TC" 1 4826
16	543615	547887	-	Target "Motif:L1_TC" 1 4384
16	548042	552720	+	Target "Motif:L1_TC" 1 4819
16	559119	562822	+	Target "Motif:L1_TC" 1016 4824
16	583595	588331	-	Target "Motif:L1_TC" 1 4824
16	583595	588331	-	Target "Motif:L1_TC" 1 4824
16	588342	593082	-	Target "Motif:L1_TC" 1 4823
16	588342	593082	-	Target "Motif:L1_TC" 1 4823
16	593093	597834	-	Target "Motif:L1_TC" 1 4823
16	593093	597834	-	Target "Motif:L1_TC" 1 4823
16	593093	597834	-	Target "Motif:L1_TC" 1 4823
16	597845	602588	-	Target "Motif:L1_TC" 1 4823

16	597845	602588	-	Target "Motif:L1_TC" 1 4823
16	602599	607332	-	Target "Motif:L1_TC" 1 4823
16	602599	607332	-	Target "Motif:L1_TC" 1 4823
16	602599	607332	-	Target "Motif:L1_TC" 1 4823
16	607343	608492	-	Target "Motif:L1_TC" 3681 4822
16	608869	611191	-	Target "Motif:L1_TC" 1 2256
16	611200	616076	-	Target "Motif:L1_TC" 1 4826
16	616072	619805	-	Target "Motif:L1_TC" 990 4827
16	620951	624651	-	Target "Motif:L1_TC" 1 3787
16	624661	629394	-	Target "Motif:L1_TC" 1 4823
16	624661	629394	-	Target "Motif:L1_TC" 1 4823
16	629285	629526	+	Target "Motif:L1_TC" 68 287
16	629405	632544	-	Target "Motif:L1_TC" 1662 4823
16	642623	647360	-	Target "Motif:L1_TC" 4 4821
16	642623	647360	-	Target "Motif:L1_TC" 4 4821
16	642623	647360	-	Target "Motif:L1_TC" 4 4821
16	662899	665324	+	Target "Motif:L1_TC" 2388 4826
16	662899	665324	+	Target "Motif:L1_TC" 2388 4826
16	671906	672007	+	Target "Motif:L1_TC" 3641 3745
16	1125894	1130709	-	Target "Motif:L1_TC" 1 4826
16	1125894	1130709	-	Target "Motif:L1_TC" 1 4826
16	1125894	1130709	-	Target "Motif:L1_TC" 1 4826
16	1125894	1130709	-	Target "Motif:L1_TC" 1 4826
16	1284201	1284445	-	Target "Motif:L1_TC" 1 248
18	173376	178061	-	Target "Motif:L1_TC" 1 4818
18	173376	178061	-	Target "Motif:L1_TC" 1 4818
18	173376	178061	-	Target "Motif:L1_TC" 1 4818
18	180412	183231	-	Target "Motif:L1_TC" 102 3010
18	180412	183231	-	Target "Motif:L1_TC" 102 3010
18	183227	183286	-	Target "Motif:L1_TC" 1 59
18	481591	481719	+	Target "Motif:L1_TC" 1 103
18	760375	765171	+	Target "Motif:L1_TC" 1 4823
19	4189	8851	-	Target "Motif:L1_TC" 1 4820
19	16952	21571	-	Target "Motif:L1_TC" 1 4821
19	16952	21571	-	Target "Motif:L1_TC" 1 4821
19	16952	21571	-	Target "Motif:L1_TC" 1 4821
19	36657	41356	-	Target "Motif:L1_TC" 1 4822
19	36657	41356	-	Target "Motif:L1_TC" 1 4822
19	43495	46988	-	Target "Motif:L1_TC" 1 3679
19	55134	59753	-	Target "Motif:L1_TC" 1 4822
19	55134	59753	-	Target "Motif:L1_TC" 1 4822
19	55134	59753	-	Target "Motif:L1_TC" 1 4822
19	67802	72400	-	Target "Motif:L1_TC" 1 4822

19	67802	72400	-	Target "Motif:L1_TC" 1 4822
19	67802	72400	-	Target "Motif:L1_TC" 1 4822
19	80489	85096	-	Target "Motif:L1_TC" 1 4821
19	80489	85096	-	Target "Motif:L1_TC" 1 4821
19	283395	283615	-	Target "Motif:L1_TC" 1 218
19	318551	323116	+	Target "Motif:L1_TC" 154 4828
19	323127	323346	+	Target "Motif:L1_TC" 1 218
19	323390	323750	-	Target "Motif:L1_TC" 3643 4006
19	508847	513575	+	Target "Motif:L1_TC" 1 4825
19	508847	513575	+	Target "Motif:L1_TC" 1 4825
19	508847	513575	+	Target "Motif:L1_TC" 1 4825
19	518184	518245	+	Target "Motif:L1_TC" 4742 4802
2	18185	18590	-	Target "Motif:L1_TC" 4417 4825
2	207257	207478	+	Target "Motif:L1_TC" 2 219
2	254546	254609	-	Target "Motif:L1_TC" 4 67
2	1634380	1634609	+	Target "Motif:L1_TC" 4 248
2	1634639	1638908	+	Target "Motif:L1_TC" 600 4815
2	1634639	1638908	+	Target "Motif:L1_TC" 600 4815
2	1647166	1649434	+	Target "Motif:L1_TC" 2521 4828
2	1649444	1654316	+	Target "Motif:L1_TC" 1 4827
2	1649444	1654316	+	Target "Motif:L1_TC" 1 4827
2	1654323	1658722	+	Target "Motif:L1_TC" 1 4412
2	1660334	1664858	+	Target "Motif:L1_TC" 352 4831
2	1660334	1664858	+	Target "Motif:L1_TC" 352 4831
2	1660334	1664858	+	Target "Motif:L1_TC" 352 4831
2	1664869	1665087	+	Target "Motif:L1_TC" 1 218
2	1665154	1670038	+	Target "Motif:L1_TC" 1 4831
2	1665154	1670038	+	Target "Motif:L1_TC" 1 4831
2	1665154	1670038	+	Target "Motif:L1_TC" 1 4831
2	1672216	1677104	+	Target "Motif:L1_TC" 1 4827
2	1672216	1677104	+	Target "Motif:L1_TC" 1 4827
2	1672216	1677104	+	Target "Motif:L1_TC" 1 4827
2	1677115	1678715	+	Target "Motif:L1_TC" 1 1612
2	1682670	1687524	+	Target "Motif:L1_TC" 1 4827
2	1682670	1687524	+	Target "Motif:L1_TC" 1 4827
2	1699517	1702312	+	Target "Motif:L1_TC" 1 2739
21	142595	145945	-	Target "Motif:L1_TC" 1391 4816
21	142595	145945	-	Target "Motif:L1_TC" 1391 4816
21	150340	151712	-	Target "Motif:L1_TC" 1 1390
21	447651	447871	+	Target "Motif:L1_TC" 1 218
22	5341	8708	-	Target "Motif:L1_TC" 1257 4778
22	5341	8708	-	Target "Motif:L1_TC" 1257 4778
22	84961	89578	+	Target "Motif:L1_TC" 1 4820

22	105292	109913	+	Target "Motif:L1_TC" 1 4821
22	105292	109913	+	Target "Motif:L1_TC" 1 4821
22	223249	226813	+	Target "Motif:L1_TC" 1128 4822
22	266699	271309	-	Target "Motif:L1_TC" 1 4820
22	319314	323927	+	Target "Motif:L1_TC" 1 4821
22	319314	323927	+	Target "Motif:L1_TC" 1 4821
22	328725	333337	+	Target "Motif:L1_TC" 1 4821
22	328725	333337	+	Target "Motif:L1_TC" 1 4821
22	338123	342746	+	Target "Motif:L1_TC" 1 4820
22	391706	395814	-	Target "Motif:L1_TC" 1 4315
22	539675	541024	-	Target "Motif:L1_TC" 1128 2544
22	539675	541024	-	Target "Motif:L1_TC" 1128 2544
22	575052	576371	-	Target "Motif:L1_TC" 3483 4818
22	685577	688029	-	Target "Motif:L1_TC" 1 2544
22	685577	688029	-	Target "Motif:L1_TC" 1 2544
22	688286	688334	+	Target "Motif:L1_TC" 2 49
23	5342	9855	+	Target "Motif:L1_TC" 114 4824
23	72819	77692	-	Target "Motif:L1_TC" 1 4824
23	72819	77692	-	Target "Motif:L1_TC" 1 4824
23	340583	341890	+	Target "Motif:L1_TC" 3496 4821
23	347879	349168	+	Target "Motif:L1_TC" 3496 4815
23	359895	360116	+	Target "Motif:L1_TC" 3496 3723
23	366053	367329	+	Target "Motif:L1_TC" 3496 4815
23	371685	372786	+	Target "Motif:L1_TC" 3690 4815
23	383508	384820	+	Target "Motif:L1_TC" 3496 4819
24	20026	24716	+	Target "Motif:L1_TC" 1 4815
24	157886	162585	+	Target "Motif:L1_TC" 1 4815
24	157886	162585	+	Target "Motif:L1_TC" 1 4815
24	344874	349561	-	Target "Motif:L1_TC" 1 4817
24	432306	436926	+	Target "Motif:L1_TC" 2 4815
24	475130	479820	-	Target "Motif:L1_TC" 1 4816
24	539792	542922	-	Target "Motif:L1_TC" 1669 4823
24	539792	542922	-	Target "Motif:L1_TC" 1669 4823
24	539792	542922	-	Target "Motif:L1_TC" 1669 4823
24	545992	548372	-	Target "Motif:L1_TC" 1 2320
24	548384	553263	-	Target "Motif:L1_TC" 1 4831
24	548384	553263	-	Target "Motif:L1_TC" 1 4831
24	548384	553263	-	Target "Motif:L1_TC" 1 4831
24	559615	562730	-	Target "Motif:L1_TC" 1669 4825
24	559615	562730	-	Target "Motif:L1_TC" 1669 4825
24	563518	568384	-	Target "Motif:L1_TC" 1 4822
24	568391	573275	-	Target "Motif:L1_TC" 1 4828
24	575449	578601	-	Target "Motif:L1_TC" 1663 4831

24	578592	579531	-	Target "Motif:L1_TC" 1 947
24	585884	590680	-	Target "Motif:L1_TC" 16 4829
24	585884	590680	-	Target "Motif:L1_TC" 16 4829
24	585884	590680	-	Target "Motif:L1_TC" 16 4829
24	590741	590958	-	Target "Motif:L1_TC" 1 218
24	590968	594115	-	Target "Motif:L1_TC" 1669 4831
24	597379	600795	-	Target "Motif:L1_TC" 1391 4825
24	601217	602549	-	Target "Motif:L1_TC" 48 1396
24	601217	602549	-	Target "Motif:L1_TC" 48 1396
26	534115	534333	-	Target "Motif:L1_TC" 1 219
27	68138	68373	-	Target "Motif:L1_TC" 1 240
27	174006	174410	-	Target "Motif:L1_TC" 4417 4826
27	197593	200718	-	Target "Motif:L1_TC" 1669 4824
27	205979	206386	-	Target "Motif:L1_TC" 4417 4826
27	491421	496192	-	Target "Motif:L1_TC" 1 4822
28	415941	420764	-	Target "Motif:L1_TC" 1 4831
28	415941	420764	-	Target "Motif:L1_TC" 1 4831
29	8199	8413	-	Target "Motif:L1_TC" 1 213
29	31195	31409	-	Target "Motif:L1_TC" 1 213
29	308681	309493	-	Target "Motif:L1_TC" 3994 4807
29	309489	314204	+	Target "Motif:L1_TC" 1 4807
29	309489	314204	+	Target "Motif:L1_TC" 1 4807
3	178515	178738	+	Target "Motif:L1_TC" 1 219
3	657157	661683	+	Target "Motif:L1_TC" 114 4824
3	657157	661683	+	Target "Motif:L1_TC" 114 4824
3	699983	701465	+	Target "Motif:L1_TC" 114 1620
3	701412	704448	+	Target "Motif:L1_TC" 1697 4824
3	701412	704448	+	Target "Motif:L1_TC" 1697 4824
3	705361	707811	+	Target "Motif:L1_TC" 2393 4826
3	707807	707893	+	Target "Motif:L1_TC" 160 248
3	707819	712682	+	Target "Motif:L1_TC" 1 4826
3	707819	712682	+	Target "Motif:L1_TC" 1 4826
3	712669	717511	+	Target "Motif:L1_TC" 1 4829
3	712669	717511	+	Target "Motif:L1_TC" 1 4829
3	717520	722390	+	Target "Motif:L1_TC" 1 4830
3	717520	722390	+	Target "Motif:L1_TC" 1 4830
3	717520	722390	+	Target "Motif:L1_TC" 1 4830
3	722400	727248	+	Target "Motif:L1_TC" 1 4822
3	731459	736325	+	Target "Motif:L1_TC" 1 4828
3	731459	736325	+	Target "Motif:L1_TC" 1 4828
3	736335	741210	+	Target "Motif:L1_TC" 1 4823
3	746542	746760	+	Target "Motif:L1_TC" 1 218
3	748991	750767	+	Target "Motif:L1_TC" 1 1783

3	750616	753857	+	Target "Motif:L1_TC" 1571 4826
3	750616	753857	+	Target "Motif:L1_TC" 1571 4826
3	753865	754280	+	Target "Motif:L1_TC" 1 418
3	755632	757341	+	Target "Motif:L1_TC" 3105 4830
3	763614	768449	+	Target "Motif:L1_TC" 1 4826
3	768459	769390	+	Target "Motif:L1_TC" 1 943
3	769694	772683	+	Target "Motif:L1_TC" 1669 4700
3	772684	777567	+	Target "Motif:L1_TC" 1 4831
3	772684	777567	+	Target "Motif:L1_TC" 1 4831
3	777579	777799	+	Target "Motif:L1_TC" 1 218
3	777862	782752	+	Target "Motif:L1_TC" 1 4831
3	777862	782752	+	Target "Motif:L1_TC" 1 4831
3	777862	782752	+	Target "Motif:L1_TC" 1 4831
3	784932	789360	+	Target "Motif:L1_TC" 1 4382
3	790689	795521	+	Target "Motif:L1_TC" 1 4827
3	795529	799265	+	Target "Motif:L1_TC" 1 3679
3	803751	808488	+	Target "Motif:L1_TC" 124 4821
3	814686	819554	+	Target "Motif:L1_TC" 1 4831
3	821682	823303	+	Target "Motif:L1_TC" 1 1615
3	828568	833323	+	Target "Motif:L1_TC" 124 4827
3	828568	833323	+	Target "Motif:L1_TC" 124 4827
3	828568	833323	+	Target "Motif:L1_TC" 124 4827
3	843872	845498	+	Target "Motif:L1_TC" 1 1639
3	843872	845498	+	Target "Motif:L1_TC" 1 1639
3	845475	849354	+	Target "Motif:L1_TC" 988 4828
3	845475	849354	+	Target "Motif:L1_TC" 988 4828
3	853553	858438	+	Target "Motif:L1_TC" 1 4830
3	853553	858438	+	Target "Motif:L1_TC" 1 4830
3	853553	858438	+	Target "Motif:L1_TC" 1 4830
3	853553	858438	+	Target "Motif:L1_TC" 1 4830
3	860615	865052	+	Target "Motif:L1_TC" 1 4382
3	860615	865052	+	Target "Motif:L1_TC" 1 4382
3	872316	877200	+	Target "Motif:L1_TC" 1 4831
3	872316	877200	+	Target "Motif:L1_TC" 1 4831
3	877213	877433	+	Target "Motif:L1_TC" 1 218
3	879658	879877	+	Target "Motif:L1_TC" 1 218
3	880761	882870	+	Target "Motif:L1_TC" 2710 4829
3	882880	887769	+	Target "Motif:L1_TC" 1 4827
3	882880	887769	+	Target "Motif:L1_TC" 1 4827
3	882880	887769	+	Target "Motif:L1_TC" 1 4827
3	882880	887769	+	Target "Motif:L1_TC" 1 4827
3	887756	887852	+	Target "Motif:L1_TC" 160 248
3	887777	892659	+	Target "Motif:L1_TC" 1 4829

3	887777	892659	+	Target "Motif:L1_TC" 1 4829
3	887777	892659	+	Target "Motif:L1_TC" 1 4829
3	887777	892659	+	Target "Motif:L1_TC" 1 4829
3	892669	892889	+	Target "Motif:L1_TC" 1 218
3	892950	897819	+	Target "Motif:L1_TC" 1 4831
3	892950	897819	+	Target "Motif:L1_TC" 1 4831
3	892950	897819	+	Target "Motif:L1_TC" 1 4831
3	892950	897819	+	Target "Motif:L1_TC" 1 4831
3	897815	898047	+	Target "Motif:L1_TC" 1 245
3	898107	902978	+	Target "Motif:L1_TC" 1 4831
3	898107	902978	+	Target "Motif:L1_TC" 1 4831
3	905143	907928	+	Target "Motif:L1_TC" 1 2739
3	914728	919560	+	Target "Motif:L1_TC" 1 4831
3	914728	919560	+	Target "Motif:L1_TC" 1 4831
3	921724	922024	+	Target "Motif:L1_TC" 1 300
3	927498	931990	+	Target "Motif:L1_TC" 1 4458
3	934022	934265	+	Target "Motif:L1_TC" 4584 4827
3	936258	941062	+	Target "Motif:L1_TC" 1 4798
3	942995	946305	+	Target "Motif:L1_TC" 1436 4828
3	942995	946305	+	Target "Motif:L1_TC" 1436 4828
3	946313	951195	+	Target "Motif:L1_TC" 1 4831
3	951204	951401	+	Target "Motif:L1_TC" 1 198
3	951460	956268	+	Target "Motif:L1_TC" 1 4819
3	951460	956268	+	Target "Motif:L1_TC" 1 4819
3	1103263	1103350	-	Target "Motif:L1_TC" 2080 2166
3	1103488	1104384	+	Target "Motif:L1_TC" 1201 2166
3	1155528	1155624	+	Target "Motif:L1_TC" 11 220
3	1155620	1155663	+	Target "Motif:L1_TC" 4777 4819
31	157083	159605	+	Target "Motif:L1_TC" 1 2544
31	157083	159605	+	Target "Motif:L1_TC" 1 2544
31	460365	464350	+	Target "Motif:L1_TC" 750 4818
31	460365	464350	+	Target "Motif:L1_TC" 750 4818
32	14394	19058	+	Target "Motif:L1_TC" 31 4825
32	25558	25663	+	Target "Motif:L1_TC" 3641 3745
32	653743	658337	+	Target "Motif:L1_TC" 106 4816
32	653743	658337	+	Target "Motif:L1_TC" 106 4816
32	653743	658337	+	Target "Motif:L1_TC" 106 4816
32	660473	665083	+	Target "Motif:L1_TC" 106 4816
32	660473	665083	+	Target "Motif:L1_TC" 106 4816
32	816289	816507	+	Target "Motif:L1_TC" 1 218
32	977807	982522	+	Target "Motif:L1_TC" 1 4820
32	995399	996578	-	Target "Motif:L1_TC" 3641 4825
33	25832	30453	+	Target "Motif:L1_TC" 1 4821

33	25832	30453	+	Target "Motif:L1_TC" 1 4821
33	50759	55379	+	Target "Motif:L1_TC" 1 4821
33	67969	72615	+	Target "Motif:L1_TC" 1 4820
33	67969	72615	+	Target "Motif:L1_TC" 1 4820
33	67969	72615	+	Target "Motif:L1_TC" 1 4820
33	127272	131973	+	Target "Motif:L1_TC" 1 4821
35	8413	9719	-	Target "Motif:L1_TC" 3496 4827
35	8413	9719	-	Target "Motif:L1_TC" 3496 4827
35	16162	17467	-	Target "Motif:L1_TC" 3496 4826
35	16162	17467	-	Target "Motif:L1_TC" 3496 4826
35	38856	40175	-	Target "Motif:L1_TC" 3481 4815
35	69093	70657	+	Target "Motif:L1_TC" 1 1600
35	70669	73667	+	Target "Motif:L1_TC" 1697 4822
35	88678	90266	+	Target "Motif:L1_TC" 1 1620
35	90215	93299	+	Target "Motif:L1_TC" 1697 4824
35	99531	102712	+	Target "Motif:L1_TC" 1595 4826
35	102723	107521	+	Target "Motif:L1_TC" 1 4826
35	107530	108552	+	Target "Motif:L1_TC" 1 1040
35	114144	118974	+	Target "Motif:L1_TC" 1 4824
35	114144	118974	+	Target "Motif:L1_TC" 1 4824
35	118970	121335	+	Target "Motif:L1_TC" 1 2320
35	131569	136445	+	Target "Motif:L1_TC" 1 4823
35	136456	141188	+	Target "Motif:L1_TC" 1 4822
35	136456	141188	+	Target "Motif:L1_TC" 1 4822
35	141199	142804	+	Target "Motif:L1_TC" 1 1615
35	144170	149019	+	Target "Motif:L1_TC" 1 4817
35	144170	149019	+	Target "Motif:L1_TC" 1 4817
35	162718	167507	+	Target "Motif:L1_TC" 1 4828
35	162718	167507	+	Target "Motif:L1_TC" 1 4828
35	162718	167507	+	Target "Motif:L1_TC" 1 4828
35	162718	167507	+	Target "Motif:L1_TC" 1 4828
35	382772	382991	+	Target "Motif:L1_TC" 1 217
35	391465	391684	+	Target "Motif:L1_TC" 1 217
35	400792	401010	-	Target "Motif:L1_TC" 1 217
35	506863	507081	+	Target "Motif:L1_TC" 1 217
35	516197	516416	-	Target "Motif:L1_TC" 1 217
36	418078	418297	+	Target "Motif:L1_TC" 1 218
37	263280	263394	-	Target "Motif:L1_TC" 1 222
37	263390	268286	-	Target "Motif:L1_TC" 1 4825
37	263390	268286	-	Target "Motif:L1_TC" 1 4825
37	281868	282116	+	Target "Motif:L1_TC" 1 218
38	425223	429523	-	Target "Motif:L1_TC" 2 4423
38	425223	429523	-	Target "Motif:L1_TC" 2 4423

39	144858	146208	-	Target "Motif:L1_TC" 3449 4807
4	4137	8861	-	Target "Motif:L1_TC" 1 4815
4	730937	735710	-	Target "Motif:L1_TC" 1 4814
4	730937	735710	-	Target "Motif:L1_TC" 1 4814
4	743208	747909	-	Target "Motif:L1_TC" 1 4815
4	743208	747909	-	Target "Motif:L1_TC" 1 4815
4	753057	757766	+	Target "Motif:L1_TC" 1 4815
4	1009010	1009951	-	Target "Motif:L1_TC" 1 943
4	1455404	1460270	+	Target "Motif:L1_TC" 3 4819
4	1455404	1460270	+	Target "Motif:L1_TC" 3 4819
4	1455404	1460270	+	Target "Motif:L1_TC" 3 4819
40	80662	85338	-	Target "Motif:L1_TC" 99 4821
40	281569	283046	+	Target "Motif:L1_TC" 99 1612
40	311101	315389	-	Target "Motif:L1_TC" 455 4820
40	311101	315389	-	Target "Motif:L1_TC" 455 4820
40	337493	342193	+	Target "Motif:L1_TC" 86 4821
40	337493	342193	+	Target "Motif:L1_TC" 86 4821
40	337493	342193	+	Target "Motif:L1_TC" 86 4821
41	414408	414614	-	Target "Motif:L1_TC" 1 213
42	6478	10991	+	Target "Motif:L1_TC" 114 4824
42	6478	10991	+	Target "Motif:L1_TC" 114 4824
42	16648	19758	+	Target "Motif:L1_TC" 1667 4824
42	43703	48325	-	Target "Motif:L1_TC" 1 4822
42	55733	58770	-	Target "Motif:L1_TC" 1727 4798
42	58744	60316	-	Target "Motif:L1_TC" 1 1620
42	67640	71961	-	Target "Motif:L1_TC" 1 4382
42	67640	71961	-	Target "Motif:L1_TC" 1 4382
42	76734	80957	-	Target "Motif:L1_TC" 1 4423
42	76734	80957	-	Target "Motif:L1_TC" 1 4423
42	76734	80957	-	Target "Motif:L1_TC" 1 4423
42	161677	164881	-	Target "Motif:L1_TC" 1588 4829
42	161677	164881	-	Target "Motif:L1_TC" 1588 4829
42	199328	203354	-	Target "Motif:L1_TC" 750 4817
42	199328	203354	-	Target "Motif:L1_TC" 750 4817
42	199328	203354	-	Target "Motif:L1_TC" 750 4817
42	438675	438895	-	Target "Motif:L1_TC" 1 218
42	438906	443751	-	Target "Motif:L1_TC" 4 4821
42	438906	443751	-	Target "Motif:L1_TC" 4 4821
42	438906	443751	-	Target "Motif:L1_TC" 4 4821
42	507107	507326	+	Target "Motif:L1_TC" 1 218
42	621965	622189	+	Target "Motif:L1_TC" 1 248
42	669976	674302	-	Target "Motif:L1_TC" 4 4382
42	687634	687871	+	Target "Motif:L1_TC" 1 248

42	802585	807313	-	Target "Motif:L1_TC" 124 4827
42	802585	807313	-	Target "Motif:L1_TC" 124 4827
42	845069	849811	-	Target "Motif:L1_TC" 124 4820
42	845069	849811	-	Target "Motif:L1_TC" 124 4820
43	112683	113863	+	Target "Motif:L1_TC" 3641 4826
44	11191	15937	-	Target "Motif:L1_TC" 1 4828
44	11191	15937	-	Target "Motif:L1_TC" 1 4828
44	148976	153709	-	Target "Motif:L1_TC" 1 4817
45	19546	19765	+	Target "Motif:L1_TC" 1 219
45	153434	156678	-	Target "Motif:L1_TC" 1571 4831
45	153434	156678	-	Target "Motif:L1_TC" 1571 4831
45	156533	158284	-	Target "Motif:L1_TC" 7 1783
45	158294	159226	-	Target "Motif:L1_TC" 1 943
45	271369	276166	-	Target "Motif:L1_TC" 1 4819
45	271369	276166	-	Target "Motif:L1_TC" 1 4819
45	271369	276166	-	Target "Motif:L1_TC" 1 4819
45	297245	302117	+	Target "Motif:L1_TC" 1 4831
45	297245	302117	+	Target "Motif:L1_TC" 1 4831
45	297245	302117	+	Target "Motif:L1_TC" 1 4831
45	349813	353137	-	Target "Motif:L1_TC" 1336 4825
45	365055	368379	-	Target "Motif:L1_TC" 1336 4826
45	375940	379252	-	Target "Motif:L1_TC" 1336 4825
45	383954	387291	-	Target "Motif:L1_TC" 1336 4824
45	394895	398227	-	Target "Motif:L1_TC" 1336 4825
45	410871	414172	-	Target "Motif:L1_TC" 1336 4824
45	421768	424851	-	Target "Motif:L1_TC" 1578 4825
46	28735	30045	+	Target "Motif:L1_TC" 3496 4824
46	127592	128899	-	Target "Motif:L1_TC" 3496 4822
46	127592	128899	-	Target "Motif:L1_TC" 3496 4822
46	127592	128899	-	Target "Motif:L1_TC" 3496 4822
46	127592	128899	-	Target "Motif:L1_TC" 3496 4822
46	138808	140122	-	Target "Motif:L1_TC" 3496 4824
46	220721	224096	+	Target "Motif:L1_TC" 1257 4778
46	220721	224096	+	Target "Motif:L1_TC" 1257 4778
46	220721	224096	+	Target "Motif:L1_TC" 1257 4778
46	252644	252734	-	Target "Motif:L1_TC" 1 89
46	286382	289065	+	Target "Motif:L1_TC" 2140 4831
5	516602	517784	+	Target "Motif:L1_TC" 3641 4825
5	744146	744368	+	Target "Motif:L1_TC" 1 218
5	757517	762228	-	Target "Motif:L1_TC" 2 4823
5	819290	822289	-	Target "Motif:L1_TC" 2 3103
5	1088172	1088434	-	Target "Motif:L1_TC" 1 248
5	1166995	1167061	+	Target "Motif:L1_TC" 1 67

5	1228055	1232516	+	Target "Motif:L1_TC" 1 4423
5	1228055	1232516	+	Target "Motif:L1_TC" 1 4423
5	1228055	1232516	+	Target "Motif:L1_TC" 1 4423
5	1228055	1232516	+	Target "Motif:L1_TC" 1 4423
5	1245678	1250436	+	Target "Motif:L1_TC" 124 4828
5	1245678	1250436	+	Target "Motif:L1_TC" 124 4828
5	1262693	1267586	+	Target "Motif:L1_TC" 1 4831
5	1273810	1278688	+	Target "Motif:L1_TC" 1 4827
5	1273810	1278688	+	Target "Motif:L1_TC" 1 4827
5	1273810	1278688	+	Target "Motif:L1_TC" 1 4827
5	1273810	1278688	+	Target "Motif:L1_TC" 1 4827
5	1294438	1299328	+	Target "Motif:L1_TC" 1 4830
5	1294438	1299328	+	Target "Motif:L1_TC" 1 4830
5	1303828	1305419	+	Target "Motif:L1_TC" 3234 4830
5	1305429	1310319	+	Target "Motif:L1_TC" 1 4831
5	1314508	1317307	+	Target "Motif:L1_TC" 1 2739
5	1314508	1317307	+	Target "Motif:L1_TC" 1 2739
5	1322854	1327733	+	Target "Motif:L1_TC" 1 4829
5	1331913	1336795	+	Target "Motif:L1_TC" 1 4830
5	1331913	1336795	+	Target "Motif:L1_TC" 1 4830
5	1338968	1339318	+	Target "Motif:L1_TC" 1 350
5	1339373	1339427	+	Target "Motif:L1_TC" 4778 4831
5	1339437	1344313	+	Target "Motif:L1_TC" 1 4828
5	1339437	1344313	+	Target "Motif:L1_TC" 1 4828
5	1339437	1344313	+	Target "Motif:L1_TC" 1 4828
5	1344323	1349210	+	Target "Motif:L1_TC" 1 4827
5	1344323	1349210	+	Target "Motif:L1_TC" 1 4827
5	1344323	1349210	+	Target "Motif:L1_TC" 1 4827
5	1349220	1354076	+	Target "Motif:L1_TC" 1 4818
5	1349220	1354076	+	Target "Motif:L1_TC" 1 4818
5	1356612	1361063	+	Target "Motif:L1_TC" 416 4826
5	1356612	1361063	+	Target "Motif:L1_TC" 416 4826
5	1361074	1365932	+	Target "Motif:L1_TC" 1 4829
5	1361074	1365932	+	Target "Motif:L1_TC" 1 4829
5	1365941	1370790	+	Target "Motif:L1_TC" 1 4819
5	1365941	1370790	+	Target "Motif:L1_TC" 1 4819
6	272638	273936	+	Target "Motif:L1_TC" 3483 4815
6	284112	288737	+	Target "Motif:L1_TC" 1 4814
6	284112	288737	+	Target "Motif:L1_TC" 1 4814
6	284112	288737	+	Target "Motif:L1_TC" 1 4814
6	328919	329194	-	Target "Motif:L1_TC" 4546 4820
6	329186	329551	-	Target "Motif:L1_TC" 3655 4049
6	334292	334565	-	Target "Motif:L1_TC" 4546 4819

6	334557	338202	-	Target "Motif:L1_TC" 180 4049
6	364132	364406	-	Target "Motif:L1_TC" 4546 4820
6	364398	368049	-	Target "Motif:L1_TC" 165 4049
6	516034	516162	-	Target "Motif:L1_TC" 3305 3432
6	893796	898559	-	Target "Motif:L1_TC" 1 4815
6	903701	908474	+	Target "Motif:L1_TC" 1 4816
6	1171616	1174087	+	Target "Motif:L1_TC" 2340 4831
7	13044	13108	-	Target "Motif:L1_TC" 4 67
7	627403	631188	-	Target "Motif:L1_TC" 945 4822
7	627403	631188	-	Target "Motif:L1_TC" 945 4822
7	631409	635133	-	Target "Motif:L1_TC" 1 3679
7	635141	640028	-	Target "Motif:L1_TC" 1 4829
7	635141	640028	-	Target "Motif:L1_TC" 1 4829
7	640037	644938	-	Target "Motif:L1_TC" 1 4828
7	640037	644938	-	Target "Motif:L1_TC" 1 4828
7	640037	644938	-	Target "Motif:L1_TC" 1 4828
7	644925	648058	-	Target "Motif:L1_TC" 1669 4829
7	650107	653273	-	Target "Motif:L1_TC" 1 3103
7	650107	653273	-	Target "Motif:L1_TC" 1 3103
7	653282	658167	-	Target "Motif:L1_TC" 1 4830
7	653282	658167	-	Target "Motif:L1_TC" 1 4830
7	658177	661420	-	Target "Motif:L1_TC" 1585 4829
7	658177	661420	-	Target "Motif:L1_TC" 1585 4829
7	661284	663059	-	Target "Motif:L1_TC" 1 1783
7	663069	664644	-	Target "Motif:L1_TC" 3234 4828
7	667546	668500	-	Target "Motif:L1_TC" 1 961
7	674700	679581	-	Target "Motif:L1_TC" 1 4831
7	674700	679581	-	Target "Motif:L1_TC" 1 4831
7	681754	683873	-	Target "Motif:L1_TC" 2700 4831
7	685220	689649	-	Target "Motif:L1_TC" 1 4382
7	685220	689649	-	Target "Motif:L1_TC" 1 4382
7	689658	694534	-	Target "Motif:L1_TC" 1 4830
7	689658	694534	-	Target "Motif:L1_TC" 1 4830
7	689658	694534	-	Target "Motif:L1_TC" 1 4830
7	694544	699410	-	Target "Motif:L1_TC" 1 4830
7	694544	699410	-	Target "Motif:L1_TC" 1 4830
7	699419	704261	-	Target "Motif:L1_TC" 1 4829
7	704248	708959	-	Target "Motif:L1_TC" 1 4829
7	715320	720206	-	Target "Motif:L1_TC" 1 4829
7	722212	725447	-	Target "Motif:L1_TC" 1571 4831
7	725297	727061	-	Target "Motif:L1_TC" 1 1783
7	727069	728200	-	Target "Motif:L1_TC" 3681 4831
7	728782	729197	-	Target "Motif:L1_TC" 1 418

7	731216	736097	-	Target "Motif:L1_TC" 1 4830
7	731216	736097	-	Target "Motif:L1_TC" 1 4830
7	731216	736097	-	Target "Motif:L1_TC" 1 4830
7	736108	740985	-	Target "Motif:L1_TC" 1 4830
7	744076	748523	-	Target "Motif:L1_TC" 1 4392
7	744076	748523	-	Target "Motif:L1_TC" 1 4392
7	748534	753434	-	Target "Motif:L1_TC" 1 4828
7	748534	753434	-	Target "Motif:L1_TC" 1 4828
7	748534	753434	-	Target "Motif:L1_TC" 1 4828
7	753421	757212	-	Target "Motif:L1_TC" 1083 4828
7	753421	757212	-	Target "Motif:L1_TC" 1083 4828
7	758896	761697	-	Target "Motif:L1_TC" 1 2739
7	761706	766586	-	Target "Motif:L1_TC" 1 4829
7	761706	766586	-	Target "Motif:L1_TC" 1 4829
7	761706	766586	-	Target "Motif:L1_TC" 1 4829
7	768763	772530	-	Target "Motif:L1_TC" 990 4831
7	768763	772530	-	Target "Motif:L1_TC" 990 4831
7	768763	772530	-	Target "Motif:L1_TC" 990 4831
7	774121	778996	-	Target "Motif:L1_TC" 1 4825
7	774121	778996	-	Target "Motif:L1_TC" 1 4825
7	779004	783883	-	Target "Motif:L1_TC" 1 4828
7	786103	786319	-	Target "Motif:L1_TC" 1 218
7	793429	795029	-	Target "Motif:L1_TC" 1 1615
7	793429	795029	-	Target "Motif:L1_TC" 1 1615
7	797269	797489	-	Target "Motif:L1_TC" 1 218
7	801724	801939	-	Target "Motif:L1_TC" 1 218
7	803997	804217	-	Target "Motif:L1_TC" 1 218
7	806436	806651	-	Target "Motif:L1_TC" 2 218
7	806663	809812	-	Target "Motif:L1_TC" 1669 4831
7	806663	809812	-	Target "Motif:L1_TC" 1669 4831
7	810364	814809	-	Target "Motif:L1_TC" 1 4423
7	810364	814809	-	Target "Motif:L1_TC" 1 4423
7	818987	823437	-	Target "Motif:L1_TC" 420 4830
7	818987	823437	-	Target "Motif:L1_TC" 420 4830
7	818987	823437	-	Target "Motif:L1_TC" 420 4830
7	824732	828308	-	Target "Motif:L1_TC" 1 3662
7	828319	833038	-	Target "Motif:L1_TC" 1 4824
7	828319	833038	-	Target "Motif:L1_TC" 1 4824
7	833049	837783	-	Target "Motif:L1_TC" 1 4823
7	833049	837783	-	Target "Motif:L1_TC" 1 4823
7	833049	837783	-	Target "Motif:L1_TC" 1 4823
7	837794	842472	-	Target "Motif:L1_TC" 57 4823
7	837794	842472	-	Target "Motif:L1_TC" 57 4823

7	837794	842472	-	Target "Motif:L1_TC" 57 4823
7	842473	845976	-	Target "Motif:L1_TC" 1 3575
7	842473	845976	-	Target "Motif:L1_TC" 1 3575
7	842473	845976	-	Target "Motif:L1_TC" 1 3575
7	845987	850725	-	Target "Motif:L1_TC" 1 4823
7	845987	850725	-	Target "Motif:L1_TC" 1 4823
7	850735	855476	-	Target "Motif:L1_TC" 1 4823
7	850735	855476	-	Target "Motif:L1_TC" 1 4823
7	850735	855476	-	Target "Motif:L1_TC" 1 4823
7	855487	858504	-	Target "Motif:L1_TC" 1768 4823
7	860159	864599	-	Target "Motif:L1_TC" 1 4382
7	860159	864599	-	Target "Motif:L1_TC" 1 4382
7	864609	869493	-	Target "Motif:L1_TC" 1 4829
7	864609	869493	-	Target "Motif:L1_TC" 1 4829
7	864609	869493	-	Target "Motif:L1_TC" 1 4829
7	873195	874135	-	Target "Motif:L1_TC" 1 943
7	876303	881207	-	Target "Motif:L1_TC" 1 4827
7	876303	881207	-	Target "Motif:L1_TC" 1 4827
7	881194	886066	-	Target "Motif:L1_TC" 1 4826
7	892383	895535	-	Target "Motif:L1_TC" 1 3103
7	892383	895535	-	Target "Motif:L1_TC" 1 3103
7	1269168	1270710	+	Target "Motif:L1_TC" 180 1881
7	1276252	1279924	+	Target "Motif:L1_TC" 180 4049
7	1279916	1280191	+	Target "Motif:L1_TC" 4546 4822
7	1285575	1288909	+	Target "Motif:L1_TC" 180 3694
7	1290332	1293984	+	Target "Motif:L1_TC" 180 4049
7	1293976	1294253	+	Target "Motif:L1_TC" 4546 4822
7	1293976	1294253	+	Target "Motif:L1_TC" 4546 4822
7	1299606	1303271	+	Target "Motif:L1_TC" 180 4049
7	1303263	1303537	+	Target "Motif:L1_TC" 4546 4821
7	1313033	1313342	+	Target "Motif:L1_TC" 180 488
7	1330271	1333939	+	Target "Motif:L1_TC" 180 4049
7	1333931	1334204	+	Target "Motif:L1_TC" 4546 4822
7	1333931	1334204	+	Target "Motif:L1_TC" 4546 4822
7	1333931	1334204	+	Target "Motif:L1_TC" 4546 4822
7	1333931	1334204	+	Target "Motif:L1_TC" 4546 4822
7	1338107	1338294	+	Target "Motif:L1_TC" 4635 4821
7	1343659	1347325	+	Target "Motif:L1_TC" 180 4049
7	1347317	1347589	+	Target "Motif:L1_TC" 4546 4822
7	1347317	1347589	+	Target "Motif:L1_TC" 4546 4822
7	1355775	1359370	+	Target "Motif:L1_TC" 180 4049
7	1359362	1359636	+	Target "Motif:L1_TC" 4546 4820
7	1366188	1366552	+	Target "Motif:L1_TC" 3655 4049

7	1366544	1366818	+	Target "Motif:L1_TC" 4546 4821
7	1373260	1373620	+	Target "Motif:L1_TC" 3655 4049
7	1373612	1373886	+	Target "Motif:L1_TC" 4546 4820
7	1460069	1464689	+	Target "Motif:L1_TC" 1 4820
7	1584782	1589393	-	Target "Motif:L1_TC" 1 4821
7	1691667	1696295	+	Target "Motif:L1_TC" 1 4821
7	1691667	1696295	+	Target "Motif:L1_TC" 1 4821
7	1757325	1761944	+	Target "Motif:L1_TC" 1 4821
7	1757325	1761944	+	Target "Motif:L1_TC" 1 4821
7	1771182	1772497	-	Target "Motif:L1_TC" 3496 4824
7	1803016	1807651	+	Target "Motif:L1_TC" 1 4820
7	1812464	1813406	+	Target "Motif:L1_TC" 1 943
7	1818496	1823115	+	Target "Motif:L1_TC" 1 4821
7	1837700	1842306	+	Target "Motif:L1_TC" 1 4820
7	1847127	1851749	+	Target "Motif:L1_TC" 1 4820
7	1914795	1918554	+	Target "Motif:L1_TC" 990 4824
7	1914795	1918554	+	Target "Motif:L1_TC" 990 4824
7	1918565	1923283	+	Target "Motif:L1_TC" 1 4823
7	1923294	1928031	+	Target "Motif:L1_TC" 1 4823
7	1923294	1928031	+	Target "Motif:L1_TC" 1 4823
7	1928042	1932764	+	Target "Motif:L1_TC" 1 4822
7	1928042	1932764	+	Target "Motif:L1_TC" 1 4822
7	1932775	1935016	+	Target "Motif:L1_TC" 1 2320
7	1941727	1945598	+	Target "Motif:L1_TC" 924 4823
7	1945609	1950347	+	Target "Motif:L1_TC" 1 4823
7	1945609	1950347	+	Target "Motif:L1_TC" 1 4823
7	1945609	1950347	+	Target "Motif:L1_TC" 1 4823
7	1950358	1955085	+	Target "Motif:L1_TC" 1 4823
7	1950358	1955085	+	Target "Motif:L1_TC" 1 4823
7	1955095	1959819	+	Target "Motif:L1_TC" 1 4822
7	1955095	1959819	+	Target "Motif:L1_TC" 1 4822
7	1959829	1962497	+	Target "Motif:L1_TC" 1 2745
7	1959829	1962497	+	Target "Motif:L1_TC" 1 2745
7	1959829	1962497	+	Target "Motif:L1_TC" 1 2745
7	1962779	1963909	+	Target "Motif:L1_TC" 3681 4824
7	1963896	1963993	+	Target "Motif:L1_TC" 160 248
7	1963918	1968801	+	Target "Motif:L1_TC" 1 4831
7	1963918	1968801	+	Target "Motif:L1_TC" 1 4831
7	1963918	1968801	+	Target "Motif:L1_TC" 1 4831
7	1968807	1973690	+	Target "Motif:L1_TC" 1 4828
7	1968807	1973690	+	Target "Motif:L1_TC" 1 4828
7	1968807	1973690	+	Target "Motif:L1_TC" 1 4828
7	1968807	1973690	+	Target "Motif:L1_TC" 1 4828

7	1968807	1973690	+	Target "Motif:L1_TC" 1 4828
7	1973698	1977789	+	Target "Motif:L1_TC" 1 4031
7	1979370	1979478	+	Target "Motif:L1_TC" 420 527
7	1979474	1983576	+	Target "Motif:L1_TC" 625 4823
7	1979474	1983576	+	Target "Motif:L1_TC" 625 4823
7	1983587	1988329	+	Target "Motif:L1_TC" 1 4823
7	1983587	1988329	+	Target "Motif:L1_TC" 1 4823
7	1983587	1988329	+	Target "Motif:L1_TC" 1 4823
7	1983587	1988329	+	Target "Motif:L1_TC" 1 4823
7	1988340	1993082	+	Target "Motif:L1_TC" 1 4823
7	1988340	1993082	+	Target "Motif:L1_TC" 1 4823
7	1993093	1997833	+	Target "Motif:L1_TC" 1 4822
7	1993093	1997833	+	Target "Motif:L1_TC" 1 4822
7	1997844	1999664	+	Target "Motif:L1_TC" 1 1897
7	2002761	2007648	+	Target "Motif:L1_TC" 1 4829
7	2002761	2007648	+	Target "Motif:L1_TC" 1 4829
7	2002761	2007648	+	Target "Motif:L1_TC" 1 4829
7	2007659	2012538	+	Target "Motif:L1_TC" 1 4825
7	2007659	2012538	+	Target "Motif:L1_TC" 1 4825
7	2007659	2012538	+	Target "Motif:L1_TC" 1 4825
7	2007659	2012538	+	Target "Motif:L1_TC" 1 4825
7	2012548	2016888	+	Target "Motif:L1_TC" 1 4322
7	2012548	2016888	+	Target "Motif:L1_TC" 1 4322
7	2017617	2021375	+	Target "Motif:L1_TC" 990 4828
7	2017617	2021375	+	Target "Motif:L1_TC" 990 4828
7	2017617	2021375	+	Target "Motif:L1_TC" 990 4828
7	2021378	2026219	+	Target "Motif:L1_TC" 1 4817
7	2053293	2057974	+	Target "Motif:L1_TC" 1 4815
7	2053293	2057974	+	Target "Motif:L1_TC" 1 4815
7	2066411	2066630	+	Target "Motif:L1_TC" 1 218
7	2068672	2068888	+	Target "Motif:L1_TC" 1 218
7	2073330	2075280	+	Target "Motif:L1_TC" 2825 4823
7	2075291	2079988	+	Target "Motif:L1_TC" 1 4823
7	2075291	2079988	+	Target "Motif:L1_TC" 1 4823
7	2079999	2082076	+	Target "Motif:L1_TC" 1 2168
7	2084147	2088799	+	Target "Motif:L1_TC" 26 4815
8	321774	322880	+	Target "Motif:L1_TC" 3698 4820
8	710680	715364	-	Target "Motif:L1_TC" 1 4817
8	710680	715364	-	Target "Motif:L1_TC" 1 4817
8	710680	715364	-	Target "Motif:L1_TC" 1 4817
8	789004	789066	-	Target "Motif:L1_TC" 6 67
8	1059718	1064520	-	Target "Motif:L1_TC" 1 4822
8	1059718	1064520	-	Target "Motif:L1_TC" 1 4822

8	1073395	1078100	-	Target "Motif:L1_TC" 1 4817
8	1086244	1090947	-	Target "Motif:L1_TC" 1 4816
8	1086244	1090947	-	Target "Motif:L1_TC" 1 4816
8	1097474	1098652	-	Target "Motif:L1_TC" 1 1195
8	1098661	1103452	-	Target "Motif:L1_TC" 1 4825
8	1098661	1103452	-	Target "Motif:L1_TC" 1 4825
8	1103460	1107887	-	Target "Motif:L1_TC" 374 4825
8	1115150	1115513	+	Target "Motif:L1_TC" 3643 4006
8	1115557	1115776	-	Target "Motif:L1_TC" 1 218
8	1115787	1120343	-	Target "Motif:L1_TC" 154 4827
8	1115787	1120343	-	Target "Motif:L1_TC" 154 4827
8	1115787	1120343	-	Target "Motif:L1_TC" 154 4827
8	1115787	1120343	-	Target "Motif:L1_TC" 154 4827
8	1151506	1151726	+	Target "Motif:L1_TC" 1 218
