# Supplementary figures and images for: Repeat-Driven Generation of Antigenic Diversity in a Major Human Pathogen, Trypanosoma cruzi
Source: Front Cell Infect Microbiol. 2021 Mar 3;11:614665. doi: 10.3389/fcimb.2021.614665 (PMC7966520; doi:10.3389/fcimb.2021.614665)

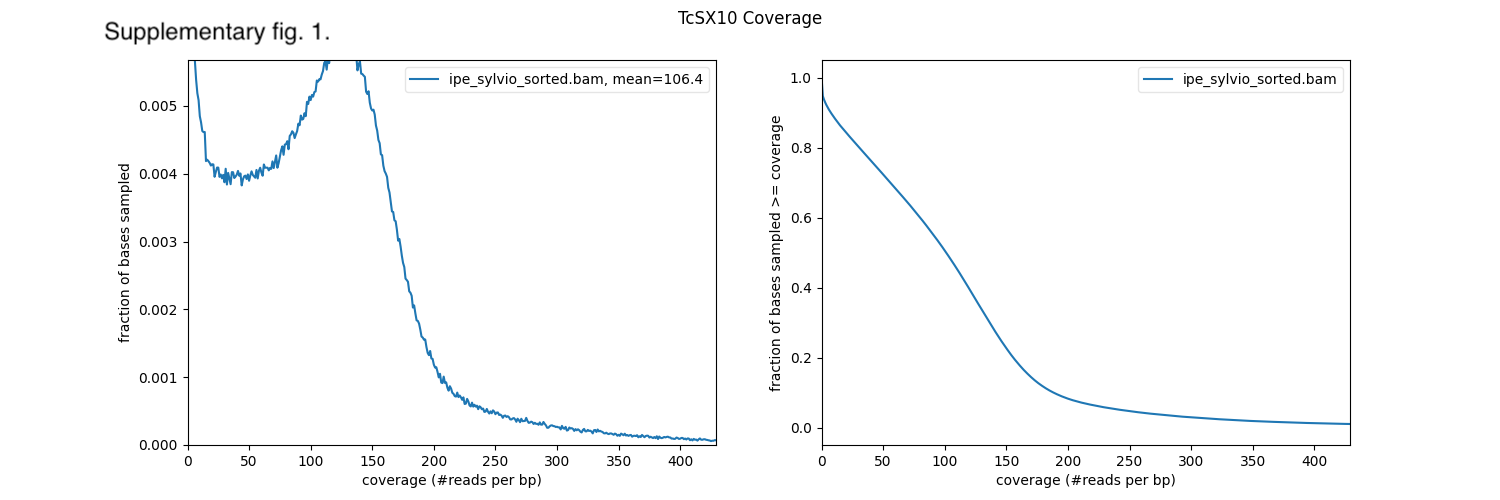

Supplement: Supplementary Figure 1 — Results from mapping Illumina reads back to the completed genome sequence. The graphs show the overall coverage across the genome. [file Image_1.png]

Supplementary fig. 2.

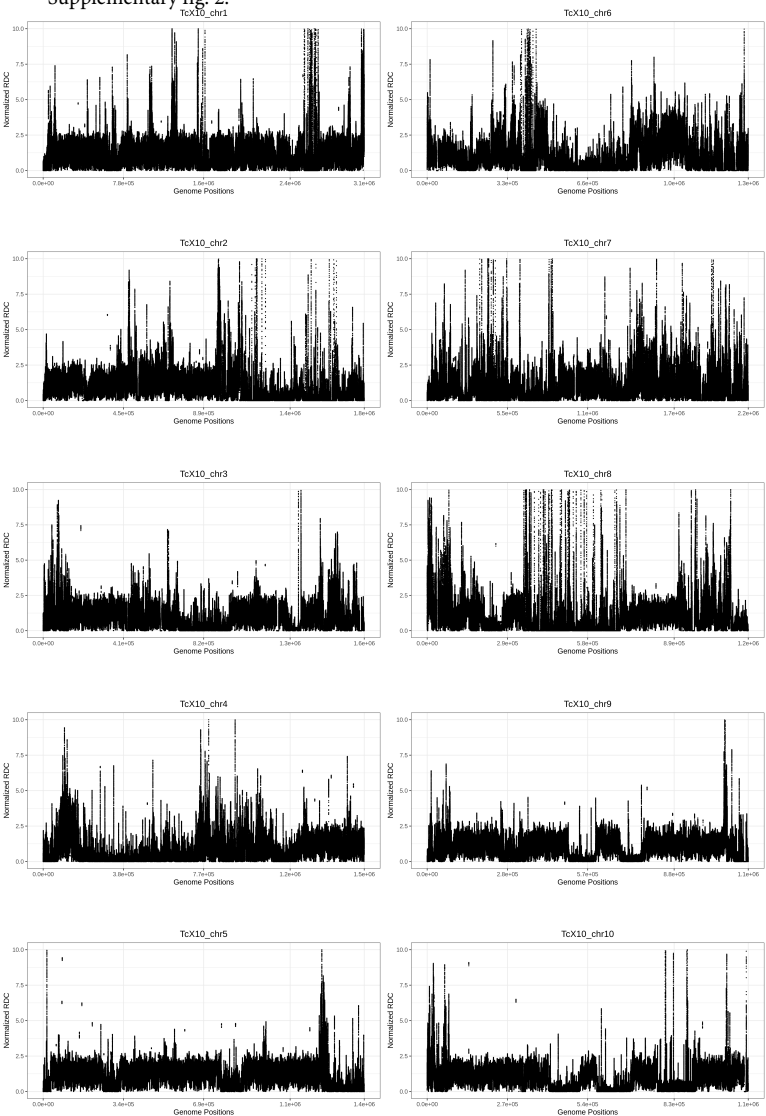

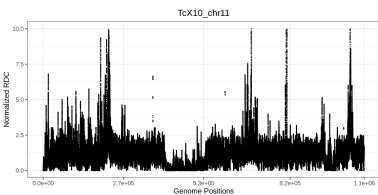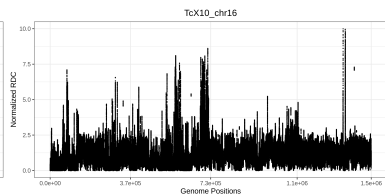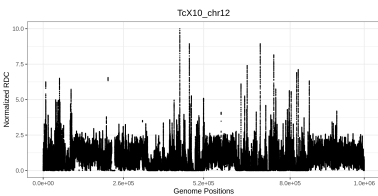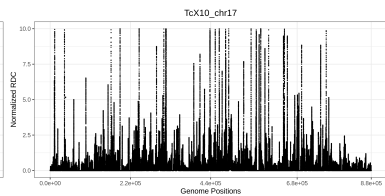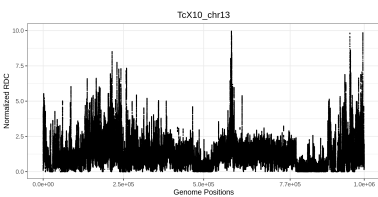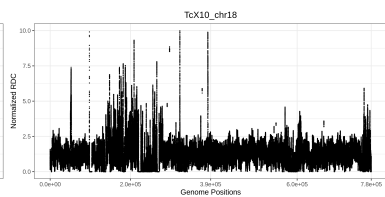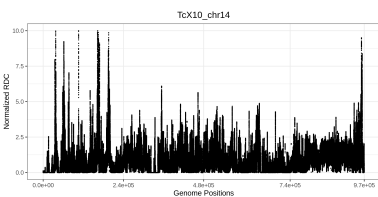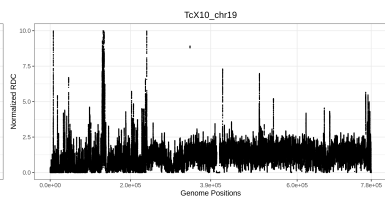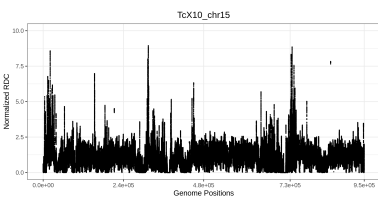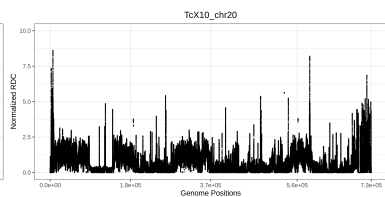

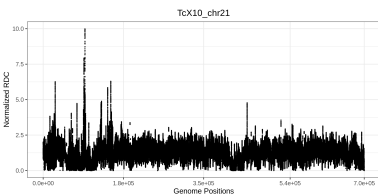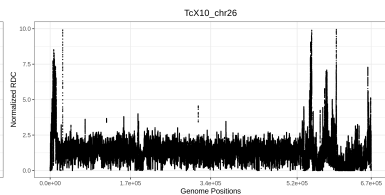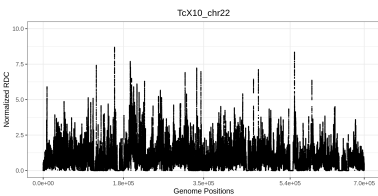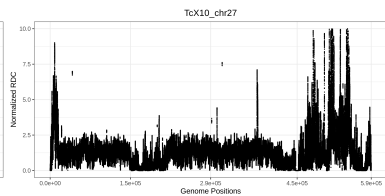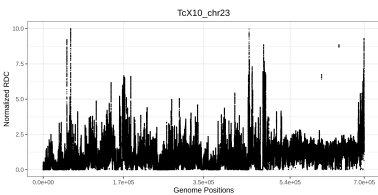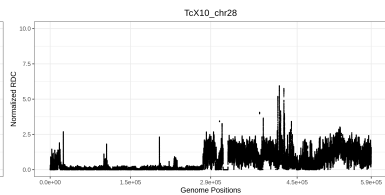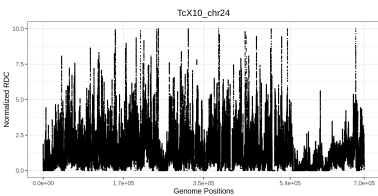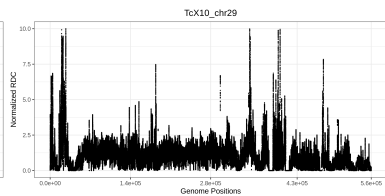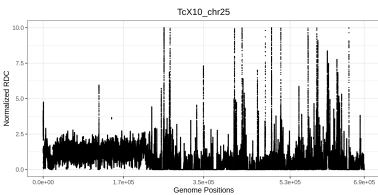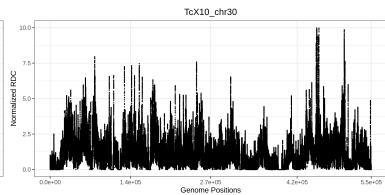

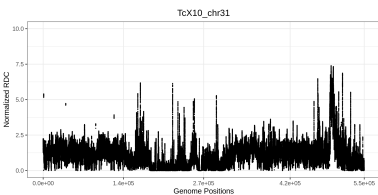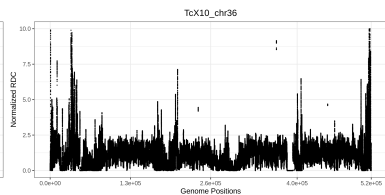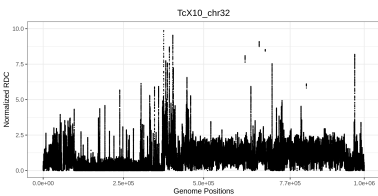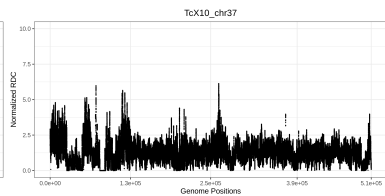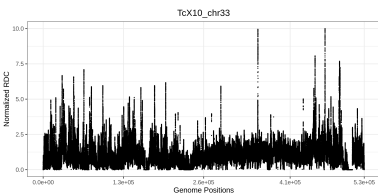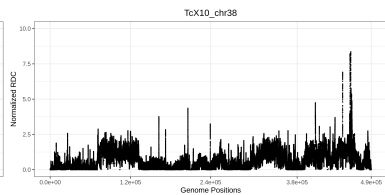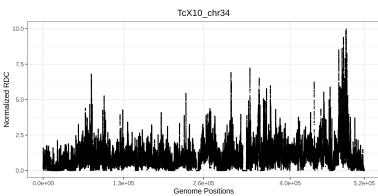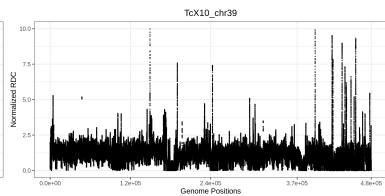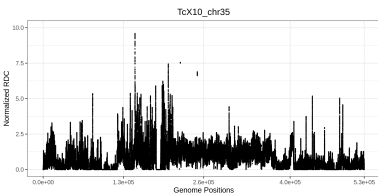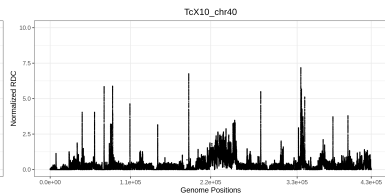

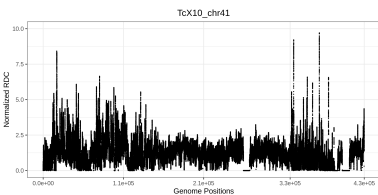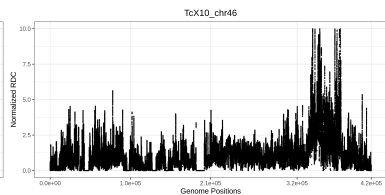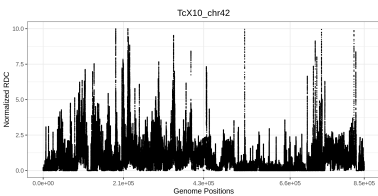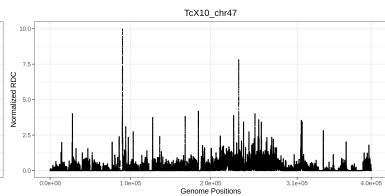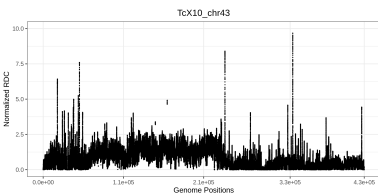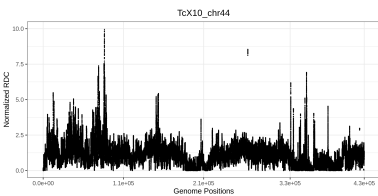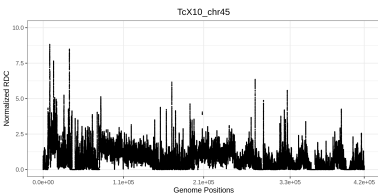

Supplement: Supplementary Figure 2 — Results from mapping Illumina reads back to the completed genome sequence. The plots show coverage across each chromosome. Local variation in coverage indicate the presence of highly repeated regions. [file DataSheet_7.pdf]

Supplementary fig. 3. CGI10

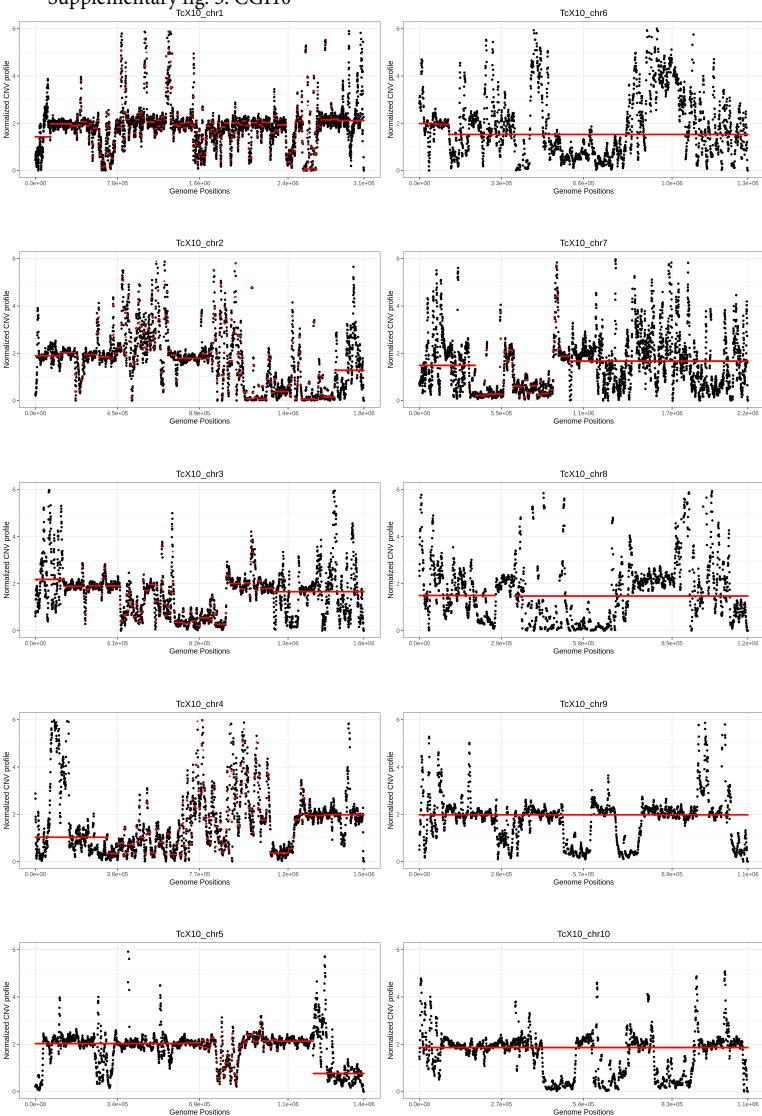

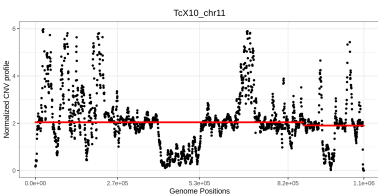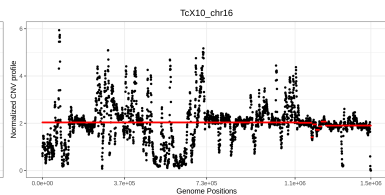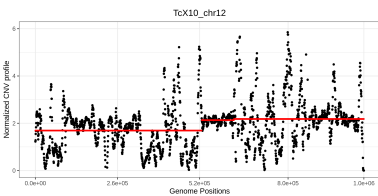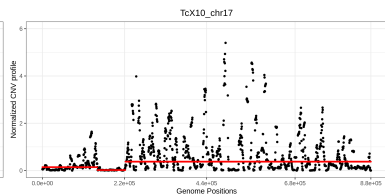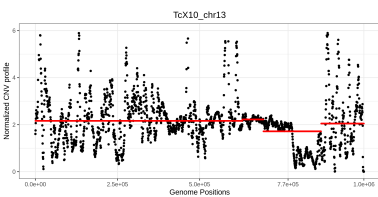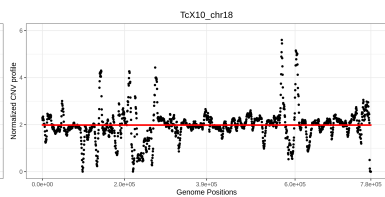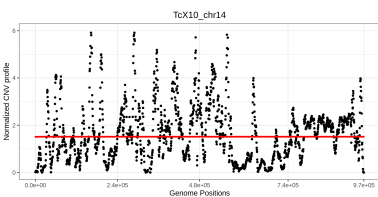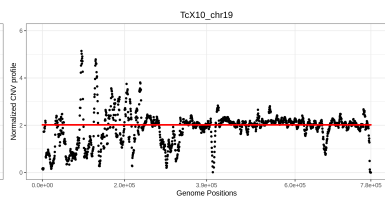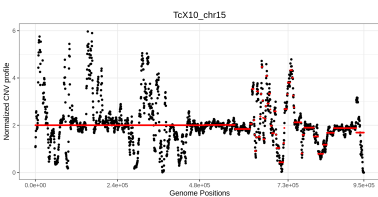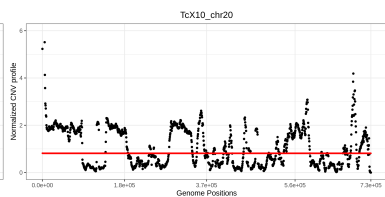

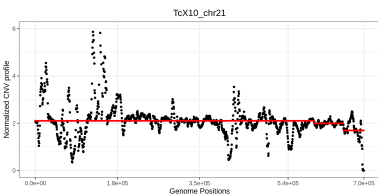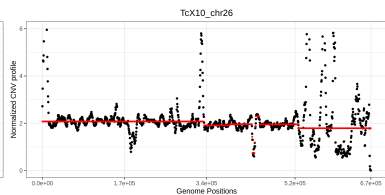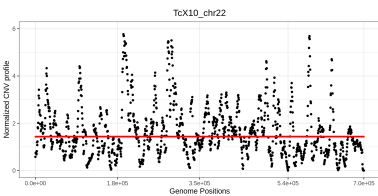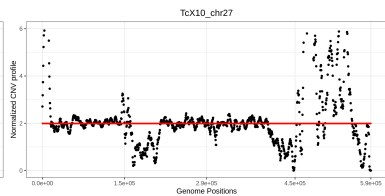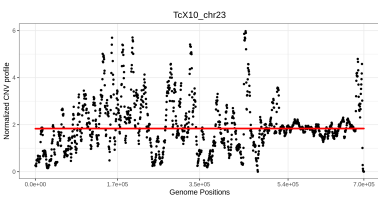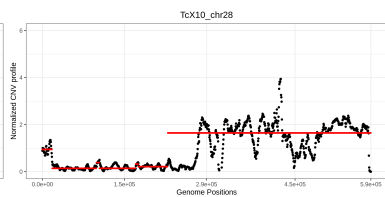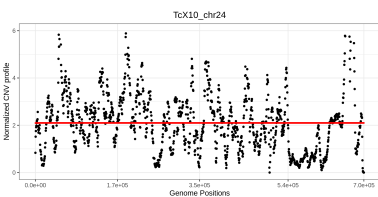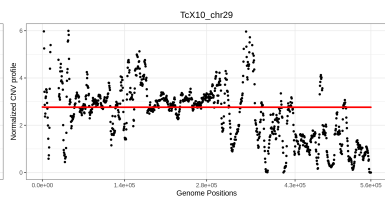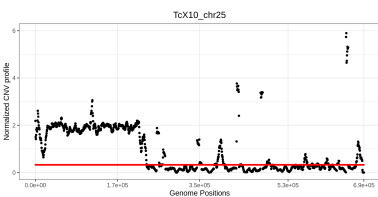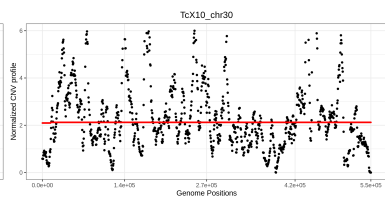

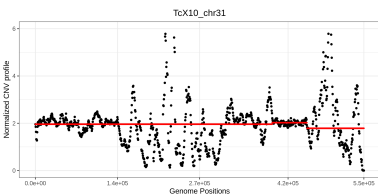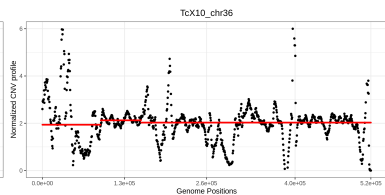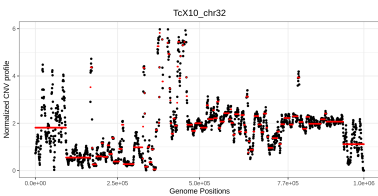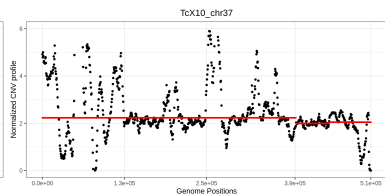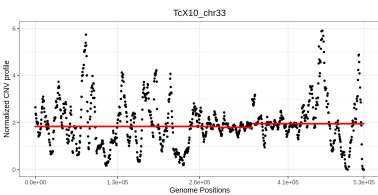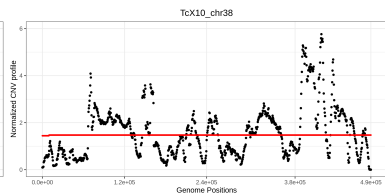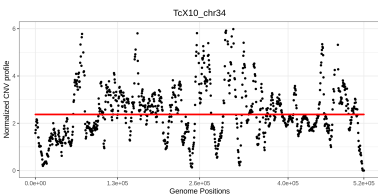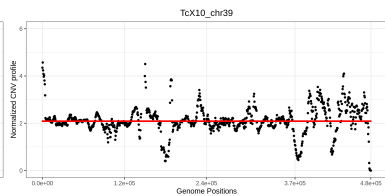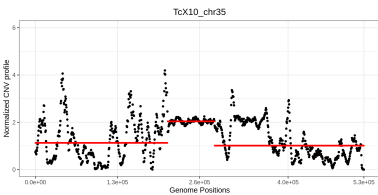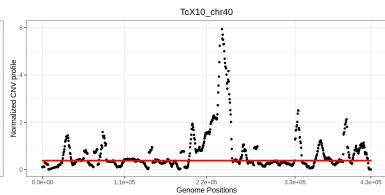

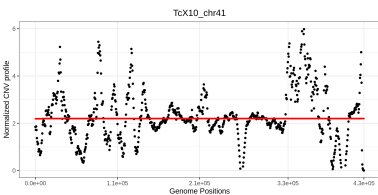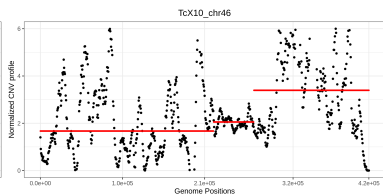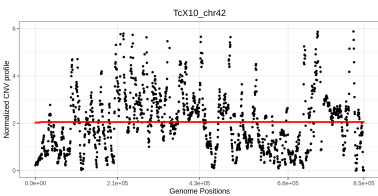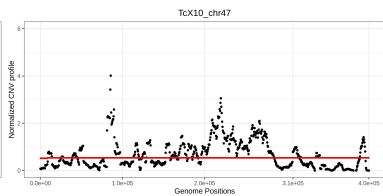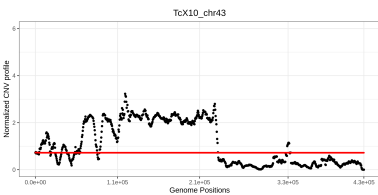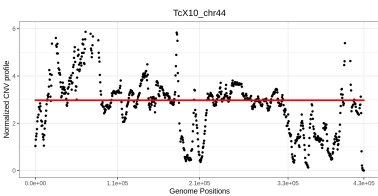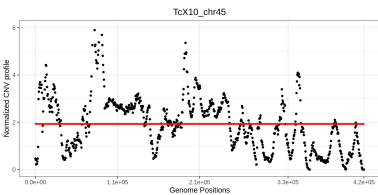

Supplement: Supplementary Figure 3–8 — Distribution of CNV changes in the Colombian CG clones and FcHc clones for all chromosomes. [file DataSheet_8.pdf]

Supplementary fig. 4. CGI11

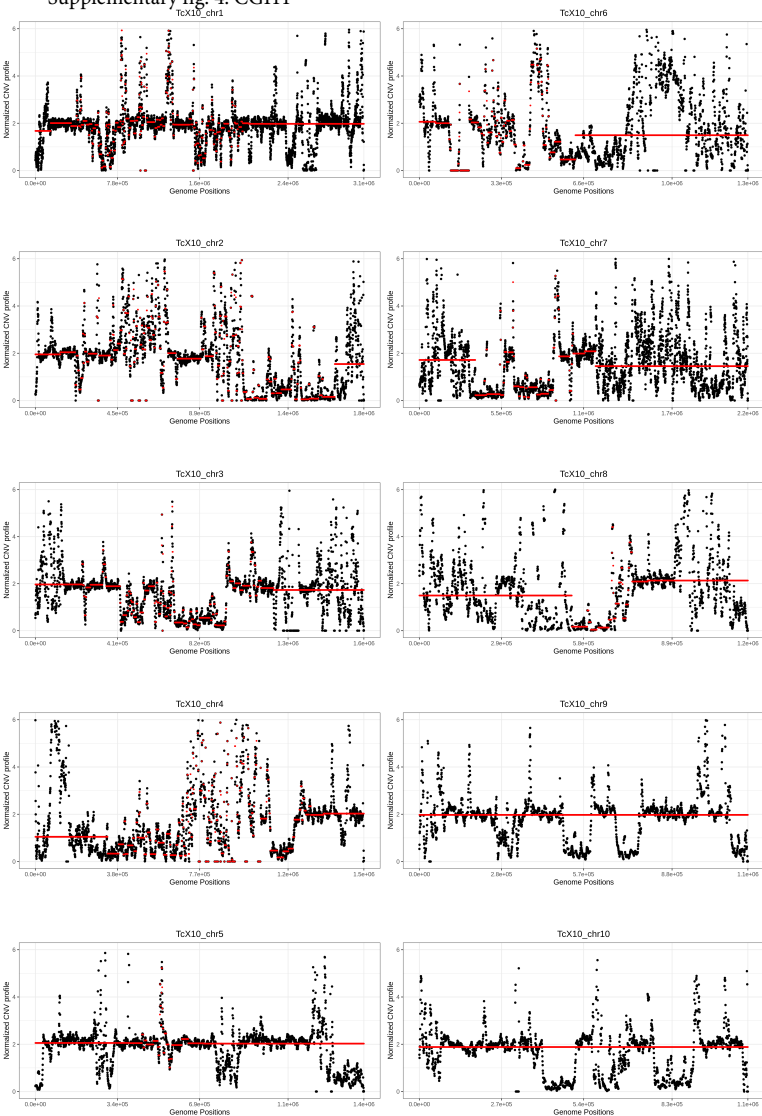

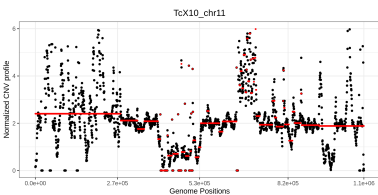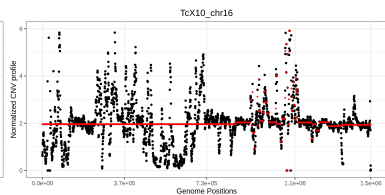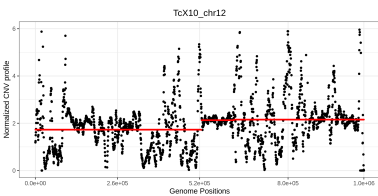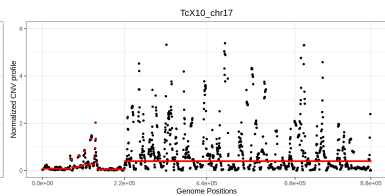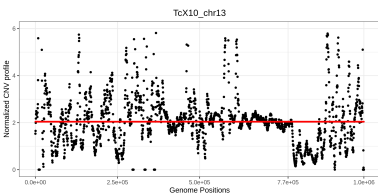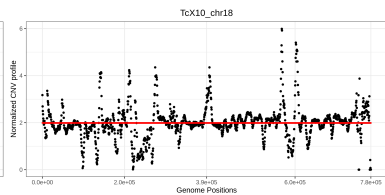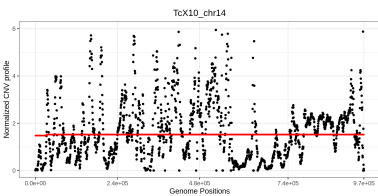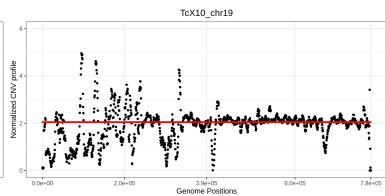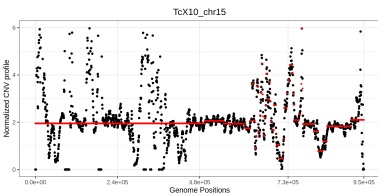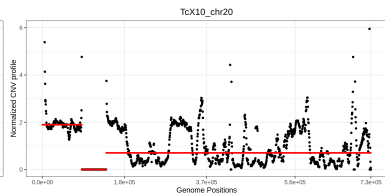

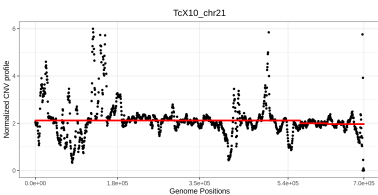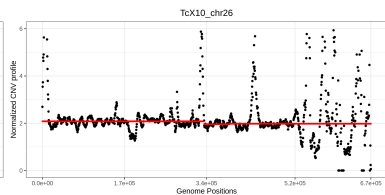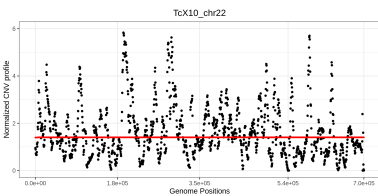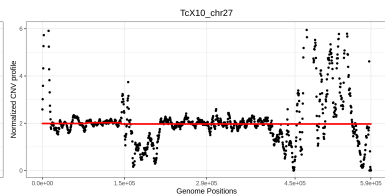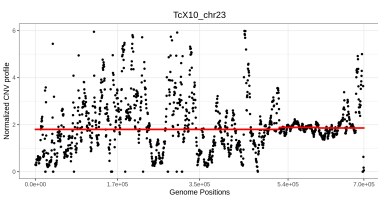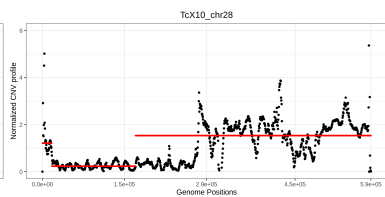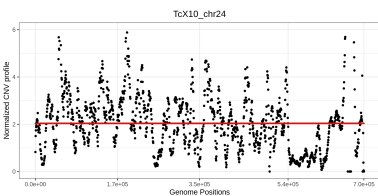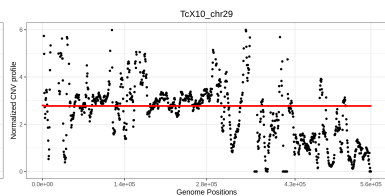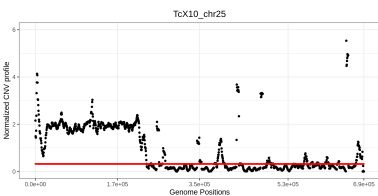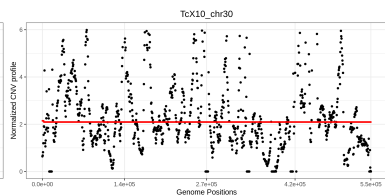

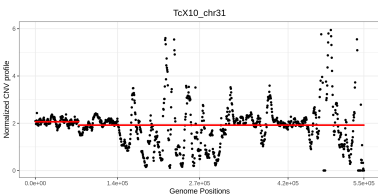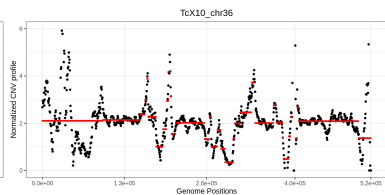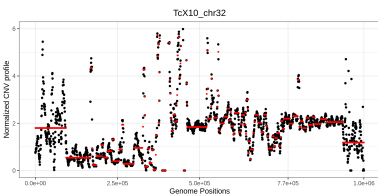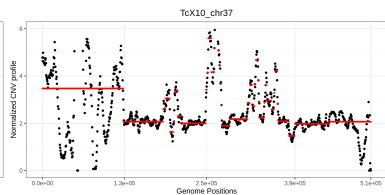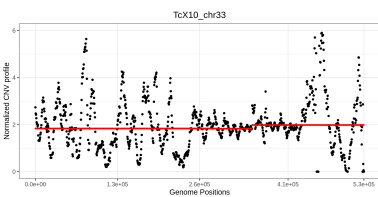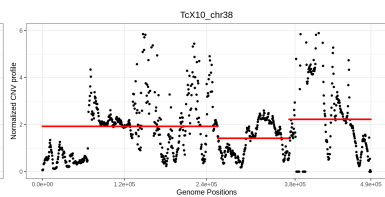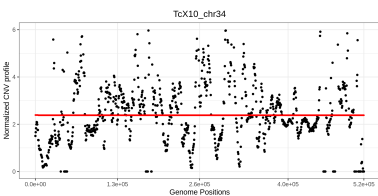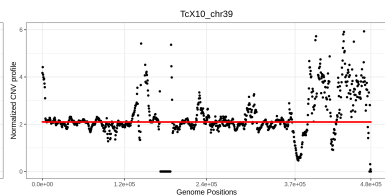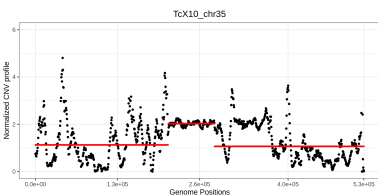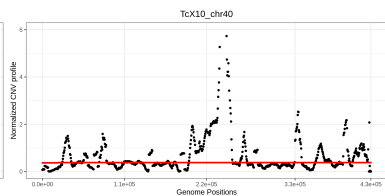

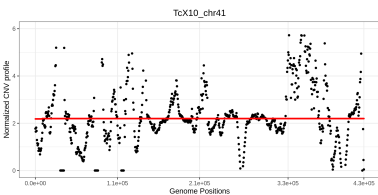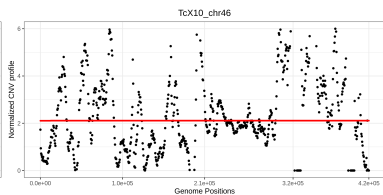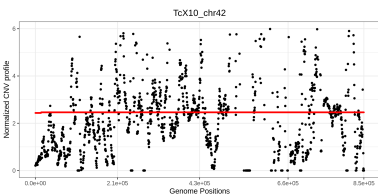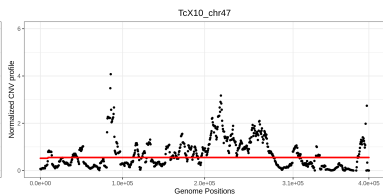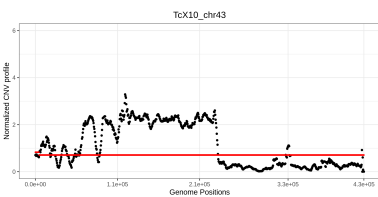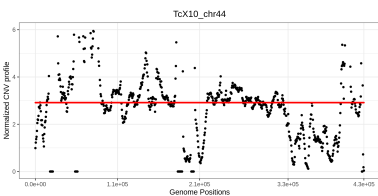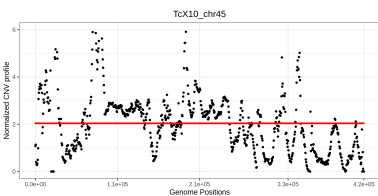

Supplement: Supplementary file 10 [file DataSheet_9.pdf]

Supplementary fig. 5. CGI13

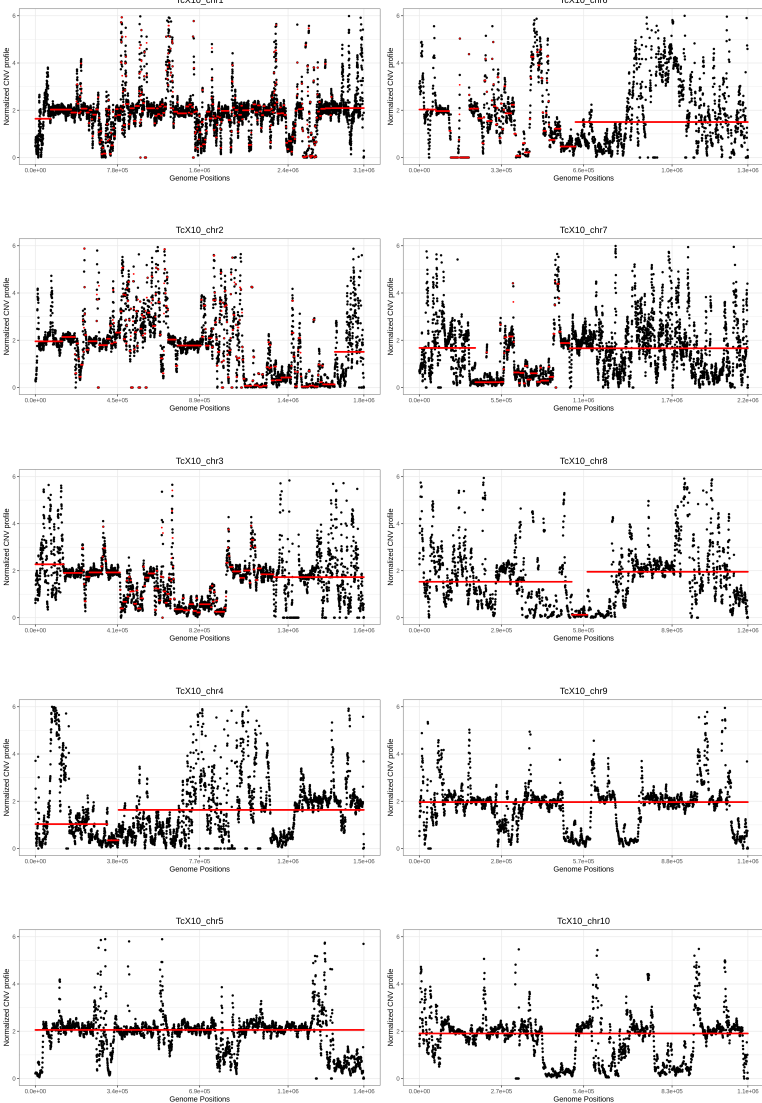

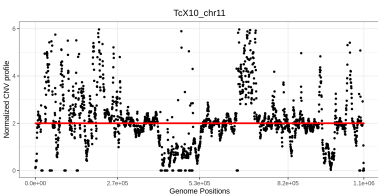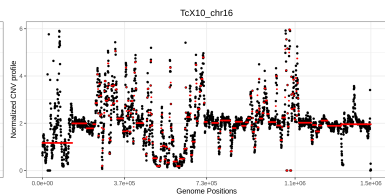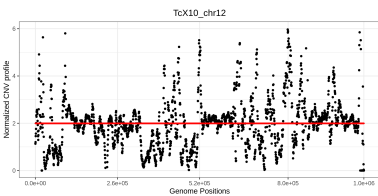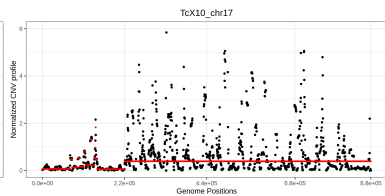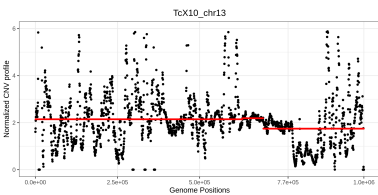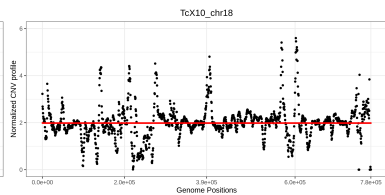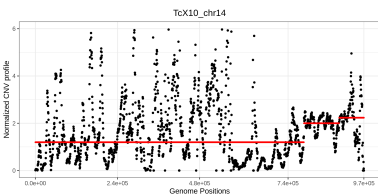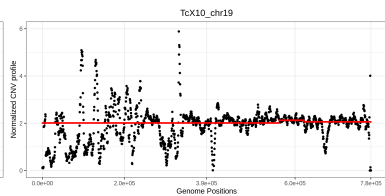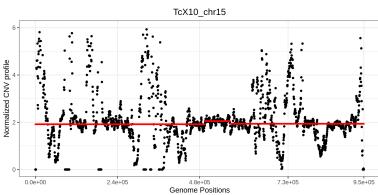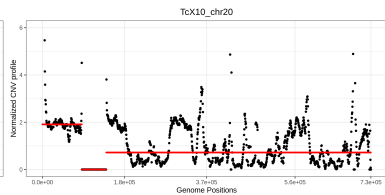

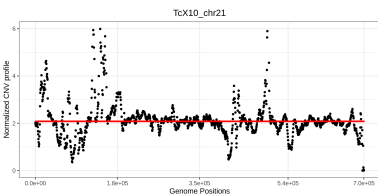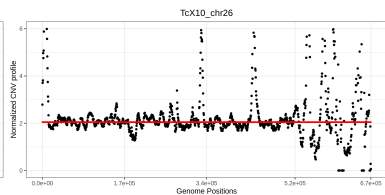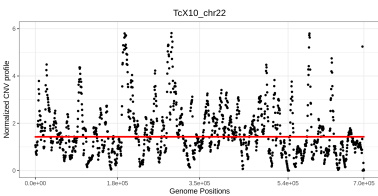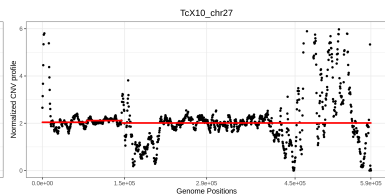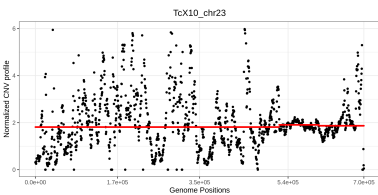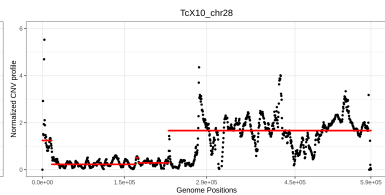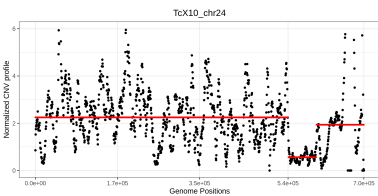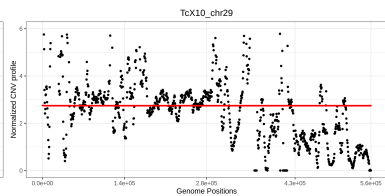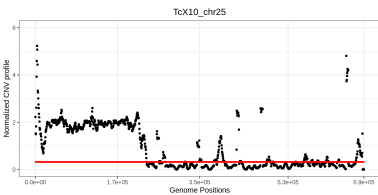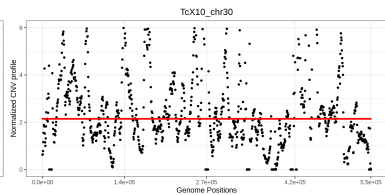

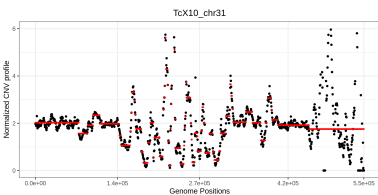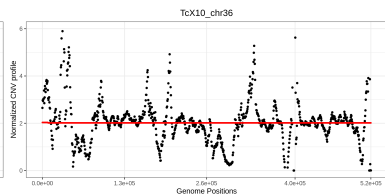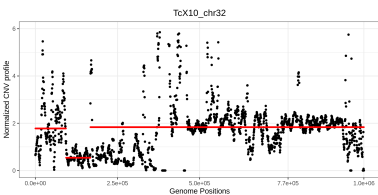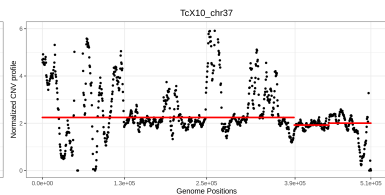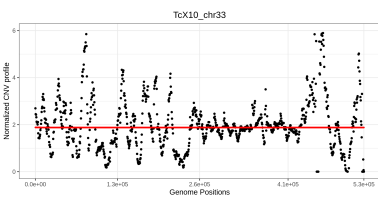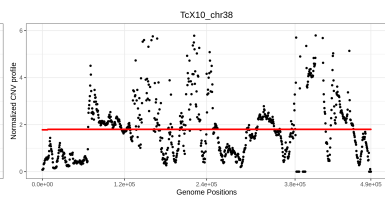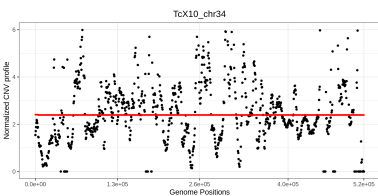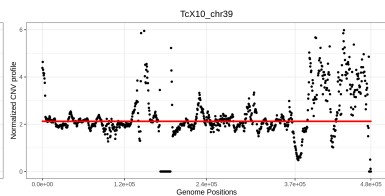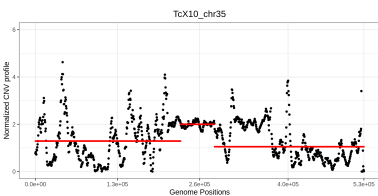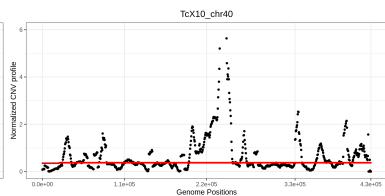

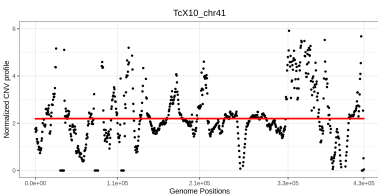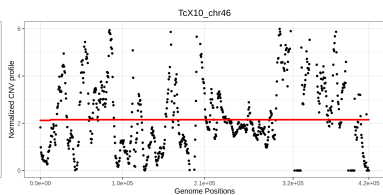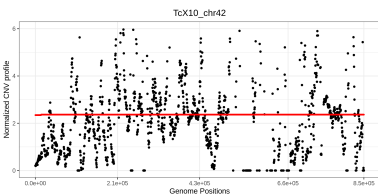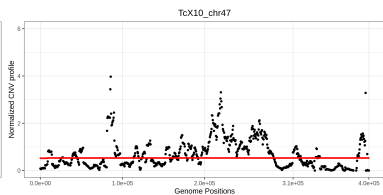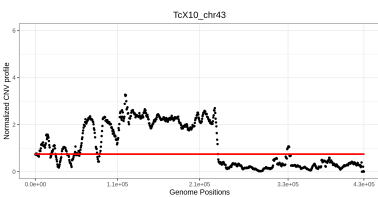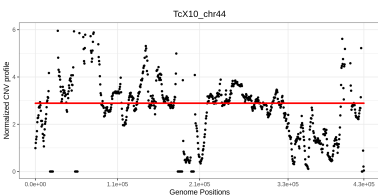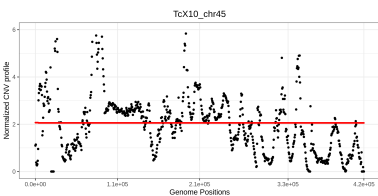

Supplement: Supplementary file 11 [file DataSheet_10.pdf]

Supplementary fig. 6. FcHc1

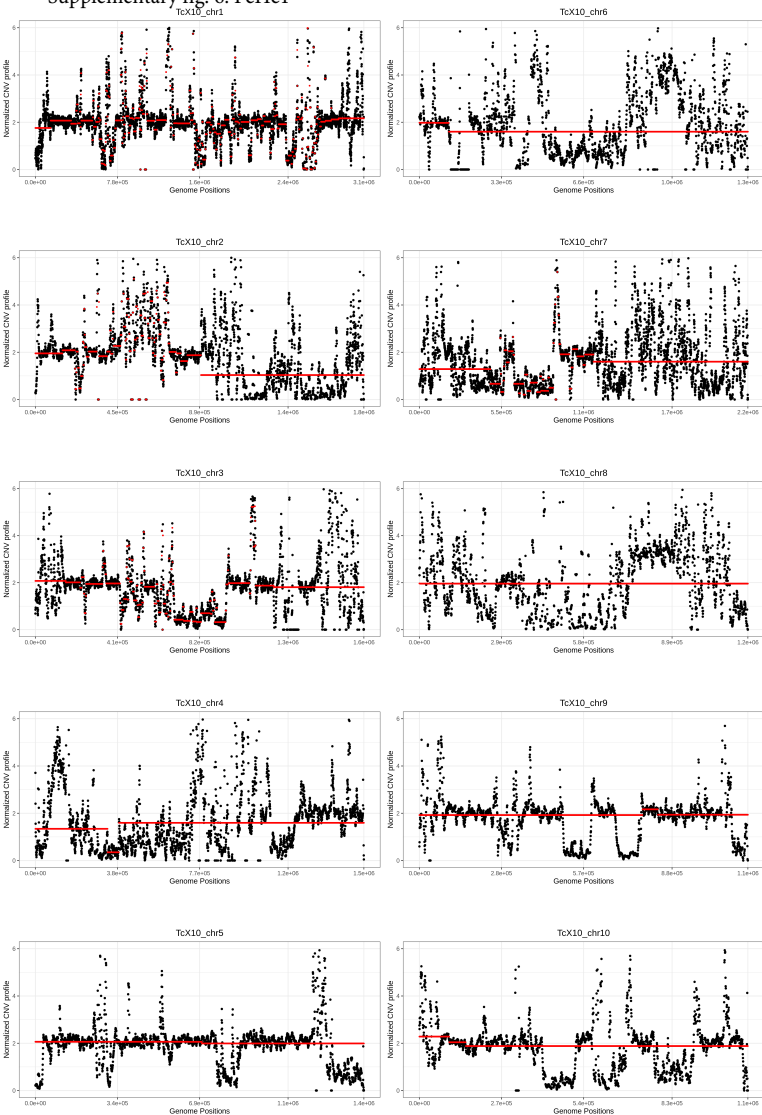

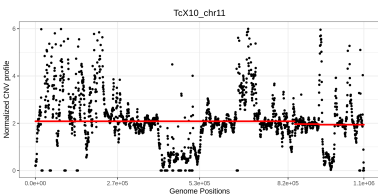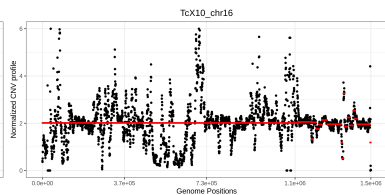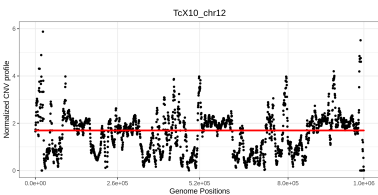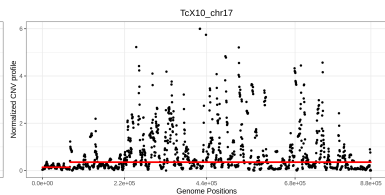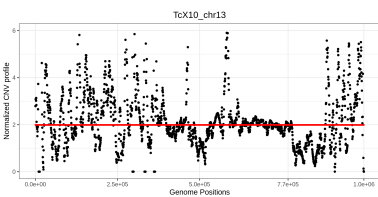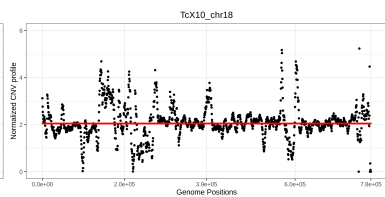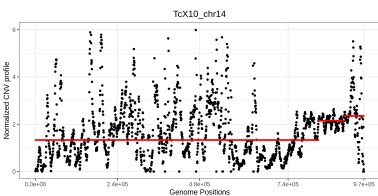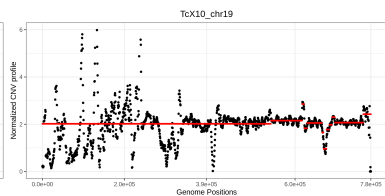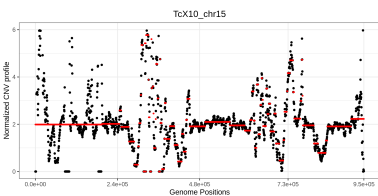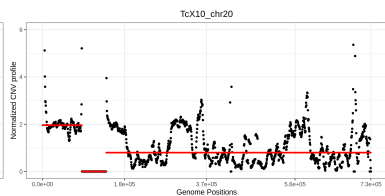

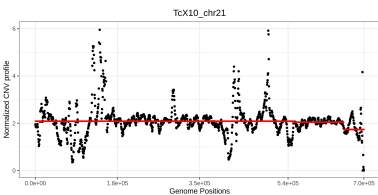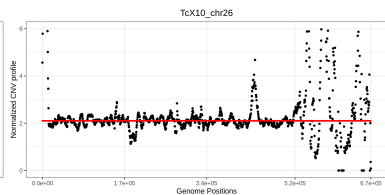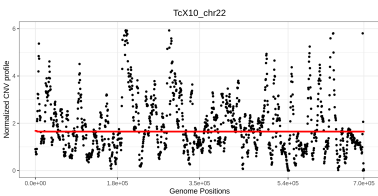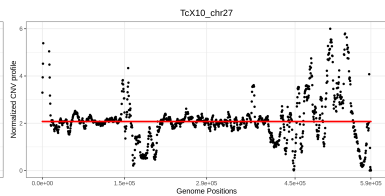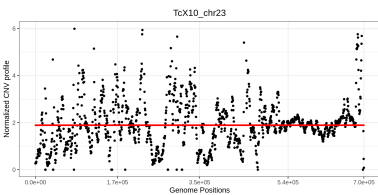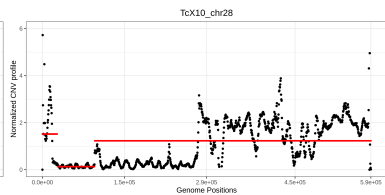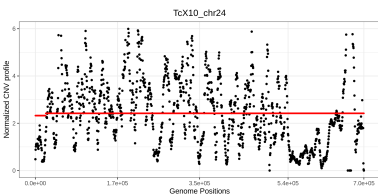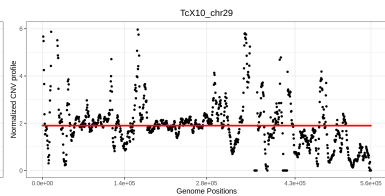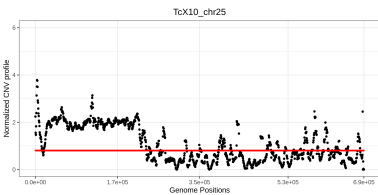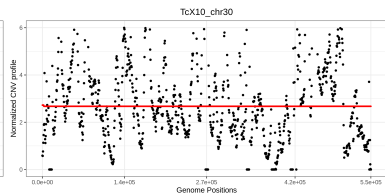

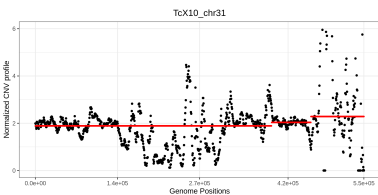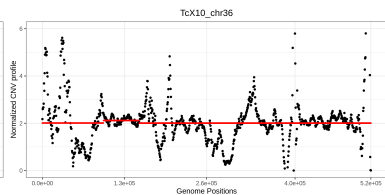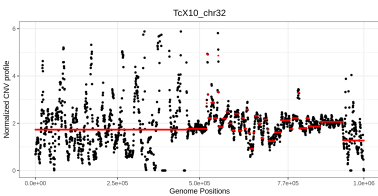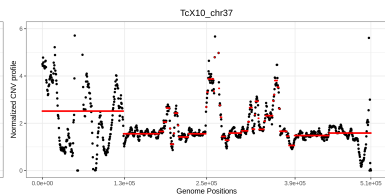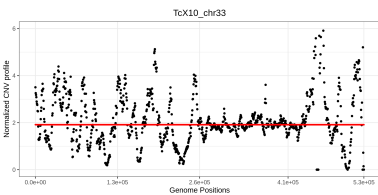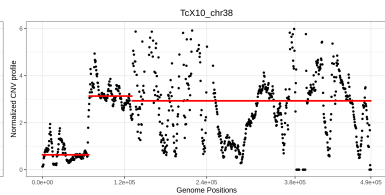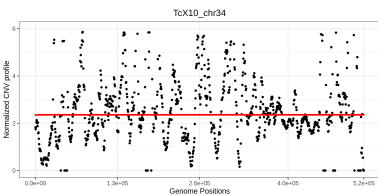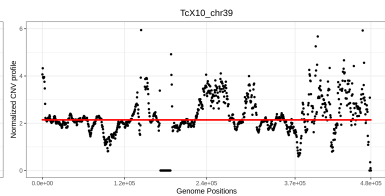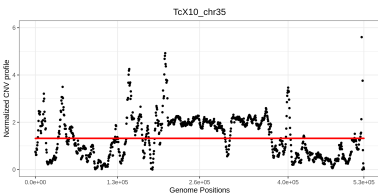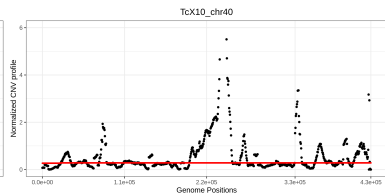

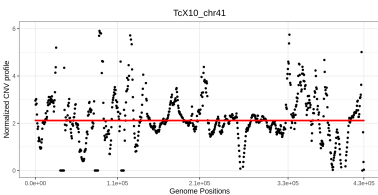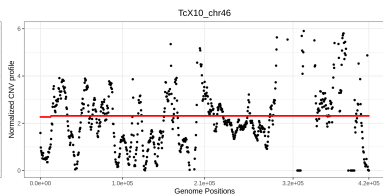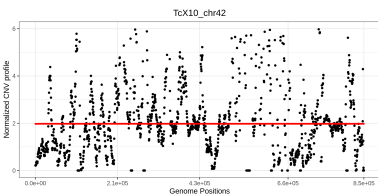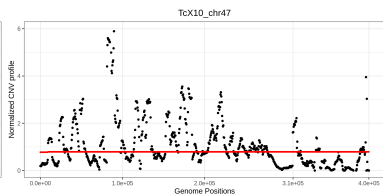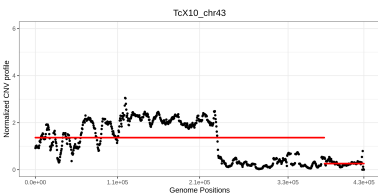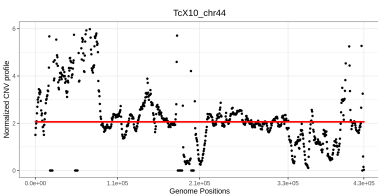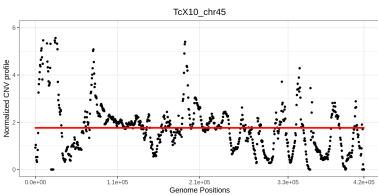

Supplement: Supplementary file 12 [file DataSheet_11.pdf]

Supplementary fig. 7. FcHc2

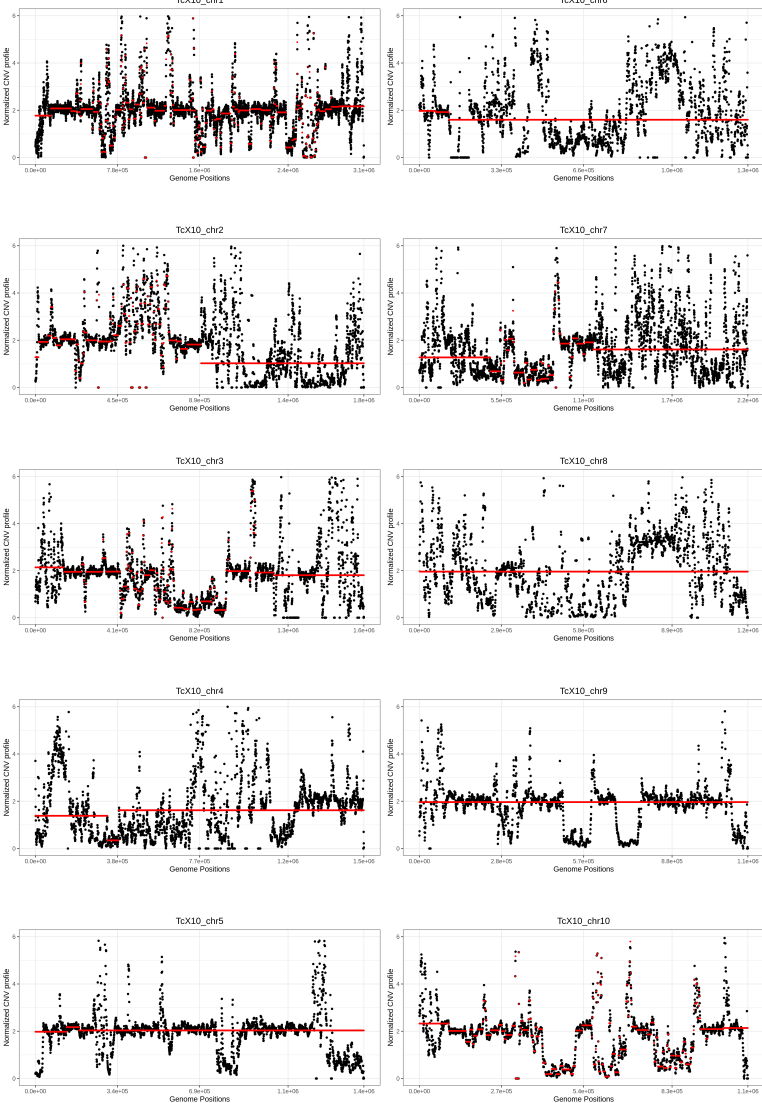

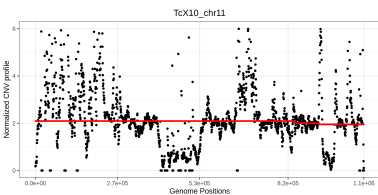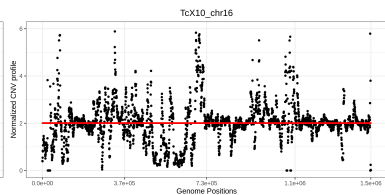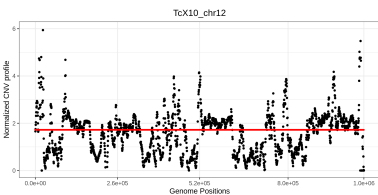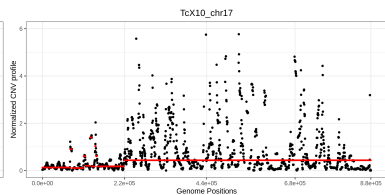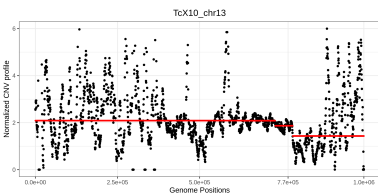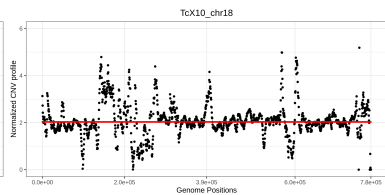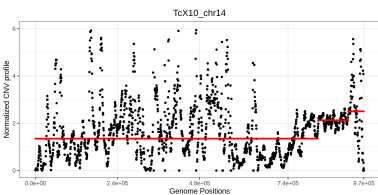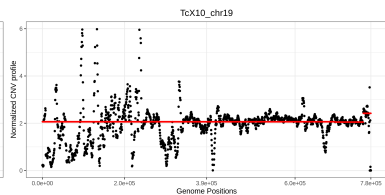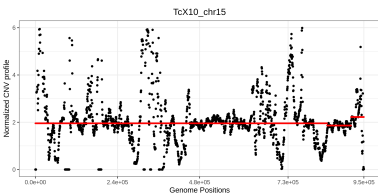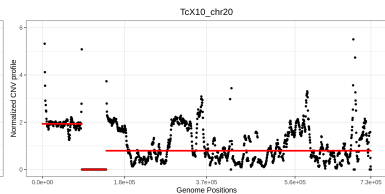

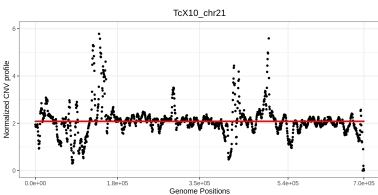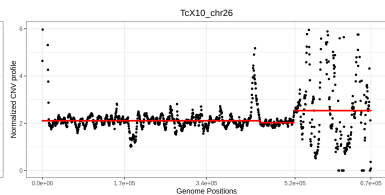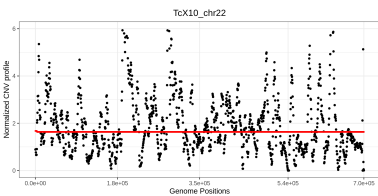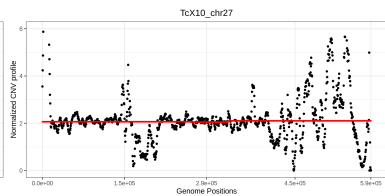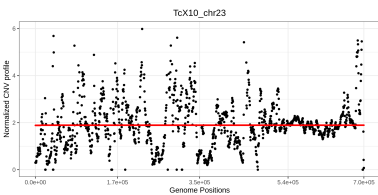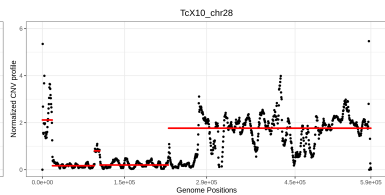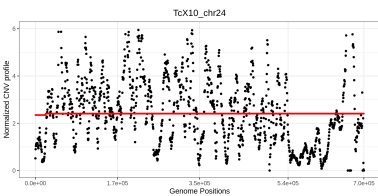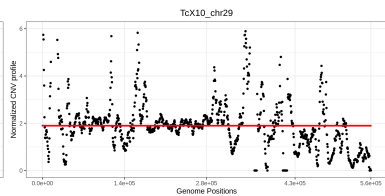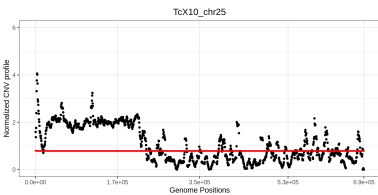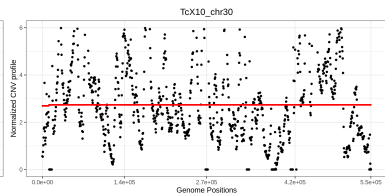

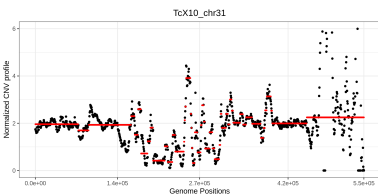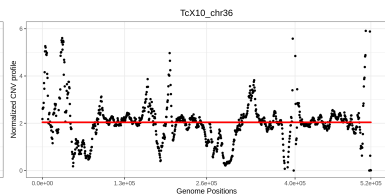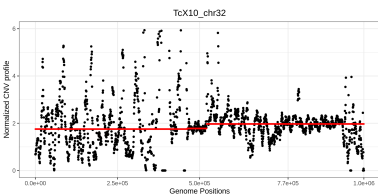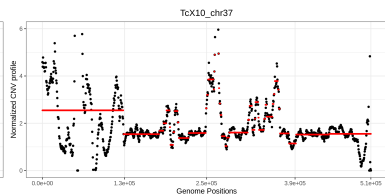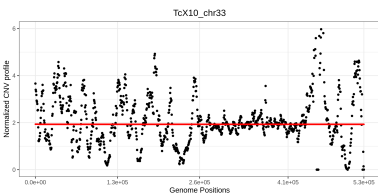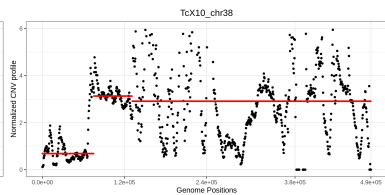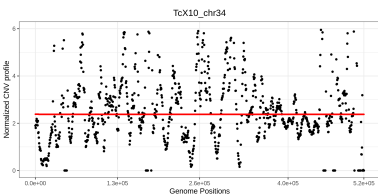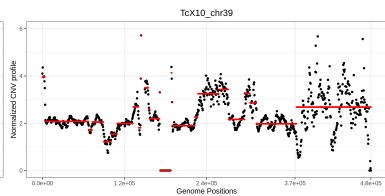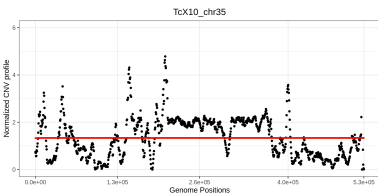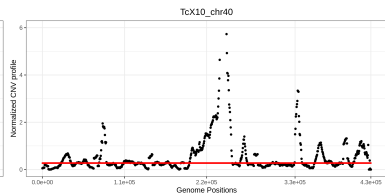

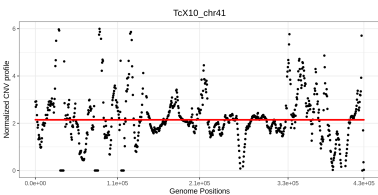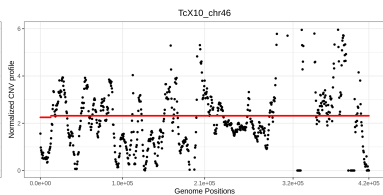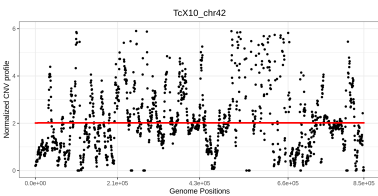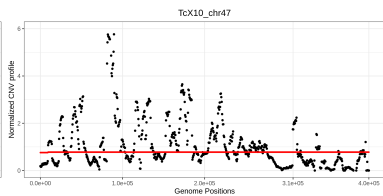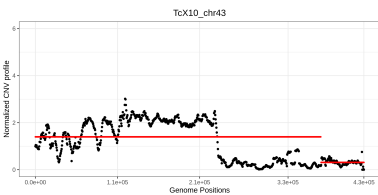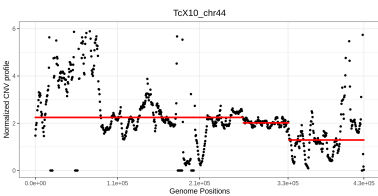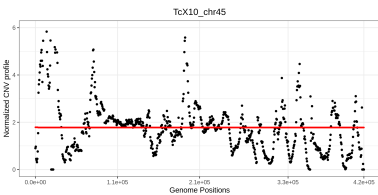

Supplement: Supplementary file 13 [file DataSheet_12.pdf]

Supplementary fig. 8. FcHc4

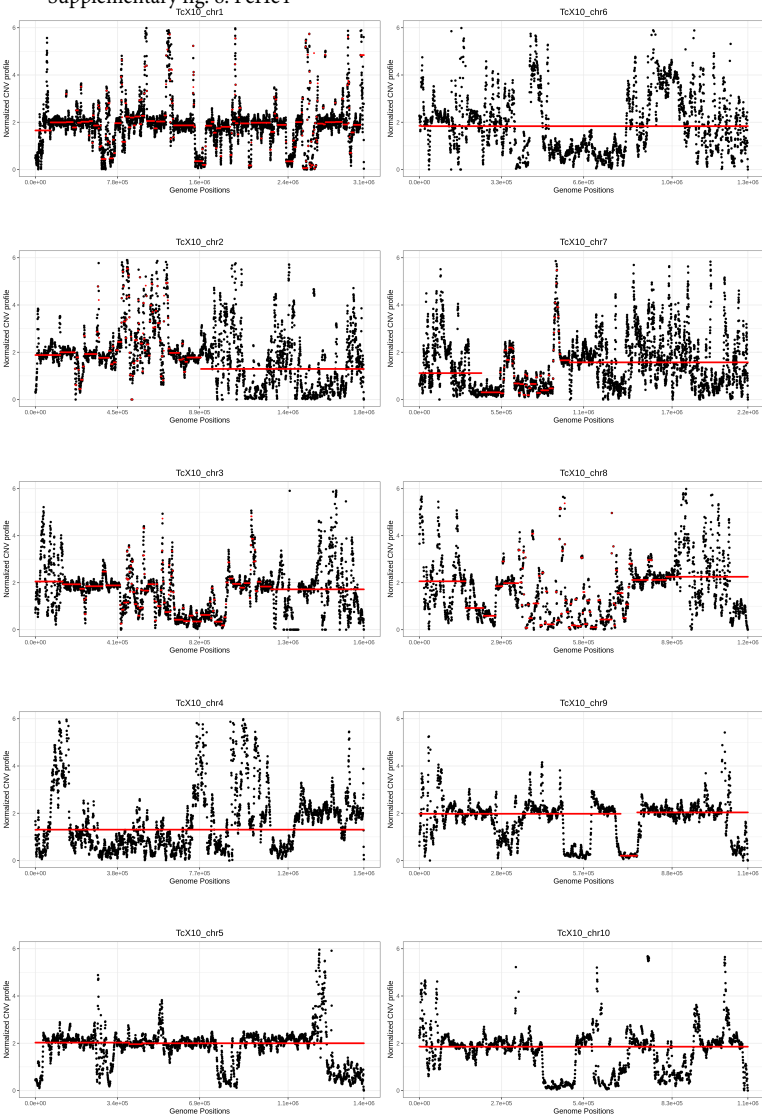

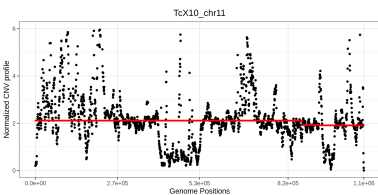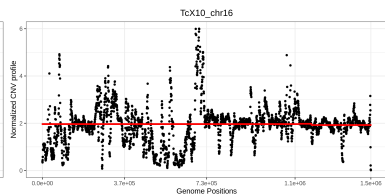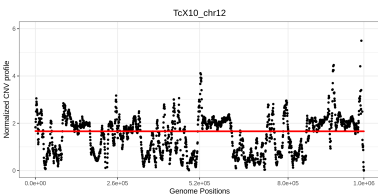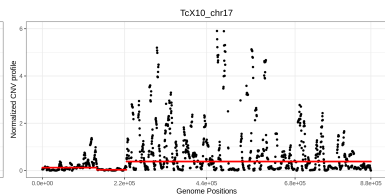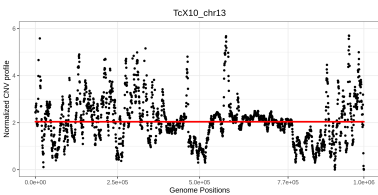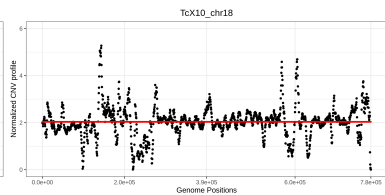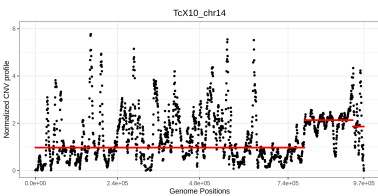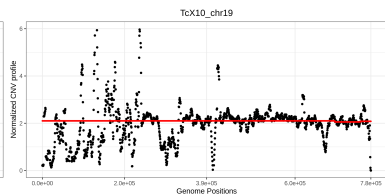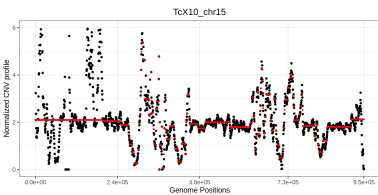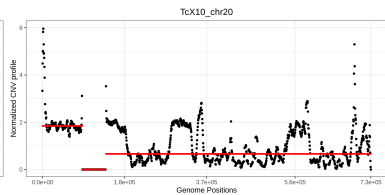

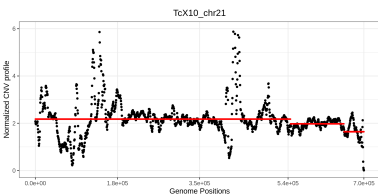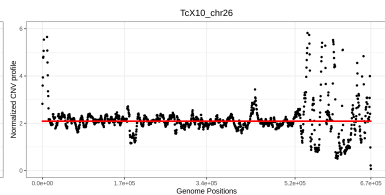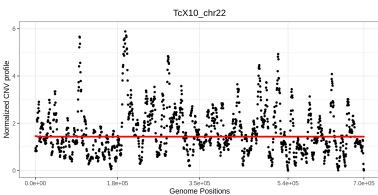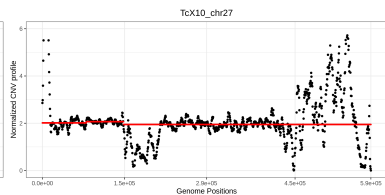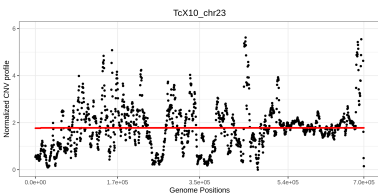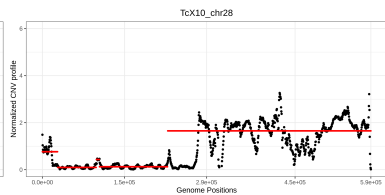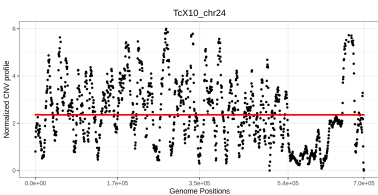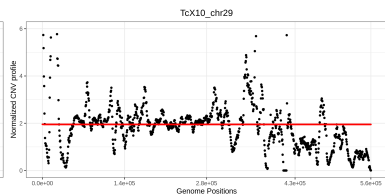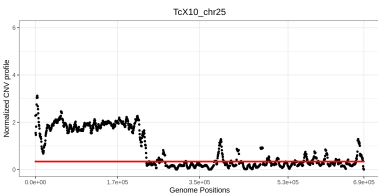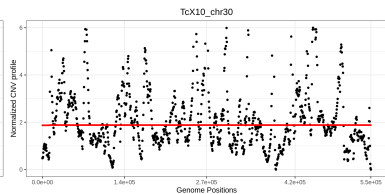

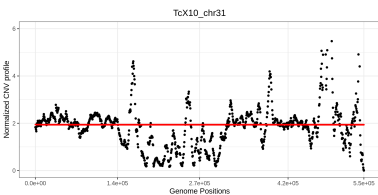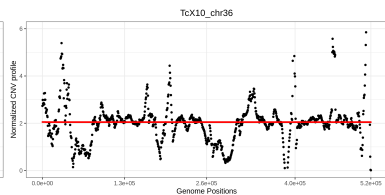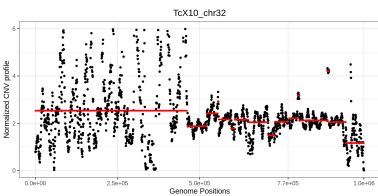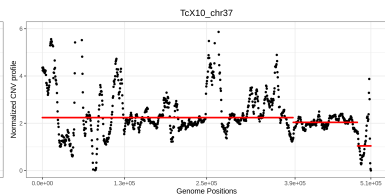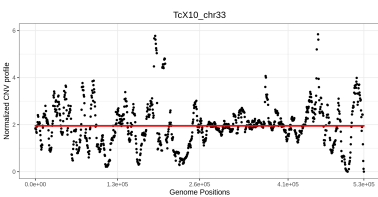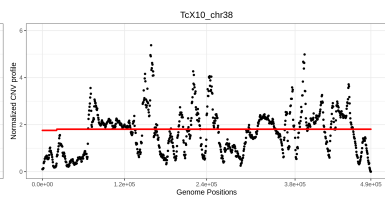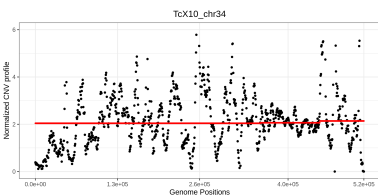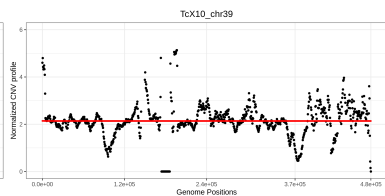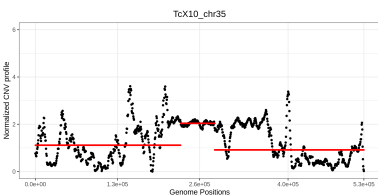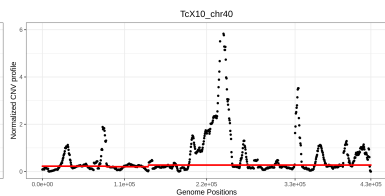

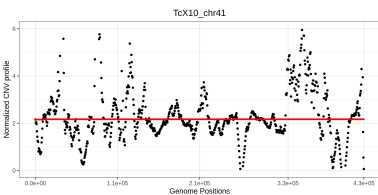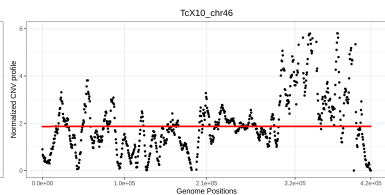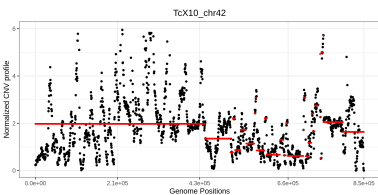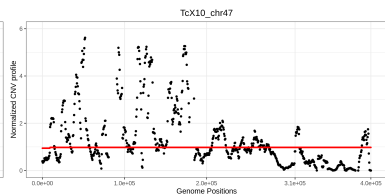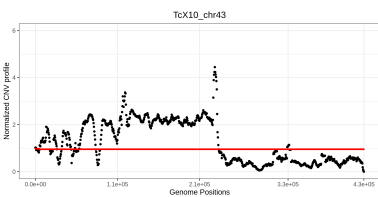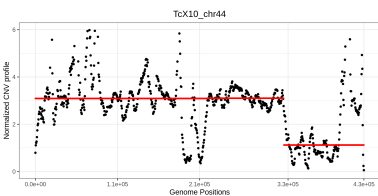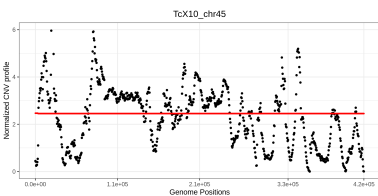

Supplement: Supplementary file 14 [file DataSheet_13.pdf]
